# Low nitrogen stress-induced transcriptome changes revealed the molecular response and tolerance characteristics in maintaining the C/N balance of sugar beet (*Beta vulgaris* L.)

**DOI:** 10.3389/fpls.2023.1164151

**Published:** 2023-04-21

**Authors:** Jiajia Li, Xinyu Liu, Lingqing Xu, Wangsheng Li, Qi Yao, Xilong Yin, Qiuhong Wang, Wenbo Tan, Wang Xing, Dali Liu

**Affiliations:** ^1^ National Beet Medium-term Gene Bank, Heilongjiang University, Harbin, China; ^2^ Key Laboratory of Sugar Beet Genetics and Breeding, Heilongjiang Province Common College/College of Advanced agriculture and ecological environment, Heilongjiang University, Harbin, China; ^3^ Key Laboratory of Molecular Biology, School of Life Sciences, Heilongjiang University, Harbin, China

**Keywords:** sugar beet, low nitrogen stress, transcriptome, photosynthesis, carbon and nitrogen balance, nitrogen assimilation and transport

## Abstract

Nitrogen (N) is an essential macronutrient for plants, acting as a common limiting factor for crop yield. The application of nitrogen fertilizer is related to the sustainable development of both crops and the environment. To further explore the molecular response of sugar beet under low nitrogen (LN) supply, transcriptome analysis was performed on the LN-tolerant germplasm ‘780016B/12 superior’. In total, 580 differentially expressed genes (DEGs) were identified in leaves, and 1,075 DEGs were identified in roots (log_2_^|FC|^ ≥ 1; q value < 0.05). Gene Ontology (GO), protein−protein interaction (PPI), and Kyoto Encyclopedia of Genes and Genomes (KEGG) analyses clarified the role and relationship of DEGs under LN stress. Most of the downregulated DEGs were closely related to “photosynthesis” and the metabolism of “photosynthesis-antenna proteins”, “carbon”, “nitrogen”, and “glutathione”, while the upregulated DEGs were involved in flavonoid and phenylalanine biosynthesis. For example, *GLUDB* (glutamate dehydrogenase B) was identified as a key downregulated gene, linking carbon, nitrogen, and glutamate metabolism. Thus, low nitrogen-tolerant sugar beet reduced energy expenditure mainly by reducing the synthesis of energy-consuming amino acids, which in turn improved tolerance to low nitrogen stress. The glutathione metabolism biosynthesis pathway was promoted to quench reactive oxygen species (ROS) and protect cells from oxidative damage. The expression levels of nitrogen assimilation and amino acid transport genes, such as *NRT2.5 * (high-affinity nitrate transporter), *NR* (nitrate reductase [NADH]), *NIR* (ferredoxin-nitrite reductase), *GS* (glutamine synthetase leaf isozyme), *GLUDB, GST* (glutathione transferase) and *GGT3* (glutathione hydrolase 3) at low nitrogen levels play a decisive role in nitrogen utilization and may affect the conversion of the carbon skeleton. *DFRA* (dihydroflavonol 4-reductase) in roots was negatively correlated with *NIR* in leaves (coefficient = −0.98, p < 0.05), suggesting that there may be corresponding remote regulation between “flavonoid biosynthesis” and “nitrogen metabolism” in roots and leaves. *FBP* (fructose 1,6-bisphosphatase) and *PGK* (phosphoglycerate kinase) were significantly positively correlated (p < 0.001) with Ci (intercellular CO_2_ concentration). The reliability and reproducibility of the RNA-seq data were further confirmed by real-time fluorescence quantitative PCR (qRT−PCR) validation of 22 genes (R^2^ = 0.98). This study reveals possible pivotal genes and metabolic pathways for sugar beet adaptation to nitrogen-deficient environments.

## 1 Introduction

The current world population is 7.7 billion, and China accounts for 1/5 of the total population (O’Sullivan, 2021). Increasing crop yield has been one of the key objectives to meet the needs of China’s growing population and global agricultural production. Nitrogen (N) is an important mineral element essential for plant life activities and a major limiting factor for crop yield, especially for sugar beet (*Beta vulgaris* L.). In both developed and developing countries, large amounts of nitrogen fertilizers are applied to meet crop demand (Zhang et al., 2015). However, such operations have led to serious biodiversity and environmental pollution problems and threaten human health (Xu et al., 2017; Ren et al., 2021). As an essential sugar and economic crop (Peleman and van der Voort, 2003; Simões et al., 2016), sugar beet is mainly distributed in northeast, northwest, and north China (Chen et al., 2017). N provides beets with nutrients, and proper N application has attracted increasing attention in recent years for high yield and sugar production of taproots.

In plants, N limitation causes leaf yellowing and plant biomass reduction. Low N application was found to reduce total plant nutrient accumulation and adversely affect yield (Pan et al., 2016). However, Yin et al. (2019) found that N deficiency optimized the distribution of N within wheat and therefore maintained or increased the yield of the grain. This indicates that nitrogen regulation and utilization is a complex process, and different plants in different nitrogen-deficient environments may have similar and different response mechanisms.

Photosynthesis is an important biological process that affects the accumulation of energy and dry matter in plants. The photosystem consists of two parts, photosystem II (PSII) and photosystem I (PSI), including a complex of proteins and photosynthetic pigments (e.g., chlorophyll) that absorb, transmit, and convert light energy. Transcriptome analysis revealed that low nitrogen stress reduced the expression levels of genes related to the apple antenna system, light absorption, and electron transfer (Wen et al., 2022). A reduction in photosynthesis ultimately leads to a decrease in biomass production and yield (Shi et al., 2022). Glycolysis, the pentose phosphate pathway (PPP), and the tricarboxylic acid (TCA) cycle are the major metabolic pathways in plants and are influenced by nitrogen supply, providing energy and carbon skeletons for other metabolic pathways. Carbon (C) metabolism and nitrogen metabolism in plants are closely coordinated, and their balance is crucial.

The C metabolism and N metabolism of plants are closely coordinated with each other (Bao et al., 2015) and require a common reducing power, ATP, and carbon skeleton (Sweetlove et al., 2010). Under stress conditions, they exhibit complex and diverse responses to abiotic stresses by altering gene and protein expression as well as primary and secondary metabolites (Bhatotia et al., 2015; Onaga and Wydra, 2016). During nitrogen starvation, various defective response genes act to maintain plant survival by altering root architecture (Li et al., 2023), improving nitrogen assimilation (Imamura et al., 2009), and increasing lignin content (Tian et al., 2022). The phenylpropanoid metabolic pathway is closely related to lignin synthesis (Cong et al., 2013), whose pathway is closely related to photosynthetic carbon utilization (Zhang et al., 2018c). Phenylpropanoid polymers such as lignin are mechanical supports for plant growth and facilitate the long-distance transport of water and nutrients (Zhao, 2016). Studies have shown that the enzymatic activities of *PAL*, *4CL*, *CAD*, and *POD* and their corresponding gene expression in lignified tissues affect lignin biosynthesis in bamboo shoots, indicating that these enzymes play key roles in lignin biosynthesis (Zheng et al., 2020). Naringenin is a member of the flavonoid family and is the starting point for the synthesis of a variety of other flavonoid molecules. Drought stress induces the expression of the flavonoid structural genes *CHS*, *CHI*, *FLS*, and *DFR*, which leads to the accumulation of flavonoids such as anthocyanins and improves the tolerance of ginkgo to drought (Yu et al., 2022). Plant flavonoid compounds, especially anthocyanins and rutin, also play a crucial role under nitrogen deficiency conditions (Shen et al., 2022). Carbon metabolism provides the carbon source and energy for nitrogen metabolism (Nunes-Nesi et al., 2010), while nitrogen metabolism provides enzymes and photosynthetic pigments for carbon metabolism. Glutamate is used for the synthesis of N compounds such as proteins and nucleic acids. When the GS/GOGAT cycle assimilates NH_3_, the carbon skeleton needs to be continuously replenished to meet the demand for N associated with plant growth (Liao et al., 2022). Thus, glutamate metabolism can directly link nitrogen to carbon metabolism.

In recent years, continuous progress in sequencing technologies has provided an efficient way to identify functional genes and study their regulatory mechanisms, and the sugar beet genome has also been extensively developed (Dohm et al., 2014; Wascher et al., 2022). Previously, we explored the tolerance and adaptation characteristics of different sugar beet germplasms at both morphological and physiological levels under low N (LN) environments (Li et al., 2022; Li et al., 2023). However, there are still some important phenomena in this process that need to be explained through relevant molecular regulation. Thus, in this study, the sugar beet low N-tolerant germplasm ‘780016B/12 superior’ was subjected to both low N and normal N conditions. Using RNA-seq, we attempted to identify possible metabolic pathways and clarify the functions of candidate genes of sugar beet involved in the response to nitrogen deficiency. This study will help to improve the understanding of the mechanism of the sugar beet response to low N stress and provide a theoretical basis for proper N utilization in sugar beet.

## 2 Materials and methods

### 2.1 Plant materials and growing conditions

The experiment was conducted at the National Beet Medium-Term Gene Bank (Harbin, China) using ‘780016B/12 superior’ sugar beet germplasm as the test material. The intact and uniform seeds were sterilized in ethanol (75%) for 1 min and then washed three times in distilled water. Subsequently, they were sown in seedling trays containing vermiculite. After 9 days of cultivation, seedlings with consistent growth were selected and grown in Hoagland nutrient solution. There were two N-level treatments, LN treatment (0.5 mmol/L N) and normal N (CV, 5 mmol/L N), which were adjusted by the dose of KNO_3_ and Ca(NO_3_)_2_ in the modified Hoagland nutrient solution. Seedlings with two pairs of fully expanded leaves (approximately 2 weeks) were subjected to LN. After 12 h of treatment, samples were immediately frozen in liquid nitrogen and then refrigerated at −80°C until RNA was extracted. The hydroponic conditions were 25°C/18°C (day/night) with a 14 h light/10 h dark cycle, 45%–55% relative humidity, and a light intensity of 200 μmol/(m^2^·s^−1^). Each treatment was repeated three times.

### 2.2 Determination of physiological indexes

A CI-340 photosynthesis instrument (Sidi Ecological Instrument Co., Ltd., Beijing, China) was used to determine the net photosynthetic rate (Pn), transpiration rate (Tr), stomatal conductivity (Gs), and intercellular CO_2_ concentration (Ci). The second pair of fully expanded true leaves of sugar beet seedlings was measured. Five seedlings of each treatment were dried, and the roots and leaves were ground separately into powder. First, 0.1 g of the sample was digested with potassium sulfate and copper sulfate as catalysts and concentrated sulfuric acid (sulfuric acid), and the nitrogen content was determined using a Hanon K1100 automatic Kjeldahl nitrogen tester (Hanon Instrument Co., Ltd., Shandong, China). The N content (N uptake) was presented as mg per plant.

Correlations between nitrogen-stressed photosynthetic indexes and differentially expressed genes (DEGs) under LN were analyzed using Pearson’s correlation statistics. Independent samples t-tests were performed using SPSS statistical software (version 26.0, SPSS, Chicago, IL, USA). All analyses were performed with p <0.05, 0.01, or 0.001 as the significance levels.

### 2.3 Illumina sequencing, data analysis, and DEG annotation

After the determination of RNA concentration, purity, and integrity, the cDNA libraries were sequenced using the Illumina high-throughput sequencing platform (NovaSeq 6000, San Diego, CA, USA). The raw reads with low quality, junction contamination, and high content of unknown bases were filtered to obtain clean reads and analyzed for guanine–cytosine (GC) content, Q20, and Q30. HISAT2 was used to align clean reads to the sugar beet reference genome RefBeet-1.2.2. Fragments per kilobase per million fragments localized transcripts (FPKM) values were calculated for each gene in the samples using StringTie software to quantify its expression abundance and variation. EdgeR software (available online: http://www.r-project.org/) was used to screen for DEGs q value <0.05, log_2_^|FC|^ ≥ 1) (Cosgrove, 2016). Functional enrichment of DEGs was determined using the Gene Ontology (GO) (available online: http://www.r-project.org/) and Kyoto Encyclopedia of Genes and Genomes (KEGG) database (available online: http://kobas.cbi.pku.edu.cn/). We used p-values calculated by hypergeometric tests and corrected by FDR with FDR ≤0.05 as the threshold to identify significant functional categories and metabolic pathways.

### 2.4 Protein interaction network construction

The protein−protein interaction (PPI) network of DEG-encoded proteins identified by transcriptomic analysis was constructed using the STRING v11.5 online database (https://string-db.org/). PPI networks were constructed by targeting upregulated and downregulated DEGs. The core genes were further screened using Cytoscape software v3.9.1 (http://www.cytoscape.org/). The nodes represent proteins, and the connected edges illustrate the relevant interactions between proteins in the PPI network.

### 2.5 Bioinformatics analysis

The amino acid sequences of sugar beet BvGLUDB (BVRB_3g056180) and BvDFRA (BVRB_1g007170) were used to identify homologous sequences in the NCBI database. The alignment and domain analyses of proteins were performed using Espript3.

### 2.6 Real-time fluorescence quantitative PCR

Total RNA was extracted from sugar beet roots and leaves using RNA-easy Isolation Reagent (Vazyme R701, Nanjing Novozymes Biotech Co., Nanjing, China). RNA concentration and the OD_260_/OD_230_ and OD_260_/OD_280_ were determined. The cDNA was reverse transcribed according to the TranScript One-Step gDNA Removal and cDNA Synthesis SuperMix protocol (AT311, Beijing All Style Gold Biotechnology Co., Beijing, China). qPCR was performed on a real-time fluorescence quantitative PCR instrument (Thermo Fisher Scientific Instruments, Shanghai, China; QuantStudio™ 1 Plus) using the SuperReal PreMix Plus kit (Tiangen Biochemical Technology Co., Beijing, China; version FP210831). The PCR procedure was as follows: predenaturation at 95°C for 15 min; 40 cycles of denaturation at 95°C for 10 s, annealing at 60°C for 20 s, and extension at 72°C for 32 s; and a melting curve at 65°C–95°C. *BvGAPDH* (NC_ 024800) was selected as the internal reference gene. The primers were designed using the NCBI database tool (**Supplementary Table 1**). The real-time fluorescence quantitative PCR (qRT−PCR) analysis was repeated three times for each sample. Correlation coefficients of RNA-seq and qRT−PCR data were plotted and calculated using Origin (version 2021) graphing software.

## 3 Results

### 3.1 Transcriptome sequencing and alignment

**Figure 1:**
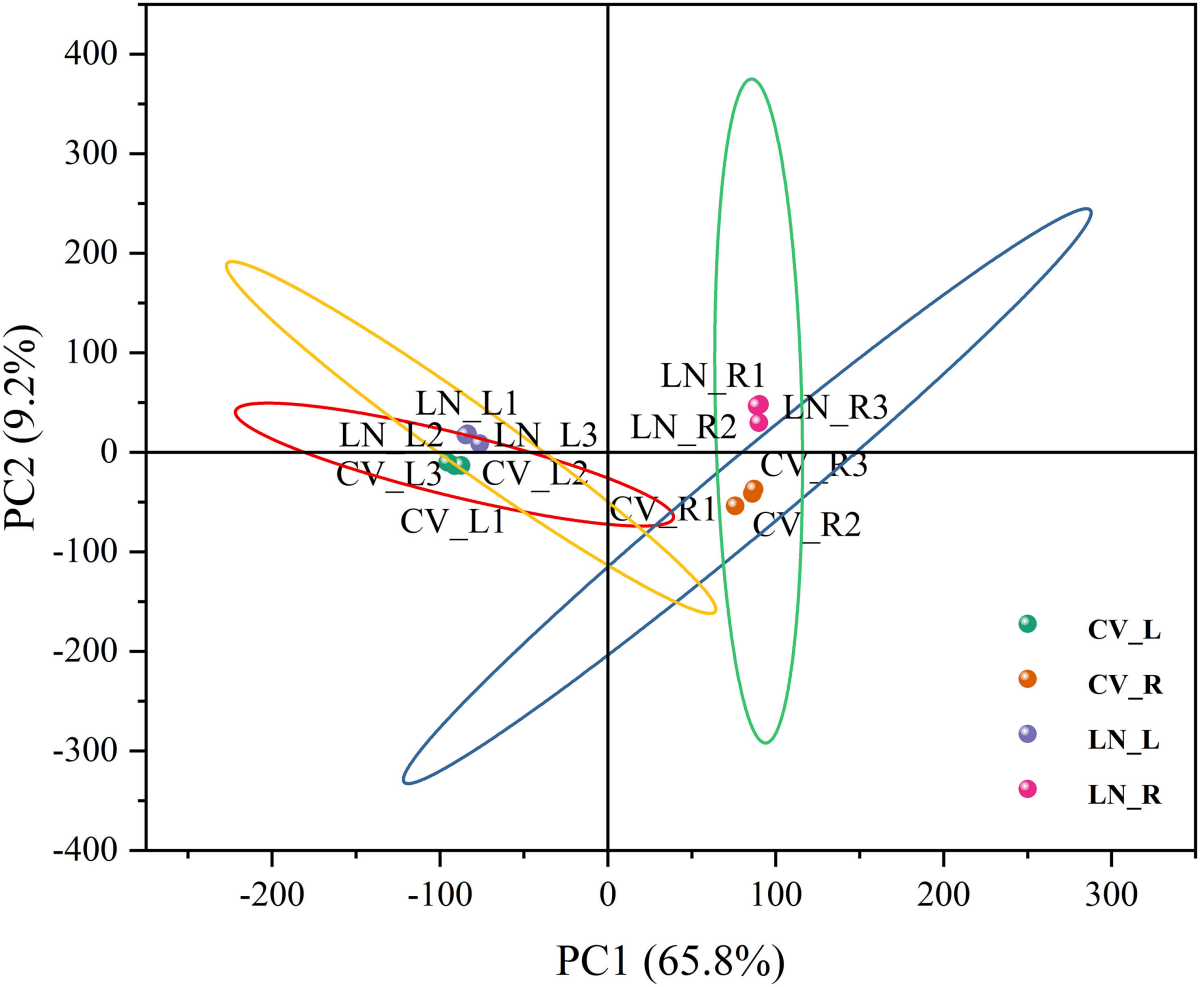
PCA plot of transcriptomes under low N stress in sugar beet. CV_R, control group, root; CV_L, control group, leaf; LN_R, low N group, root; LN_L, low N group, leaf; PCA, principal component analysis. The same abbreviations are used below.

To clarify the regulatory profile of genes related to nitrogen metabolism in sugar beet seedlings under LN stress, the transcriptional changes in the LN-tolerant germplasm (780016B/12 superior) were investigated by RNA sequencing after 0.5 mmol/L N treatment for 12 h. A total of 1,130,329,346 raw reads and 1,088,994,086 clean reads (**Supplementary Table 2**) were obtained. The GC content ranged from 41.08% to 43.15%, and the Q20 and Q30 values were >97.01% and >92.66%, respectively. Compared with the sugar beet reference genome (RefBeet-1.2.2), the alignment rate within each treatment group was > 98%. Therefore, the depth of this sequencing was sufficient. Principal component analysis (PCA) of the transcriptome showed a high degree of similarity between the three biological replicates within each treatment group. A clear separation of CV_R and LN_R data was observed, while a closer distance between CV_L and LN_L was detected (**Figure 1**). This indicated that short-term N deficiency influenced more transcription products in roots than in leaves.

### 3.2 Differentially expressed genes in leaves and roots of sugar beet seedlings under LN

**Figure 2:**
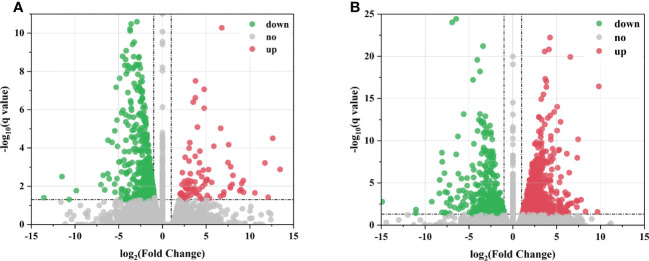
Differentially expressed genes (DEGs) of sugar beet under LN treatment. **(A, B)** Volcano plot of DEGs in leaves and roots between the control and low N treatments, respectively. The x-axis represents changes in gene expression ploidy across LN stress; the y-axis represents statistically significant differences in gene expression. Scattered dots represent individual genes; gray dots indicate no significant differences in transcripts, red dots indicate significant differences in downregulated genes, and green dots indicate significant differences in upregulated genes.

The transcript levels of the DEGs were measured using FPKM as a metric, which takes into account the effects of sequencing depth and gene length on fragment count. LN-responsive genes were further screened (log_2_^|FC|^ ≥ 1; q value < 0.05). A total of 580 DEGs were identified in leaves, of which 91 were upregulated and 489 were downregulated (**Figure 2A**). A total of 1,075 DEGs were found in roots, of which 689 were upregulated and 386 were downregulated (**Figure 2B**). The number of DEGs in roots was greater and more significant than those in leaves, which might be because roots were the first to respond to stress stimuli.

**Figure 3:**
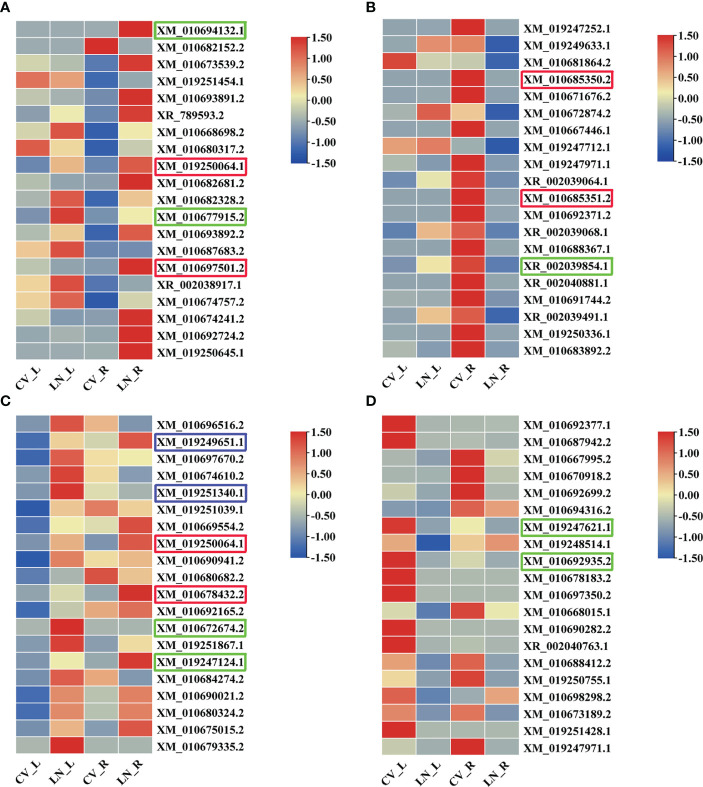
Heatmap of the top 20 significantly upregulated (**A**, roots; **C**, leaves) and downregulated (**B**, roots; **D**, leaves) DEGs responsive to LN in sugar beet. The redder the color, the more pronounced the expression of that transcript. DEGs, differentially expressed genes.

In the top 20 significantly upregulated (log_2_^FC^ ≥ 1; q value < 0.05) and downregulated (log_2_^FC^ ≤ −1; q value < 0.05) DEGs (**Figure 3**, **Supplementary Table 3**), the differential expression fold changes were all above 5; in roots, between 6.115 and 9.814 were upregulated and between −14.907 and −7.112 were downregulated; and in leaves, between 6.792 and 13.464 were upregulated and between −13.562 and −5.424 were downregulated. Among them, vacuolar sorting protein 39 (*VPS39*, XM_019250064.1) and cytochrome P450 (*CYP76AD1*, XM_010697501.2/CYP72A397, and XM_010685350.2/XM_010685351.2/XM_010678432.2) were transcribed at high levels in both leaves and roots of sugar beet and might play important roles in LN tolerance in sugar beet.

The transcription factors BIM2 (*BIM2*, XM_019249651.1) and bHLH67 (*BHLH67*, XM_019251340.1) were found to be upregulated in leaves (log_2_^FC^ = 12.620 and log_2_^FC^ = 10.580). In addition, 6,7,8-trihydroxycoumarin synthase (*CYP71AZ3*, XM_010672674.2) and arginine—tRNA ligase, chloroplastic/mitochondrial (*EMB1027*, XM_010680324.2) were also upregulated in leaves (log_2_^FC^ = 7.814 and log_2_^FC^ = 7.028); quinolinate synthase, chloroplastic (*QS*, XM_019247621.1) and gibberellin 3-beta-dioxygenase 1 (*GA3OX1*, XM_010692935.2) were downregulated in leaves (log_2_^FC^ = −6.023 and log_2_^FC^ = −6.224). In roots, NRT1/PTR FAMILY 7.3-like (XM_010694132.1) was upregulated (log_2_^FC^ = 9.815); DEGs encoding amino acids were also transcribed in roots: primary amine oxidase (*AMO*, XM_010677915.2) and aspartyl protease family protein 1 (*APF1*, XR_002039854.1) were upregulated (log_2_^FC^ = 6.701) and downregulated (log_2_^FC^ = −8.342), respectively.

### 3.3 The transcript pattern, biological processes, and pathways of DEGs

**Figure 4:**
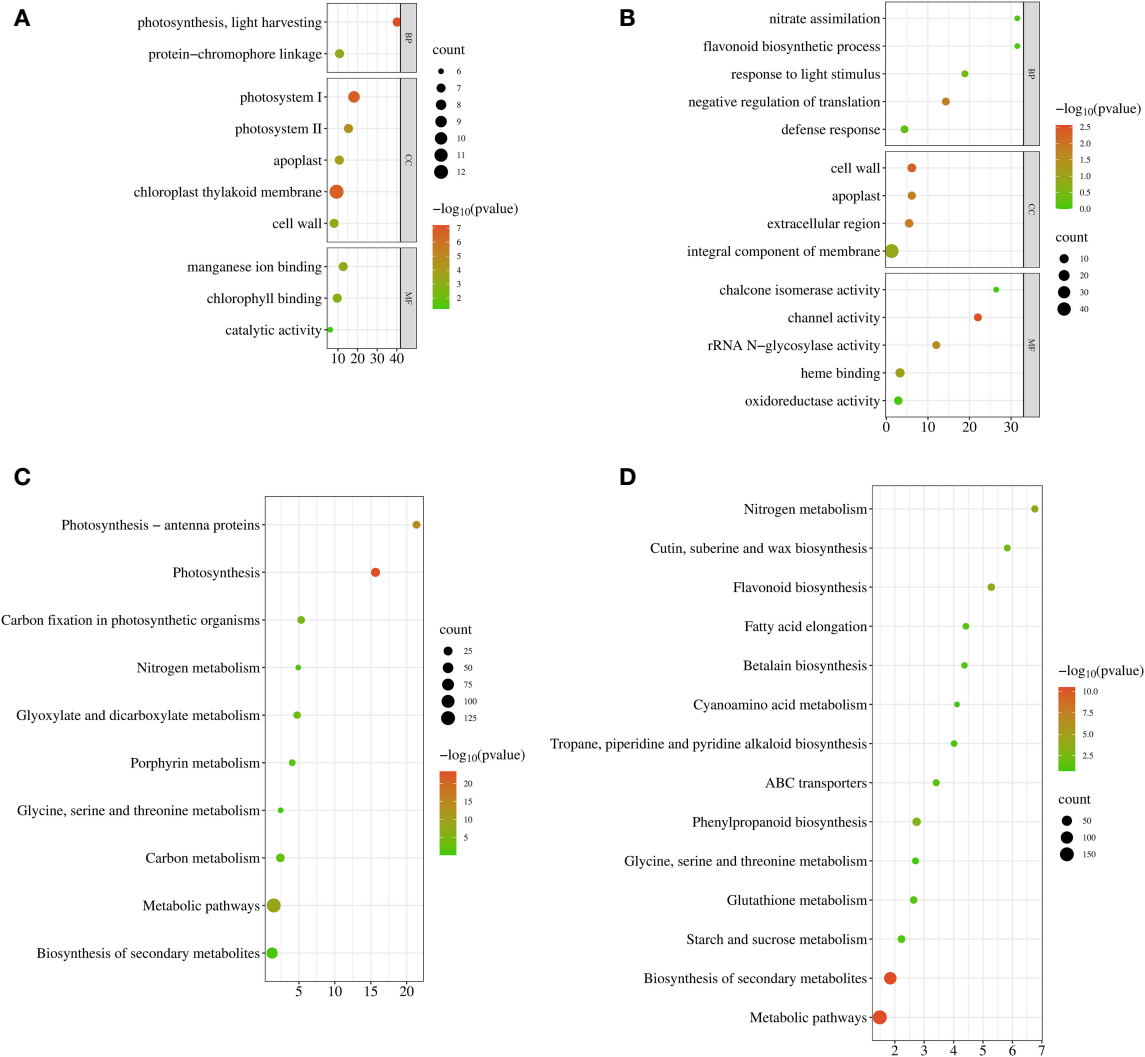
Functional enrichment of DEGs of sugar beet under low N stress. **(A, B)** Scatter plot of GO functional enrichment in leaves and roots. **(C, D)** Scatter plot of KEGG enrichment in leaves and roots. The x-axis is the enrichment factor; the y-axis is the enrichment of entries. The size of the bubbles represents the number of DEGs on the annotated items; the color represents the enrichment degree −log_10_^(p-value)^. DEGs, differentially expressed genes; GO, Gene Ontology; KEGG, Kyoto Encyclopedia of Genes and Genomes.

GO enrichment analysis of DEGs was performed using GOSeq, topGO, and hmmscan (Release 2.12) (Young et al., 2010). GO terms with FDRs less than 0.05 were considered significantly enriched for DEGs. In leaves, a total of 165 DEGs (log_2_^|FC|^ ≥ 1, q value < 0.05) were significantly enriched in two biological process (BP), five cellular component (CC), and two molecular function (MF) categories (**Figure 4A**). They were “photosynthesis, light harvesting (GO:0009765)” and “protein-chromophore linkage (GO:0018298)” in BP. The top three of the CC category were “photosystem I (GO:0009522)”, “chloroplast thylakoid membrane (GO:0009535)”, and “photosystem II (GO:0009523)”. In the MF category, “manganese ion binding (GO:0030145)” and “chlorophyll binding (GO:0016168)” were significantly enriched. A total of 526 DEGs (log_2_^|FC|^ ≥ 1, q value < 0.05) in the roots were obtained in one BP, three CC, and two MF categories (**Figure 4B**), and they were mainly related to “negative regulation of translation (GO:0017148)”, “cell wall (GO:0005618)”, “extracellular region (GO:0005576)”, “apoplast (GO:0048046)”, “channel activity (GO:0015267)”, and “rRNA N-glycosylase activity (GO:0030598)”. These results suggested that the DEGs involved in low nitrogen stress were mainly related to photosynthesis, transport, and stimulus response to stress.

In organisms, genes usually perform their biological functions in coordination with each other. The important biochemical metabolic and signal transduction pathways in which specific genes are involved (Kanehisa et al., 2008) can be identified by KEGG enrichment analysis. FDR ≤ 0.05 was defined as pathways significantly enriched in DEGs, and we used KOBAS (v2.0) (Mao et al., 2005) for pathway enrichment analysis. Seven and nine pathways were significantly enriched in LN-treated leaves and roots, respectively (**Figures 4C, D**). The enriched pathways were “photosynthesis (bvg00195)”, “photosynthesis-antenna proteins (bvg00196)”, “metabolic pathways (bvg01100)”, “carbon fixation in photosynthetic organisms (bvg00710)”, “glyoxylate and dicarboxylate metabolism (bvg00630)”, “carbon metabolism (bvg01200)”, and “porphyrin metabolism (bvg00860)” in leaves and “biosynthesis of secondary metabolites (bvg01110)”, “metabolic pathways (bvg01100)”, “flavonoid biosynthesis (bvg00941)”, “nitrogen metabolism (bvg00910)”, “phenylpropanoid biosynthesis (bvg00940)”, “cutin, suberine and wax biosynthesis (bvg00073)”, “fatty acid elongation (bvg00062)”, “ABC transporters (bvg02010)”, and “glutathione metabolism (bvg00480)” in roots. These results indicated that photosynthesis and synthesis of secondary metabolites were key pathways for sugar beet in response to LN.

### 3.4 Response of differentially expressed hub genes to LN stress

**Figure 5:**
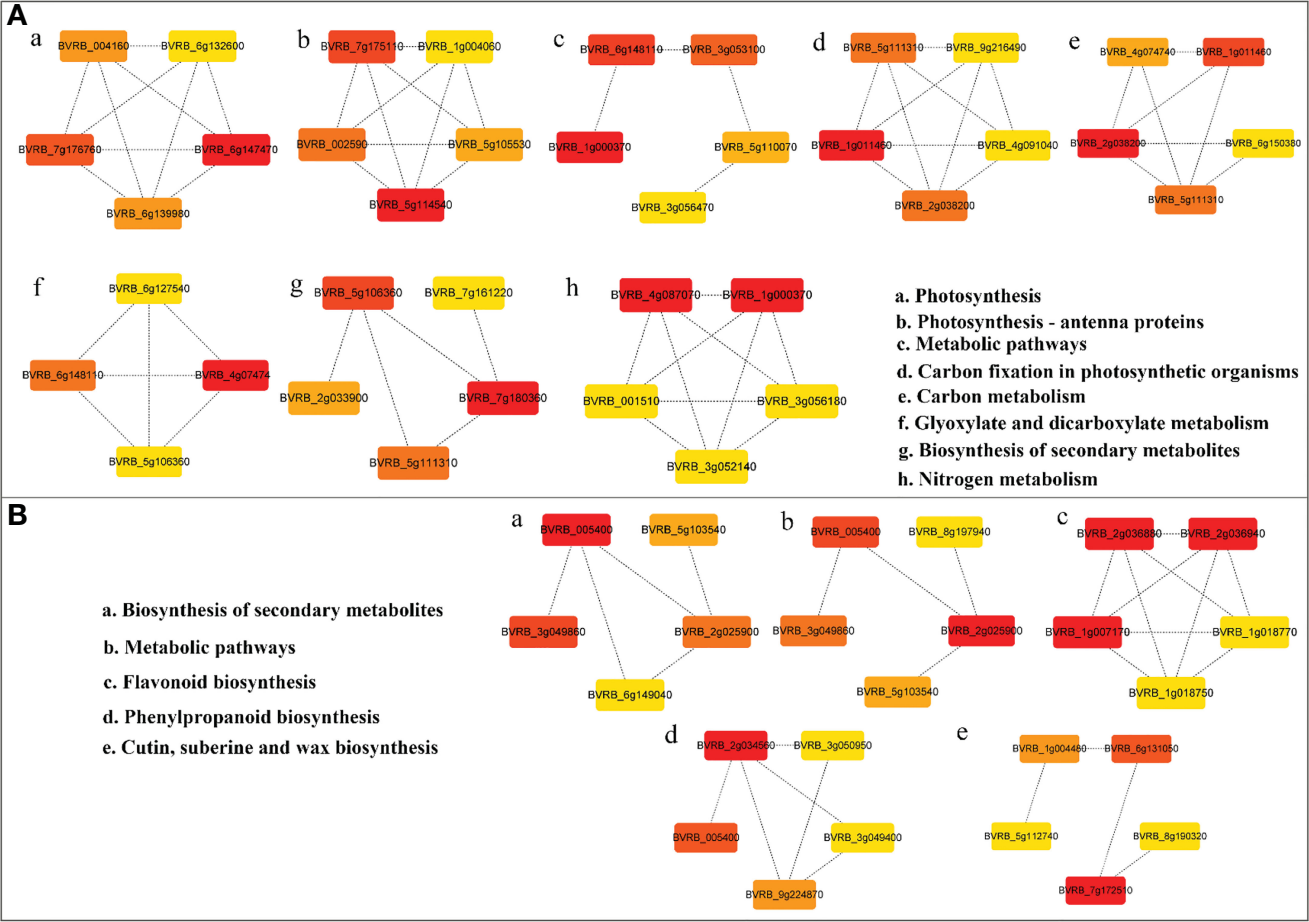
PPI network of primary and secondary metabolism of DEGs in sugar beet under LN stress. **(A, B)** PPI network diagram of downregulated and upregulated DEGs, respectively. Proteins are represented as nodes, and interactions are represented as edges. The color of the proteins indicates the strength of the protein interactions; the redder the color, the stronger the interactions. Only pathways with categories in error incidence rate (FDR) <0.01 were considered (**Supplementary Table 4**). PPI, protein−protein interaction; DEGs, differentially expressed genes; FDR, false discovery rate.

PPI networks were constructed using DEGs (log_2_^|FC|^ > 1, q value < 0.05) in LN-tolerant germplasm to explain the importance of these interactions in physiological biochemistry and signal transduction. Differential expression was screened for hub genes of each pathway using Cytoscape (**Figure 5**, **Supplementary Table 4**). Among the downregulated DEGs, the core genes associated with photosynthesis were photosystem II core complex proteins psbY, chloroplastic (*PsbY*, BVRB_6g147470) (**Figure 5Aa**), and chlorophyll *a*–*b* binding protein 4, chloroplastic (*CAB4/Lhca4*, BVRB_5g114540) (**Figure 5Ab**). The essential genes associated with carbon metabolism are fructose 1,6-bisphosphatase, cytosolic (*FBP*, BVRB_1g011460) (**Figure 5Ad**), and phosphoglycerate kinase, chloroplastic (*PGK*, BVRB_2g038200) (**Figure 5Ae**). Ferredoxin-nitrite reductase, chloroplastic (*NIR*, BVRB_4g087070), and nitrate reductase [NADH] (*NR*, BVRB_1g000370) (**Figure 5Ah**) are the core genes associated with nitrogen metabolism. Phosphoglycolate phosphatase 1A, chloroplastic (*PGLP1A*, BVRB_4g074740) (**Figure 5Af**), is highly associated with the glyoxylate and dicarboxylic acid metabolic pathways. Magnesium protoporphyrin IX methyltransferase, chloroplastic (*CHLM*, BVRB_7g180360) (**Figure 5Ag**), is a key gene for secondary metabolite biosynthesis. In conclusion, the downregulated DEGs were mainly closely related to photosynthesis, C/N metabolism, and secondary metabolite synthesis under low N stress in sugar beet.

For upregulated DEGs, the key genes in the secondary metabolic biosynthesis and metabolic pathways are likely cinnamyl alcohol dehydrogenase 6 (*CAD6*, BVRB_005400) (**Figure 5Ba**), and proline dehydrogenase 2, mitochondrial (*POX2*, BVRB_2g025900 (**Figure 5Bb**). Chalcone-flavanone isomerase (*CHI*, BVRB_2g036880), chalcone-flavanone isomerase (*CHI*, BVRB_2g036940), and dihydroflavonol 4-reductase (*DFRA*, BVRB_1g007170) (**Figure 5Bc**) play key roles in flavonoid biosynthesis. The key gene for phenylpropanoid biosynthesis is spermidine hydroxycinnamoyl transferase (*SHT*, BVRB_2g034560) (**Figure 5Bd**). Cytochrome P450 86A1 (*CYP86A1*, BVRB_7g172510) is vital for keratin, cork, and wax key genes in the biosynthetic pathway. It can be speculated that the upregulated DEGs involved in secondary metabolite synthesis and flavonoid and phenylpropanoid biosynthesis fulfill crucial functions in the sugar beet response to LN.

### 3.5 Correlation analysis between low nitrogen stress and the expression levels of key genes

**Figure 6:**
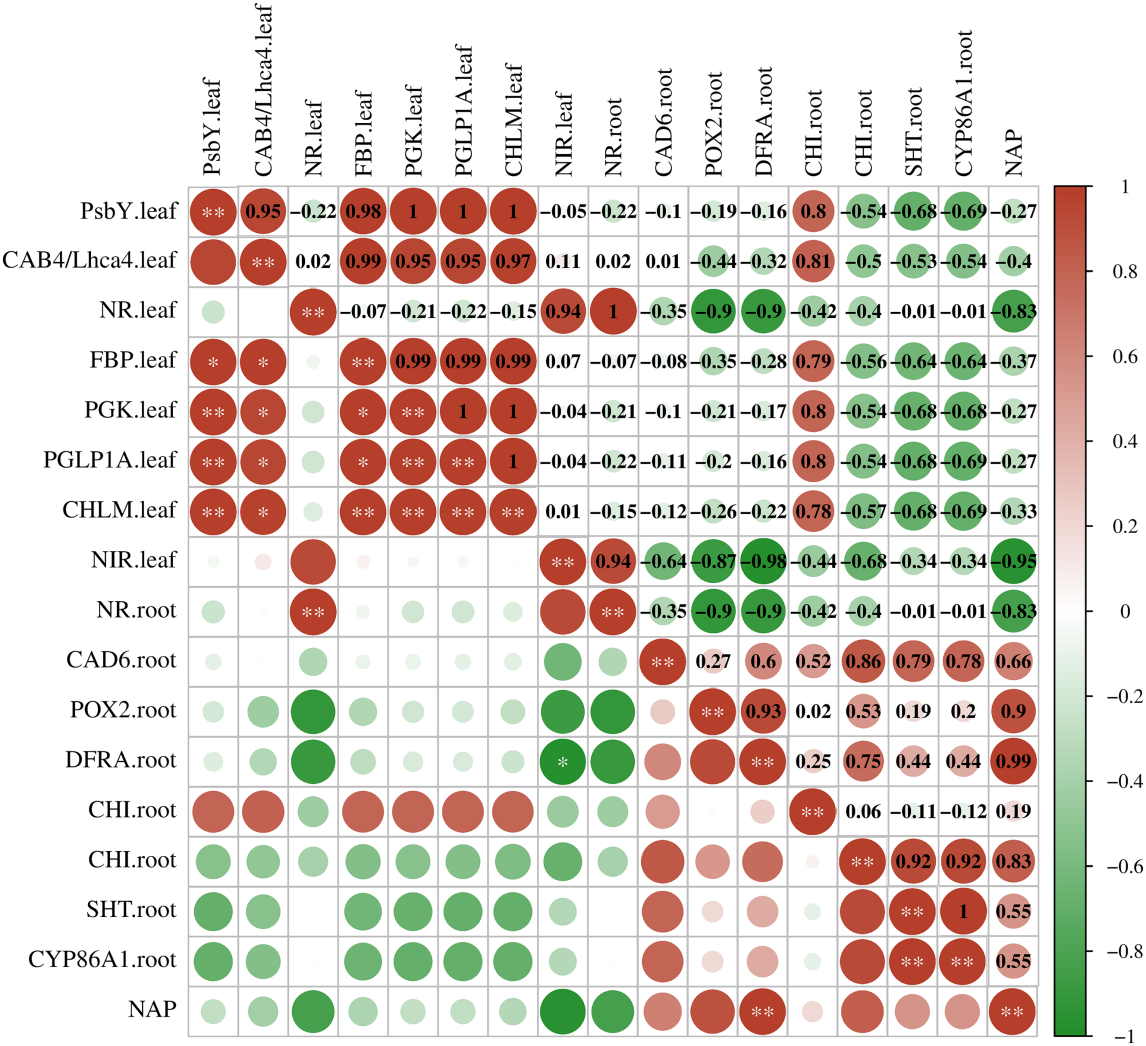
Pearson’s correlation coefficient (r) of the expression level of key genes. PSBY. leaf, photosystem II core complex proteins psbY, chloroplastic; CAB4/Lhca4. leaf, chlorophyll *a*–*b* binding protein 4; NR. leaf, nitrate reductase [NADH]; FBP. leaf, fructose 1,6-bisphosphatase, cytosolic; PGK. leaf, phosphoglycerate kinase, chloroplastic; PGLP1A. leaf, phosphoglycolate phosphatase 1A; CHLM. leaf, magnesium protoporphyrin IX methyltransferase; NIR. leaf, ferredoxin-nitrite reductase; CAD6. root, probable cinnamyl alcohol dehydrogenase 6; POX2. root, proline dehydrogenase 2, mitochondrial; DFRA. root, dihydroflavonol 4-reductase; CHI. root, chalcone-flavanone isomerase; SHT. root, spermidine hydroxycinnamoyl transferase; CYP86A1. root, cytochrome P450 86A1; NAP, nitrogen accumulation in plants. Scale: bright blue to bright red represents negative to positive correlations. *p < 0.05, **p < 0.01.

To understand the relationship between DEGs and LN stress, we analyzed the correlation between them using Pearson’s correlation analysis (**Figure 6**). Nitrogen accumulation in plants was positively correlated with *DFRA* in the roots (coefficient = 0.99, p < 0.01). *PsbY* and *CAB4* in the leaves were coregulated with four DEGs, *FBP*, *PGK*, *PGLP1A*, and *CHLM* (coefficient ≥ 0.95, p < 0.05), and the coefficients equal to 1 indicated a stronger correlation with each other. *PGK*, *GLP1A*, and *CHLM* had positive correlations with each other (p < 0.05). *DFRA* in the roots was negatively correlated with NIR in the leaves (coefficient = −0.98, p < 0.05). There may be remote regulation between flavonoid biosynthesis and nitrogen metabolism in sugar beet roots and leaves.

### 3.6 Abundant DEGs involved in primary metabolism under LN stress

**Figure 7:**
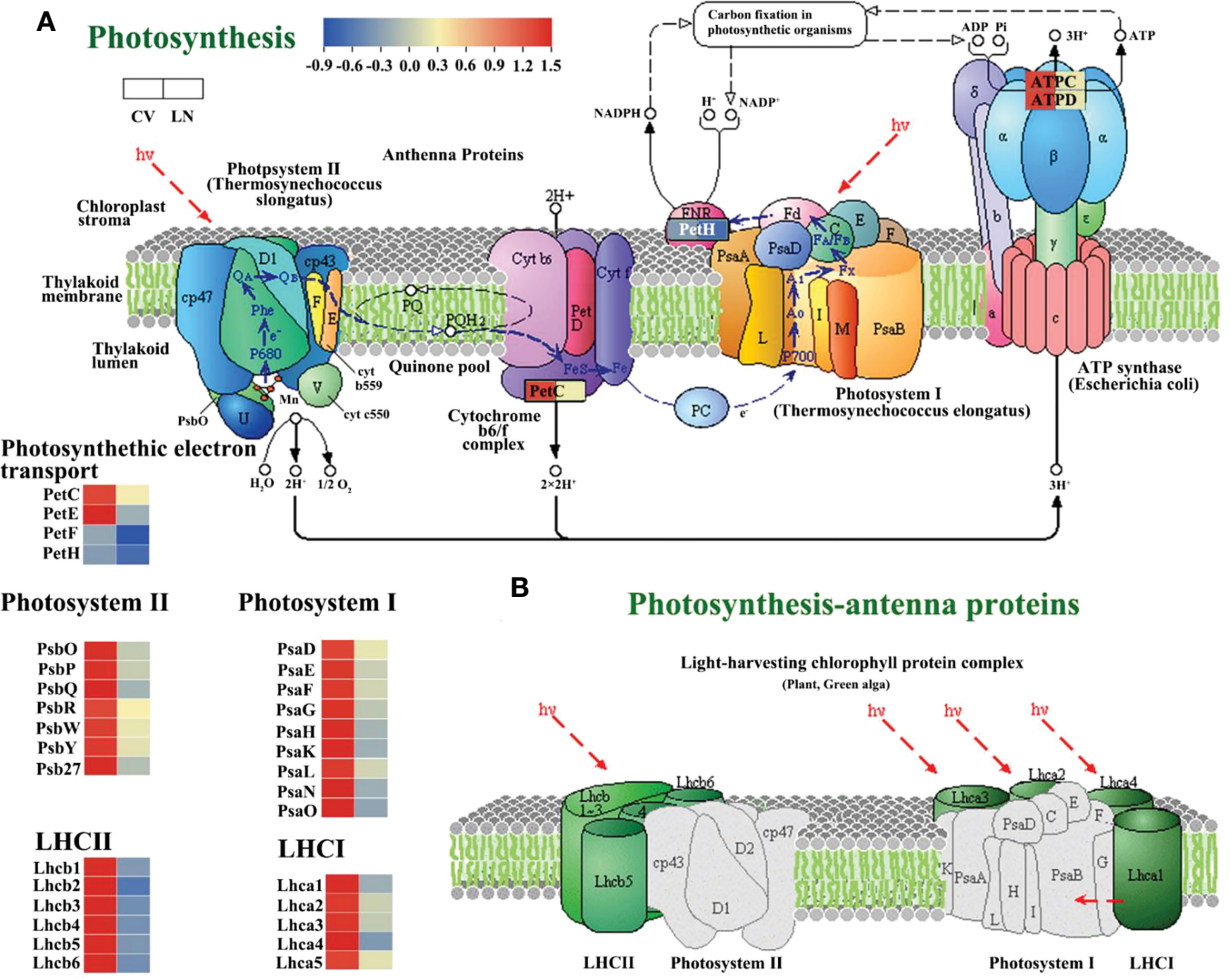
Significantly downregulated expression of photosynthesis-regulated genes under low N stress in sugar beet (**Supplementary Table 5**). **(A)** Photosynthetic pathway. **(B)** Photosynthetic antenna protein (Kanehisa et al., 2016; Kanehisa et al., 2017).

In the KEGG analysis, photosynthesis was found to be the most regulated pathway in the transcriptome. We annotated 48 genes involved in photosynthesis (**Supplementary Table 5**), and 15 of them were associated with photosynthesis - antenna protein. All of these genes were significantly downregulated in response to LN. This was similar to genes involved in photosynthesis in *Arabidopsis*, which were found to be downregulated at the mRNA level under nitrogen starvation conditions (Peng et al., 2007; Kiba et al., 2011). Under LN stress, DEGs involved in PSII (*PsbO*, *PsbP*, *PsbQ*, *PsbY*, and *Psb27-1*) were all significantly downregulated in sugar beet (**Figure 7A**, **Supplementary Table 5**, q value < 0.01). In LHC II, Lhcb1, Lhcb2, Lhcb3, Lhcb4, Lhcb5, and Lhcb6 involved in the photosynthetic antenna protein pathway were also significantly downregulated (**Figure 7B**, **Supplementary Table 5**, q value < 0.01). *PetE*, *PetF*, and *PetH* mediating electron transfer between PS II and PS I were found to be downregulated (**Figure 7A**, **Supplementary Table 5**, q value < 0.05). The other downregulated DEGs included photosystem I reaction center subunits (*PsaE*, *PsaF*, *PsaG*, *PsaH*, *PsaK*, *PsaL*, *PsaN*, and *PsaO*) (**Figure 7A**, **Supplementary Table 5**, q value < 0.01) and *Lhca1*, *Lhca 2*, *Lhca 3*, *Lhca 4*, and *Lhca 5*, which are involved in photosynthetic antenna proteins in LHC I (**Figure 7B**, **Supplementary Table 5**, q value < 0.01). These data suggested that the light-dependent function of photosynthesis was diminished in 780016B/12 superior leaves due to LN supply.

**Figure 8:**
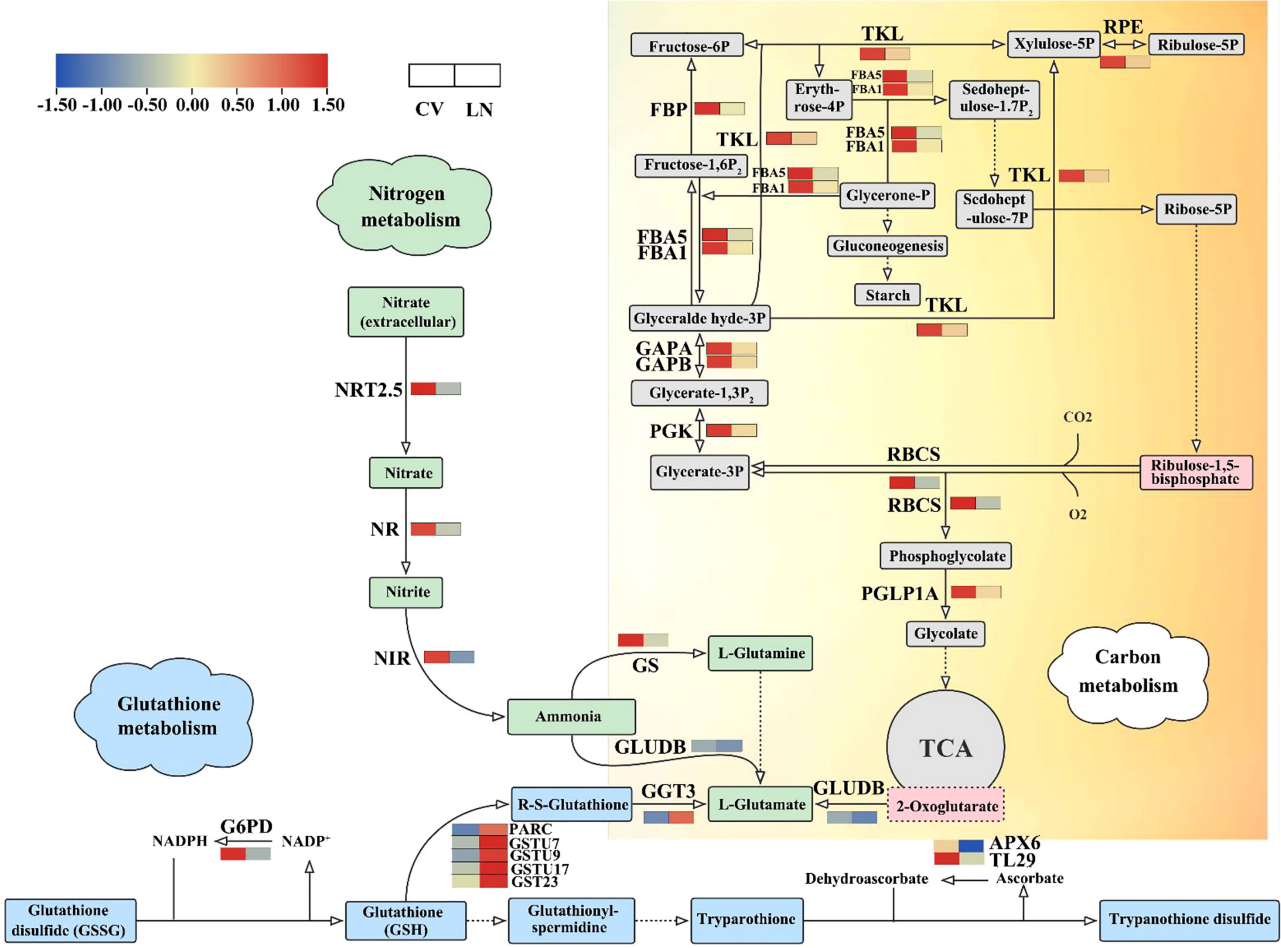
DEGs in C, N, and glutathione metabolism biosynthetic pathways under LN. The red to blue colors indicate upregulated to downregulated tendencies. Same as below. DEGs, differentially expressed genes.

In sugar beet, a large number of genes involved in carbon (C) metabolic pathways were significantly transcriptionally altered during nitrogen starvation. Of the 50 DEGs annotated (**Supplementary Table 6**), 15 are associated with carbon fixation in photosynthetic organisms, and 22 and 12 genes are within carbon metabolic and nitrogen metabolic pathways, respectively. Rubisco (ribulose-1,5-bisphosphate carboxylase/oxygenase) is an important carboxylase and an indispensable oxygenase in photorespiration in C_3_ plants. The transcript of the small subunit (*RBCS*) was downregulated under LN treatment (**Figure 8**, **Supplementary Table 6**), indicating that N deficiency influenced the N use efficiency for N fixation. C_3_ is the basic pathway for carbon assimilation, which can synthesize a variety of organic substances, such as sugars and starch. Starch can also be hydrolyzed in leaves to produce fructose and glucose, which need to be phosphorylated to enter glycolysis mediated by phosphofructokinase. Fructose 1,6-bisphosphatase (*FBP*), fructose-bisphosphate aldolase (*FBA5*, *FBA1*), phosphoglycerate kinase (*PGK*), and glyceraldehyde-3-phosphate dehydrogenase (*GAPA*, *GAPB*) were all downregulated under LN conditions (**Figure 8**, **Supplementary Table 6**). Decreases in phosphoglycerate lyase and phosphofructokinase are an important basis for the decrease in the C backbone of amino acid biosynthesis (Diop and Gallois, 2022; Fei et al., 2022). Sugar beet nitrogen-tolerant germplasms have less glucose entering the respiratory pathway, which may facilitate starch synthesis to maintain nocturnal leaf metabolism and support the production of other polysaccharides or glycoproteins (Meng et al., 2021).

During nitrogen starvation, genes directly involved in nitrogen metabolism were differentially transcribed. *NRT2.5, GS, GS1, NR, NIR, GLUDB* were significantly downregulated (**Figure 8**, **Supplementary Table 6**, q value < 0.05). This downregulation under LN was accompanied by extensive changes in cellular transport and was necessary for plant cells to maintain homeostatic growth (Cai et al., 2012). Glutathione (*GSH*) plays an important role in detoxification, antioxidant response, and redox homeostasis (Yamazaki et al., 2019). Under nitrogen deficiency, GST23 (*GST23*), glutathione S-transferase (*GSTU7*, *GSTU9*, and *GSTU17*), and l-ascorbate peroxidase 3 (*APX3*) were detected as significantly upregulated transcripts (**Figure 8**, **Supplementary Table 6**, q value < 0.01).

### 3.7 Essential DEGs in phenanthrene metabolism and flavonoid biosynthesis

**Figure 9:**
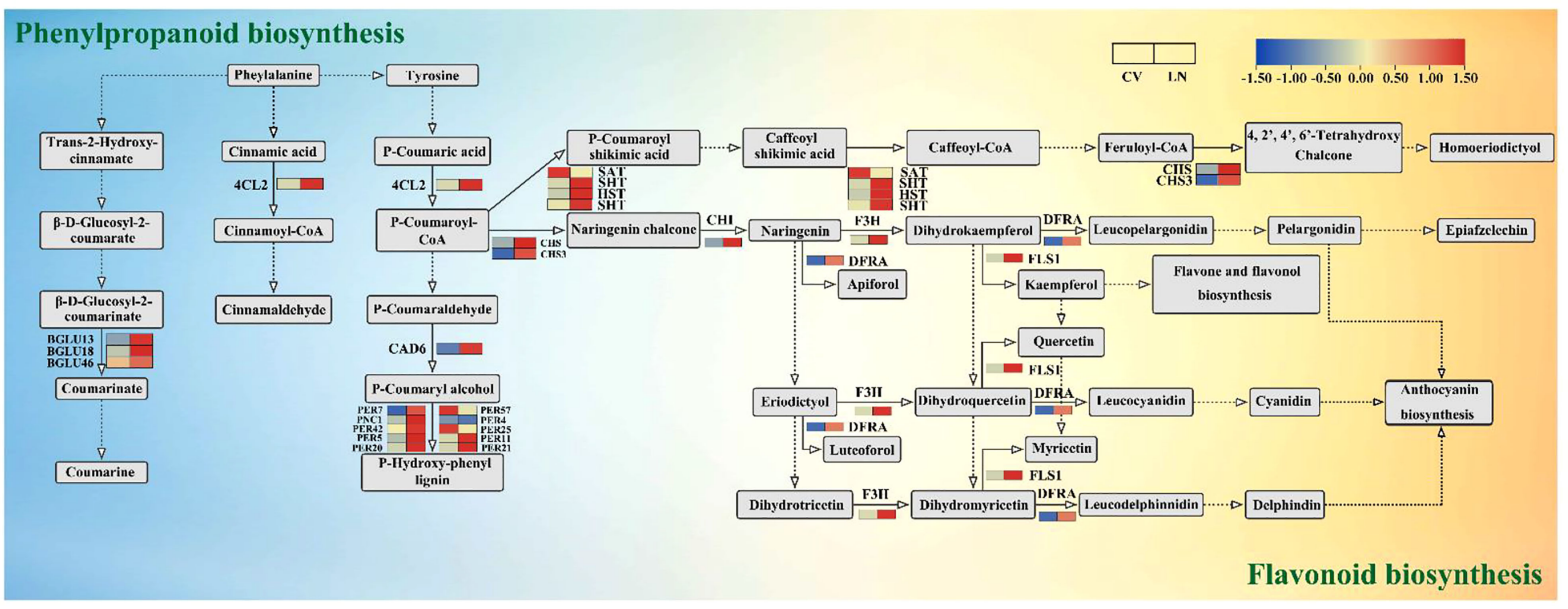
DEGs in the metabolic pathways of phenylpropanoid metabolism and flavonoid biosynthesis under LN. DEGs, differentially expressed genes.

In secondary metabolism, phenylpropane metabolism and flavonoid biosynthesis were found to be the most regulated pathways. In sugar beet, 30 and 13 DEGs were derived from phenylpropanoid metabolism and flavonoid biosynthesis, respectively (**Supplementary Table 7**). Sixteen peroxidase genes (*PER*, 13 upregulated and three downregulated) were transcriptionally altered due to nitrogen deficiency. The peroxidase multigene family encodes secreted glycoproteins involved in lignin biosynthesis, cell elongation, cell wall construction and differentiation, and pathogen defense responses (Passardi et al., 2004; Silva et al., 2022; Yang et al., 2022) (**Figure 9**, **Supplementary Table 7**). Members of the 4-coumarate-CoA ligase (*4CL*) family are key enzymes in lignin biosynthesis (Liang et al., 2021). In this study, *4CL2* was found to be upregulated for the synthesis of phenylpropanoid derivatives, such as lignin. *CAD6* was also upregulated, and it is required for the final step in stimulating lignin biosynthesis (Hirano et al., 2012). Thus, lignin formation and cell wall modification are regulated by morphological changes in the roots in response to nitrogen starvation signals (Liang et al., 2021). A total of five beta-glucosidase genes (*BGLU*, four upregulated and one downregulated) were observed in response to LN stress in this study. *BGLU13* is a defense protein that plays a key role in fighting against plant protection from salinity stress (Jia et al., 2019). β-Glucosidases are active in many metabolic processes, such as hydrolysis of derived oligosaccharides on the cell wall, as well as controlling the reactivity and biological activity of plant secondary metabolites and phytohormones, which regulate plant growth and development (Gan et al., 2022).

Flavonoids are derived from the phenylpropanoid metabolic pathway and are induced by C metabolism (Zhang et al., 2018b). In sugar beet, genes involved in flavonoid biosynthesis (*CHS*, *CHI*, *F3H*, *SHT*, *HST*, *DFRA*, and *FLS1*) were significantly upregulated under LN, but *SAT* was downregulated (**Figure 9**, **Supplementary Table 7**, q value < 0.05). Members of CHIs interact with chalcone synthase (*CHS*), flavonol synthase/flavanone 3-hydroxylase (*FLS*), naringin, and 2-oxoglutarate 3-dioxygenase (*F3H*) to form heterodimers, which may jointly play a regulatory role and participate in the flavonoid pathway (Wan et al., 2022).

### 3.8 Effect of low nitrogen stress on the photosynthetic characteristics of sugar beet seedlings

Furthermore, we detected the net photosynthetic rate (Pn), transpiration rate (Tr), stomatal conductivity (Gs), and intercellular CO_2_ concentration (Ci) of sugar beet under LN conditions. As shown in **Supplementary Figures 1A, C**, in sugar beet, both Pn and Gs showed significant decreases (p < 0.01) of 42.7% and 21.9%, respectively, under low-N stress conditions; Ci decreased significantly (p < 0.05) (**Supplementary Figure 1D**). *FBP* and *PGK* were significantly positively correlated with Ci and significantly negatively correlated (p < 0.001) with Pn (**Supplementary Figure 1E**). Tr and Gs exhibited significant negative correlations (p < 0.001) with *CHLM*, *PSBY*, and *PGLP1A*. Tr and Gs were negatively correlated with CHLM, *PSBY*, and *PGLP1A* (p < 0.001). This result suggested that the changes in photosynthetic gas exchange parameters under N deficiency might be determined by some photosynthetic DEGs in sugar beet.

### 3.9 Validation of RNA sequencing data by real-time fluorescence quantitative PCR

**Figure 10:**
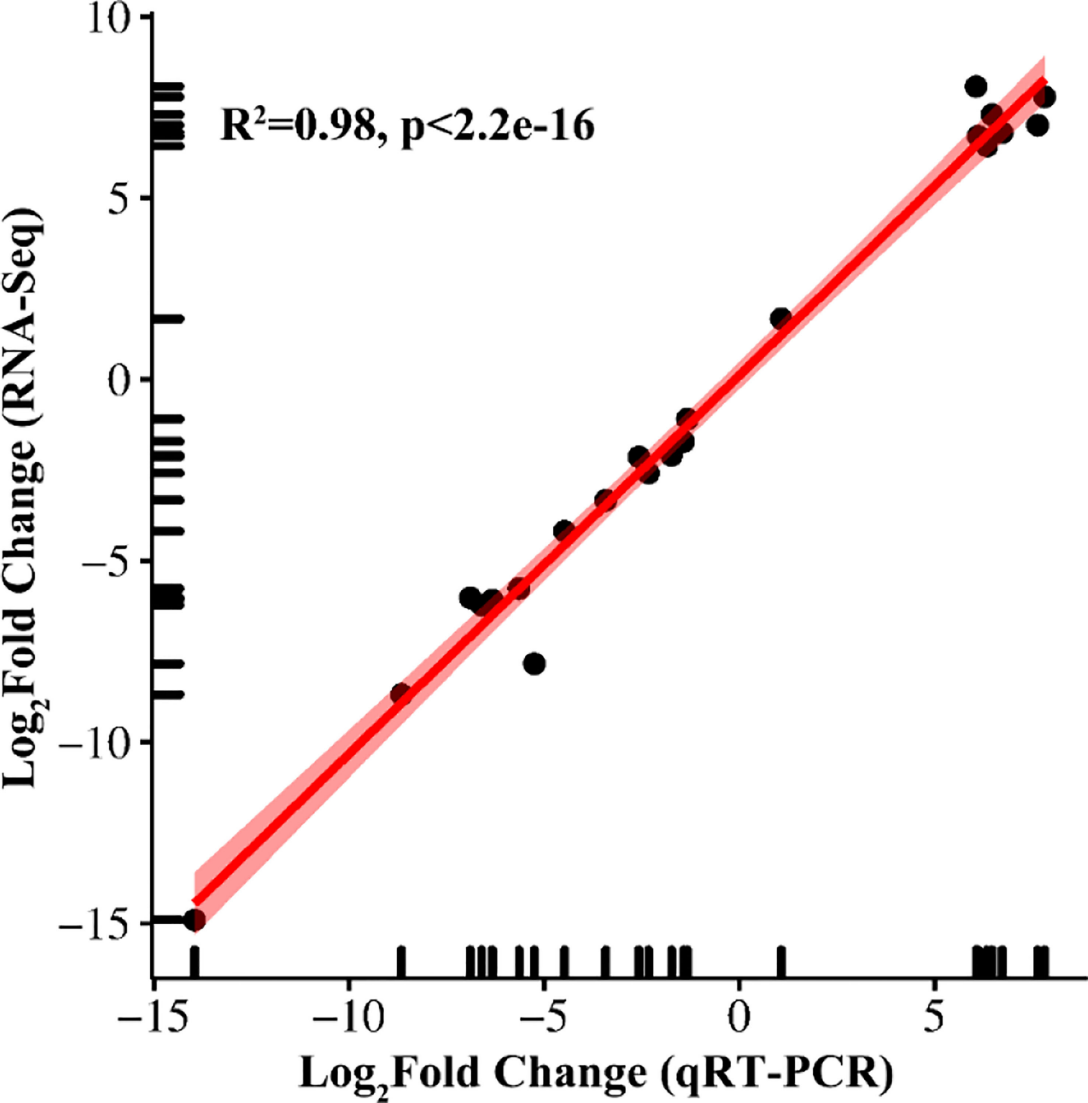
qRT−PCR validation of sugar beet RNA-seq results.

Gene fold changes between low nitrogen-stressed tissues and control treatments were confirmed using correlation analysis of qRT−PCR and RNA-seq data. A total of 22 genes were selected for qRT−PCR, including eight upregulated genes and 14 downregulated genes (**Figure 10**, **Supplementary Table 1**). There was a strong positive correlation coefficient (R^2^ = 0.98) for all these genes under low nitrogen stress, which confirmed the reliability and reproducibility of the RNA-seq data.

## 4 Discussion

### 4.1 The crucial role of downregulated DEGs in the photosynthesis of sugar beet suffering from N deficiency

Photosynthesis provides the carbon skeleton and energy for nitrogen assimilation and other metabolic processes (Meng et al., 2021). In the present study, LN stress caused a significant decrease in Pn, Ci, and Gs (**Supplementary Figure 1**, p < 0.05). It was shown that the reduction in photosynthetic capacity was mainly attributed to stomatal or non-stomatal limitation (Erel et al., 2015), which may depend on the changes in Pn, Ci, and Gs. In plants grown under nitrogen-deficient conditions, coordination between PSII activity and CO_2_ assimilation is important to maintain photosynthesis efficiency (Sun et al., 2022). In sugar beet, most of the DEGs involved in photosynthesis and photorespiration, including PSI, PSII, ATP synthase, and Rubisco, were downregulated (**Figure 7**, **Supplementary Table 5**). The reason for this may be that Rubisco breaks down under low nitrogen stress, plant photosynthetic activity is reduced, and ultimately plant growth may be inhibited (Curci et al., 2017). It was deduced that the downregulated *PetE*, *PetF*, and *PetH* in sugar beet under low nitrogen stress reduced the energy provided by photosynthetic biocarbon fixation due to the decreased Calvin cycle activity and NADPH consumption, resulting in reduced ATP (ATPC and ATPD downregulation) utilization (**Figure 7A**, q value < 0.01). Some of these genes have been proven to be of general importance for photosynthesis and plant growth, including PSII dissolved oxygen enhancing proteins, cytochrome b6/f complex proteins, PSI subunits, FNRs, and Rubisco (Ermakova et al., 2019; Meng et al., 2021), which is consistent with our hypothesis that the identified DEGs in sugar beet played key roles in nitrogen use efficiency (NUE) under low nitrogen conditions.

Many studies have reported that low nitrogen stress affects photosynthesis by affecting light and dark reactions in chloroplasts (Sultana et al., 2020; Tantray et al., 2022). Excited energy captured by the LHC is transferred to the core antenna complex and ultimately used in PSI and PSII reaction centers (RCs) for photochemical reactions. Both PSII and PSI in sugar beet have an antenna system (Pan et al., 2020) that includes a series of membrane proteins binding chlorophyll and carotenoids and belongs to the light-harvesting complex (LHC) superfamily, including LHCII and LHCI. Lhcb proteins are important components of the PSII-related light-harvesting complex (LHCII), which is composed of the minor antenna complex Lhcb4 (*CP29*), Lhcb5 (*CP26*), and Lhcb6 (*CP24*) and the major antenna complex containing homodimeric and heterodimeric trimers of the Lhcb1, Lhcb2, and Lhcb3 gene products (Boekema et al., 1995; Damkjær et al., 2009). In this study, all chlorophyll *a*–*b* binding proteins interacting with low nitrogen regulation were downregulated (**Supplementary Table 5**), which may be due to the decrease in chlorophyll content. Lhca encodes the LHCI protein and binds to the PSI core complex to form the PSI-LHCI supercomplex (BenShem et al., 2003; Ozawa et al., 2018). Consistent with the study of wheat varieties under nitrogen stress (Sultana et al., 2020), the expression of LHC components in PSII and PSI was significantly reduced in the current study (**Figure 7B**, **Supplementary Table 5**).

The identification of functional genes for improving low nitrogen tolerance is a feasible way to improve nitrogen use efficiency. In higher plants, *PsbO*, *PsbP*, and *PsbQ* in PSII have been identified as exogenous proteins required for optimal oxygen release (Bricker et al., 2012). PsbP proteins are important cofactors of PSII and are involved in key processes such as calcium binding and photosynthesis (Kakiuchi et al., 2012). In addition, PsbP proteins can act as signaling response factors in response to various adverse external environments (Liu et al., 2012; Ding et al., 2022). Inhibition of *PsbP* results in slower growth and some defects in PSII function, such as lower oxygen-evolving activity (oxygen-evolving), lower quantum yield, and slower electron transfer rate on the PSII donor side (Yi et al., 2007; Yi et al., 2009). Allahverdiyeva et al. found that *PPD1* is essential at the early stages of seedling development (Allahverdiyeva et al., 2013). In the present study, the PsbP structural domain-containing protein 1 (*PPD1*) gene, a member of the *PsbP* gene family, was significantly downregulated in sugar beet leaves under low nitrogen stress.

The core genes of the photosynthetic pathway are *PsbY* (**Figure 5Aa**) and *CAB4/Lhca4* (**Figure 5Ab**). Both *PsbY1* and *PsbY2* were identified in spinach (Gau et al., 1998). The *PsbY* protein of *Arabidopsis* photosystem II plays an important role in the redox regulation of cytochrome b559 (*PsbE* and *PsbF*) (Lotta et al., 2016). The *PsbY* gene was similarly found to be repressed in maize under low nitrogen stress (Du et al., 2021). According to **Supplementary Table 4**, two *PsbY* proteins are likely present in PSII of sugar beet, as in spinach. This hypothesis requires further confirmation.

Fe is a major element in the photosynthetic process and is involved in plant C metabolism (Quan et al., 2017). *PetF* is the major photosynthetic iron oxygen returning protein in chloroplasts and plays a role in electron transfer between PSI and FNR (Schmitter et al., 1988; Terauchi et al., 2009). Lin et al. found that overexpression of *PetF* increases the efficiency of chloroplasts in scavenging reactive oxygen species, thus conferring heat tolerance to plants (Lin et al., 2013). However, the results of the present study showed that *PetF* in sugar beet leaves was downregulated under low nitrogen stress. Its role is to reduce trivalent Fe(III) to divalent Fe(II) (Li et al., 2019), and thus, Fe may function in maintaining homeostasis in sugar beet under low nitrogen stress.

### 4.2 DEG performance on the balance of C and N metabolism in sugar beet under low N supply

The maintenance of coordinated carbon and nitrogen metabolism and the proper C/N balance is important for plant growth, development, and yield (Krapp and Traong, 2006). In sugar beet, as shown in the genes in **Figure 8** and **Supplementary Table 6**, carbon, nitrogen, and glutamate metabolism were found to play important roles in response to nitrogen deficiency, and the downregulation of glutamate dehydrogenase B (*GLUDB*) was a pivotal link between these processes, similar to the results of Li et al. (2018). Sequence alignment showed that BvGLUDB was highly homologous with GDHs in *Arabidopsis* (Fontaine et al., 2012). They share the same conserved structural domain of Glu/Leu/Phe/Val dehydrogenase, dimerization domain (InterPro: IPR006097) (**Supplementary Figure 2A**). In the *gdh1-2-3* mutation, the main physiological function of NADH-GDH is to provide 2-oxoglutarate for the tricarboxylic acid cycle by altering primary C and N metabolism (Fontaine et al., 2012). In the present study, BvGLUDB was found to be downregulated in leaves, which is speculated that the cycle of the metabolites Glu and 2-oxoglutarate is weakened. GDH controls the levels of C and N metabolites by changing the structure of heterohexamers (Tercé-Laforgue et al., 2013), and the related metabolic pathways play a major role in nitrogen assimilation (Tercé-Laforgue et al., 2015).

Rubisco and 2-oxoglutarate act as carbon and nitrogen starvation signals, respectively (Laurent et al., 2005). Carbon and nitrogen metabolism are fundamental metabolic processes in plants, and nitrogen assimilation requires carbon metabolism to provide energy and a carbon skeleton, which is mainly formed by α-ketoglutarate. α-Ketoglutarate changes significantly under low nitrogen treatment (Li et al., 2018), and it affects the conversion pathway to glutamate as a carbon source. Glutamate has been reported to exhibit considerable stability in amino acid profile changes, suggesting its homeostasis function in plants (Liao et al., 2022). The expression levels of nitrogen assimilation and amino acid transport genes play a decisive role in nitrogen utilization. In this study, we found that the *NRT2.5*, *NR*, *NIR*, *GS*, *GLUDB*, *GST*, and *GGT3* genes are involved in the regulation of homeostasis in sugar beet seedlings under low nitrogen application. They were significantly downregulated except for *GST* and *GGT3*, which were significantly upregulated. Reduced expression levels of *NRT2.5* lead to decreased high-affinity nitrate uptake (Lezhneva et al., 2014). *NR* and *NIR* are mainly responsible for the conversion of ${\text{NO}}_{3}^{-}$ to nitrite and nitrite to ${\text{NH}}_{4}^{+}$ , respectively (Rana et al., 2010), and *GLUDB* mediates the conversion of ${\text{NH}}_{4}^{+}$ to glutamate. *GSTU* and *GGT3* participate in glutathione (*GSH*) to *R*-*S*-glutathione to l-glutamate conversion. Glutamate is used as an amino donor for the synthesis of many other amino acids, such as proline, arginine, and lysine. This indicates that a low supply of nitrogen significantly promotes the glutathione metabolic biosynthetic pathway to quench reactive oxygen species (ROS) and protect cells from oxidative damage under short-term stress (Kumar and Trivedi, 2018).

Under stress conditions, different levels of regulation exist in cells to maintain a properly balanced metabolic ratio between carbon and nitrogen, which is necessary to avoid metabolic inefficiencies (Zhang et al., 2018a). In plants, photosynthesis is the energy source for carbon and nitrogen metabolism, and nitrogen deficiency has been reported to significantly reduce the photosynthetic capacity of plants (Tantray et al., 2022). Low nitrogen significantly attenuated the expression of genes controlling nitrogen metabolism (**Figure 8**, **Supplementary Table 6**). As a result, it competes with photosynthetic carbon sources for ATP and NADPH, increasing the photosynthetic electron transport burden while reducing the overall activity of PSII in sugar beet. Increasing nitrogen application can alleviate this competitive relationship and make it easier to balance carbon and nitrogen metabolism. In the present study, the transcript levels of ribulose bisphosphate carboxylase small subunit (*RBCS*) and phosphoglycolate phosphatase 1A (*PGLP1A*) were significantly downregulated (**Figure 8**), which may inhibit glycolate as a carbon backbone to synthesize other amino acids such as glutamate, interfering with the fixation or assimilation of photosynthetic carbon in sugar beet seedlings under low N conditions. When N is insufficient, plants can enhance the regulation of carbon metabolism in the roots. Phosphoglycerate kinase (*PGK*) catalyzes the first ATP production of the glycolytic pathway (Bowler, 2013). Glyceraldehyde-3-phosphate dehydrogenase (*GAPA*, *GAPB*), fructose-bisphosphate aldolase (*FBA1*, *FBA5*), and *PGK* were all downregulated, indicating that the intermediate steps of glycolysis were inhibited. *FBP* catalyzes the production of fructose-6P (*F6P*) from fructose-1,6P2 (*FDP*) in the gluconeogenesis pathway (Li et al., 2020). *FBP* was significantly downregulated under low nitrogen stress in sugar beet, indicating that the fructose phosphate pathway in glycolysis was inhibited. Therefore, the fructose content in sugar beet seedlings may be related to their tolerance to LN stress. Transketolase (*TKL*) was downregulated (**Figure 8**); it catalyzes reversible reactions in the carbon skeleton and plays a large role in carbon metabolism. We hypothesized that the conversion of the carbon skeleton is disturbed by nitrogen deficiency. Therefore, nitrogen deficiency not only leads to a decrease in nitrogen and carbon metabolism but also inhibits the synthesis of most amino acids to some extent (Hao et al., 2021). *DFRA* in the roots was found to be negatively correlated with *NIR* in the leaves (coefficient = −0.98, p < 0.05). Regulation of carbon and nitrogen balance can promote uptake, assimilation, and metabolic efficiency of carbon- and nitrogen-containing nutrients, which is a guide to improving low nitrogen tolerance in crops.

### 4.3 DEGs involved in conservative secondary metabolic regulatory networks in sugar beet in response to nitrogen starvation

In terms of energy and substrate availability, C status is considered to be a key factor in the accumulation of secondary metabolites (Beshir et al., 2019). Phenylpropanoids are an important metabolic pathway in which a large number of phenylalanine or tyrosine secondary metabolites, such as flavonoids and isoflavonoids, are produced. These important molecules are involved in many biological processes in plants (Betti et al., 2014). The phenylpropanoid metabolic pathway is closely related to lignin synthesis (Cong et al., 2013). Lignin is a major component of the plant skeleton and functions in plant root growth (Guo et al., 2001). Peroxidase superfamily protein (*PER*) is one of the key proteins involved in lignin synthesis and is able to catalyze lignin monomer precursors to produce *p*-hydroxyphenyl lignin (**Figure 9**). In the present study, 4-coumarate-CoA ligase 2 (*4CL2*) and probable cinnamyl alcohol dehydrogenase 6 (*CAD6*) were significantly upregulated and were speculated to be the major enzymes involved in lignin synthesis under low nitrogen application in sugar beet. Among the genes encoding peroxidase in this pathway, DEGs (*PER4*, *PER5*, *PER7*, *PER11*, *PER20*, *PER21*, and *PER42*) were upregulated compared to the downregulated *PER25* and *PER57* genes (**Figure 9**, **Supplementary Table 7**). This is different from previous results in rice (Guo et al., 2001) and oilseed rape (Qin et al., 2019). The reason is speculated to be that the reduction in *PER25* and *PER57* transcripts further triggered the response of sugar beet to a low nitrogen environment, but LN stress seems to promote lignin biosynthesis, which could contribute to the response of plants to various abiotic stresses (Dong and Lin, 2021).

The expression of beta-glucosidases (*BGLU13*, *BGLU18*, and *BGLU46*) involved in phenylalanine biosynthesis was upregulated under low nitrogen stress, and *BGLU13* catalyzed the conversion of β-d-glucosyl-2-coumarate to β-d-glucosyl-2-coumarinate and then to coumarin (**Figure 9**, **Supplementary Table 7**). Recent studies have shown that *BGLU13* is involved in glucose synthesis under saline stress (Jia et al., 2019). Han et al. (2020) found that BGLU18 in *Arabidopsis* is abundant in the petiole and localized mainly in the endoplasmic reticulum (ER), suggesting its involvement in defense against biotic stresses in the petiole. *BGLU46* is mainly located in the primary xylem (Baiya et al., 2018) and functions in the lignification process with *4CL2* (**Figure 9**). *Os4BGlu18* was found to be closely associated with *Arabidopsis BGLU46* in the rice genome (Opassiri et al., 2006). Further detailed examination of the distribution of *BGLU* needs to be verified in aboveground tissues of sugar beet (mainly leaves and petioles). Thus, *BGLUs* may be defense proteins in response to stress, and their upregulation may protect sugar beet from low nitrogen stress. The upregulated *POX2* and *APX* (**Supplementary Tables 6, 7**) can alleviate low nitrogen stress and improve LN tolerance, which is consistent with previous findings (Hao et al., 2021; Zhang et al., 2023). These results confirm that oxidative damage and compositional changes are sensitive responses for sugar beet to cope with low nitrogen stress.

Although a number of studies have investigated the effect of C or N on flavonoid biosynthesis, the mechanism by which C/N interactions affect flavonoid metabolism remains unclear (Klem et al., 2022). Chalcone synthase (CHS) is involved in the biosynthesis of precursor molecules for flavonoids and isoflavones (García-Calderón et al., 2020), generating naringenin chalcone with *p*-coumaroyl-CoA. Naringenin can also be catalyzed by chalcone isomerase (CHI) as a precursor of other flavonoids. The present study found that *CHS*, *F3H*, and *p*-coumaroyl-CoA were used as precursors of other flavonoids in sugar beet (**Figure 9**). *CHS*, *F3H*, *DFRA*, *FLS*, and *CHI* interacted with each other and played important roles in flavonoid biosynthesis (**Figures 5Bc**, **9**). *CHI* in the roots was closely associated with *PsbY*, *CAB4*, *PGK*, and *PGLP1A* involved in the photosynthetic carbon pathway (coefficient ≥ 0.8, p < 0.05, **Figure 6**), suggesting that dynamic changes in beet carbon metabolism directly affect the expression of genes related to photosynthesis (Sweetlove and Fernie, 2013; García-Calderón et al., 2020). *CHS* provides the basic carbon shelf structure for flavonoids and is a key step in the synthesis of flavonols, flavanones, and other substances. Upregulation of *CHS* and *CHI* facilitates the accumulation of naringenin and high-sage phenols (**Figure 9**) (Chen et al., 2021).

Flavonoids act as antioxidants to reduce oxidative damage caused by ROS accumulation due to abiotic stresses (Nakabayashi and Saito, 2015; Jia et al., 2019; Jiao et al., 2020). 2-Oxoglutarate 3-dioxygenase (*F3H*) has been shown to be a key gene for cold stress tolerance in tomatoes (Hu et al., 2019). F3H catalyzed the oxidation of the 3′ position of naringenin to produce dihydrokaempferol and catalyzed eriodictyol to dihydrotricetin and subsequently dihydromyricetin (**Figure 9**). They were then catalyzed by dihydroflavonol 4-reductase (*DFRA*) to produce leucocyanidin, anthocyanin, and leucodelphinnidin; flavonol synthase/flavanone 3-hydroxylase (*FLS1*) was used to produce kaempferol, quercetin, and myricetin (**Figure 9**). The upregulated flavonoid biosynthesis genes could increase the accumulation of flavonoids and thus reduce oxidative damage, thus improving the tolerance of sugar beet to low nitrogen stress. Amino acid sequence analysis revealed the presence of a common conserved structural domain in both sugar beet BvDFRA and AtDFR, the putative NADP binding site (**Supplementary Figure 2B**). It has been shown that the *AtDFR* gene ectopic expression determines the increase of anthocyanins and thus confers salt stress tolerance in *Brassica napus* L. (Kim et al., 2017). It is known that low nitrogen stress interferes with redox homeostasis in plant cells, which induces ROS production and leads to oxidative stress. DFRi transgenic plants by downregulation of *IbDFR* expression have reduced antioxidant capacity and are more sensitive to abiotic stress, implying an important biological role of flavonoids such as anthocyanins against oxidative stress *in vivo* (Wang et al., 2013).

## 5 Conclusion

**Figure 11:**
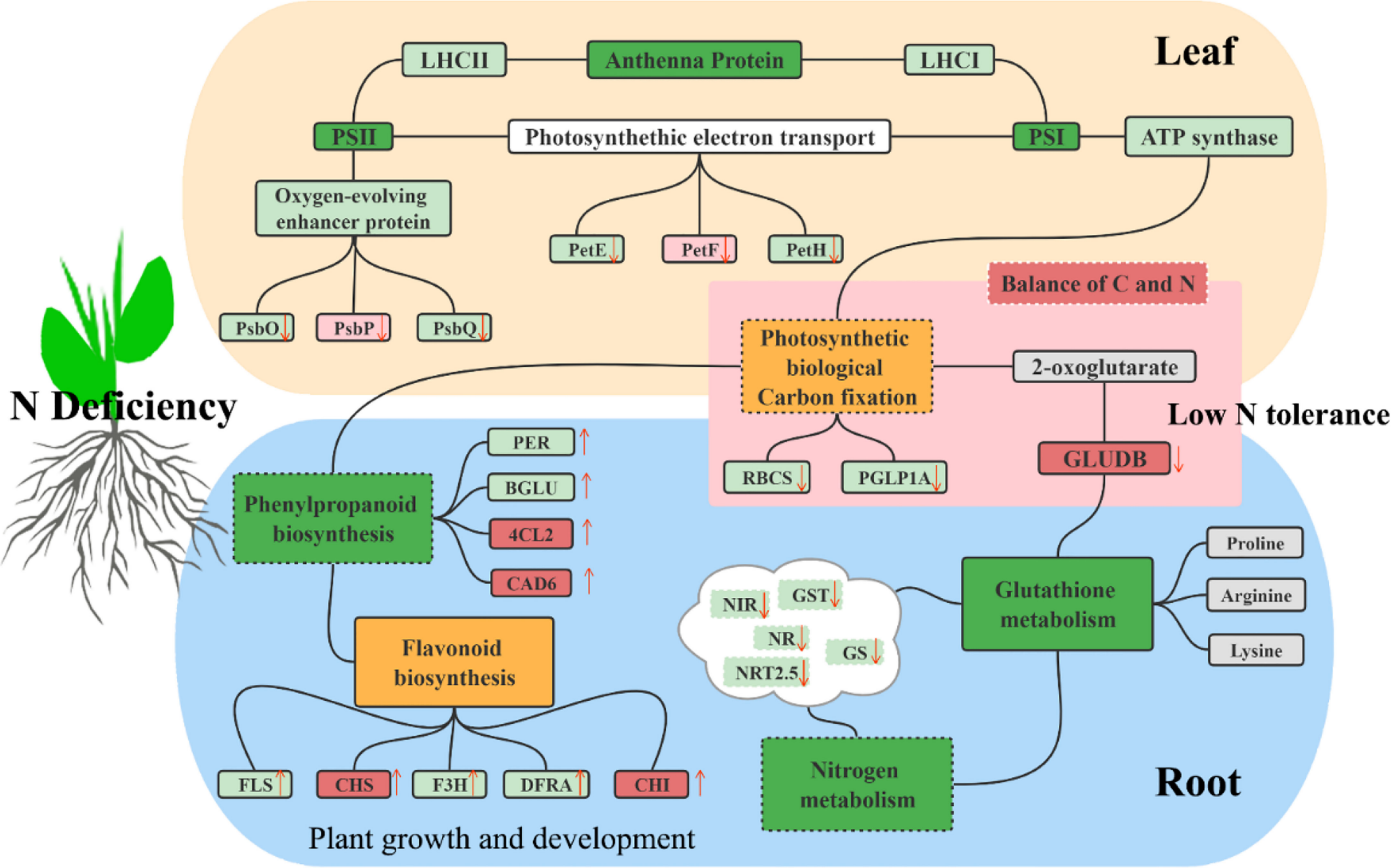
Modeling the response of sugar beet seedlings to low N stress.

The molecular tolerance and adaptation mechanisms of sugar beet to low nitrogen stress were identified by comprehensive transcriptome analysis. In summary, DEGs were extensively involved in the network of sugar beet primary and secondary metabolism (**Figure 11**). Low nitrogen stress suppressed the transcription of various genes involved in photosynthesis and C, N, and glutathione metabolism by regulating them in both leaves and roots. More C skeleton is used in secondary metabolism in sugar beet, which contributed to the increased expression of functional genes in phenylalanine and flavonoid biosynthesis. The present study contributed to the understanding of responsive relationships of gene networks related to the nitrogen metabolic pathway in sugar beet under low N conditions and provided a series of excellent genes for the improvement of sugar beet.

## Data availability statement

The datasets presented in this study can be found in online repositories. The names of the repository/repositories and accession number(s) can be found below: NCBI SRA database, accession number SRP422868.

## Author contributions

JL and DL conceived and designed the experiments, analyzed the data, wrote and reviewed the manuscript, and approved the final draft. WL and QY produced the graphs. XL provided reagents, materials, and analytical tools. JL, LX, and XY performed the experiments. Revisions were performed by QW, WT, and WX. All authors contributed to the article and approved the submitted version.

## References

[B1] Allahverdiyeva Y. Suorsa M. Rossi F. Pavesi A. Kater M. M. Antonacci A.. (2013). Arabidopsis plants lacking PsbQ and PsbR subunits of the oxygen-evolving complex show altered PSII super-complex organization and short-term adaptive mechanisms. Plant J. 75, 671–684. doi: 10.1111/tpj.12230 23647309

[B2] Baiya S. Mahong B. Lee S. Jeon J. Cairns J. R. K. (2018). Demonstration of monolignol β-glucosidase activity of rice Os4BGlu14, Os4BGlu16 and Os4BGlu18 in *Arabidopsis thaliana bglu45* mutant. Plant Physiol. Bioch. 127, 223–230. doi: 10.1016/j.plaphy.2018.03.026 29614441

[B3] Bao A. Zhao Z. Ding G. Shi L. Xu F. Cai H. (2015). The stable level of *glutamine synthetase 2* plays an important role in rice growth and in carbon-nitrogen metabolic balance. Int. J. Mol. Sci. 16, 12713–12736. doi: 10.3390/ijms160612713 26053400 PMC4490469

[B4] BenShem A. Frolow F. Nelson N. (2003). Crystal structure of plant photosystem I. Nature 426, 630–635. doi: 10.1038/nature02200 14668855

[B5] Beshir W. F. Tohge T. Watanabe M. Hertog M. L. Hoefgen R. Fernie A. R.. (2019). Non-aqueous fractionation revealed changing subcellular metabolite distribution during apple fruit development. Hortic. Res. 6, 98. doi: 10.1038/s41438-019-0178-7 31666959 PMC6804870

[B6] Betti M. García-Calderón M. Pérez-Delgado C. M. Credali A. Pal’ove-Balang P. Estivill G.. (2014). Reassimilation of ammonium in *Lotus japonicus* . J. Exp. Bot. 65, 5557–5566. doi: 10.1093/jxb/eru260 24948681

[B7] Bhatotia K. Khatodia S. Khurana S. (2015). Abiotic stress specific phosphorylation in plant proteins: a review. Curr. Adv. Agric. Sci. (An Int. Journal) 7, 95–100. doi: 10.5958/2394-4471.2015.00024.6

[B8] Boekema E. J. Hankamer B. Bald D. Kruip J. Nield J. Boonstra A. F.. (1995). Supramolecular structure of the photosystem II complex from green plants and cyanobacteria. Proc. Natl. Acad. Sci. U.S.A. 92, 175–179. doi: 10.1073/pnas.92.1.175 7816811 PMC42840

[B9] Bowler M. W. (2013). Conformational dynamics in phosphoglycerate kinase, an open and shut case? FEBS J. 587, 1878–1883. doi: 10.1016/j.febslet.2013.05.012 23684636

[B10] Bricker T. M. Roose J. L. Fagerlund R. D. Frankel L. K. Eaton-Rye J. J. (2012). The extrinsic proteins of photosystem II. Biochim. Biophys. Acta Bioenerg. 1817, 121–142. doi: 10.1016/j.bbabio.2011.07.006 21801710

[B11] Cai H. Lu Y. Xie W. Zhu T. Lian X. (2012). Transcriptome response to nitrogen starvation in rice. J. Biosci. 37, 731–747. doi: 10.1007/s12038-012-9242-2 22922198

[B12] Chen Y. Li Y. Yu L. Wang Y. Ni H. (2017). Analysis of the development of the sugar beet industry in china’s three main production areas. Sugar Crops China 39, 74–76 + 80. doi: 10.13570/j.cnki.scc.2017.04.024

[B13] Chen C. Zhou G. Chen J. Liu X. Lu X. Chen H.. (2021). Integrated metabolome and transcriptome analysis unveils novel pathway involved in the formation of yellow peel in cucumber. Int. J. Mol. Sci. 22, 1494. doi: 10.3390/ijms22031494 33540857 PMC7867363

[B14] Cong F. Diehl B. G. Hill J. L. Brown N. R. Tien M. (2013). Covalent bond formation between amino acids and lignin: cross-coupling between proteins and lignin. Phytochemistry 96, 449–456. doi: 10.1016/j.phytochem.2013.09.012 24099658

[B15] Cosgrove D. J. (2016). Plant cell wall extensibility: connecting plant cell growth with cell wall structure, mechanics, and the action of wall-modifying enzymes. J. Exp. Bot. 67, 463–476. doi: 10.1093/jxb/erv511 26608646

[B16] Curci P. L. Aiese Cigliano R. Zuluaga D. L. Janni M. Sanseverino W. Sonnante G. (2017). Transcriptomic response of durum wheat to nitrogen starvation. Sci. Rep. 7, 1–14. doi: 10.1038/s41598-017-01377-0 28446759 PMC5430780

[B17] Damkjær J. T. Kereïche S. Johnson M. P. Kovacs L. Kiss A. Z. Boekema E. J.. (2009). The photosystem II light-harvesting protein Lhcb3 affects the macrostructure of photosystem II and the rate of state transitions in arabidopsis. Plant Cell 21, 3245–3256. doi: 10.1105/tpc.108.064006 19880802 PMC2782274

[B18] Ding G. Cao L. Zhou J. Li Z. Lai Y. Liu K.. (2022). DNA Methylation correlates with the expression of drought-responsive genes and drought resistance in rice. Agronomy 12, 1445. doi: 10.3390/agronomy12061445

[B19] Diop M. Gallois J. (2022). Exploring new routes for genetic resistances to potyviruses: the case of the *Arabidopsis thaliana* phosphoglycerates kinases (PGK) metabolic enzymes. Viruses 14, 1245. doi: 10.3390/v14061245 35746717 PMC9228606

[B20] Dohm J. C. Minoche A. E. Holtgräwe D. Capella-Gutiérrez S. Zakrzewski F. Tafer H.. (2014). The genome of the recently domesticated crop plant sugar beet *(Beta vulgaris*). Nature 505, 546–549. doi: 10.1038/nature12817 24352233

[B21] Dong N. Q. Lin H. X. (2021). Contribution of phenylpropanoid metabolism to plant development and plant–environment interactions. J. Integr. Plant Biol. 63, 180–209. doi: 10.1111/jipb.13054 33325112

[B22] Du Q. Juan Y. Sadiq S. Yang R. Yu J. Li W. (2021). Comparative transcriptome analysis of different nitrogen responses in low-nitrogen sensitive and tolerant maize genotypes. J. Integr. Agric. 20, 2043–2055. doi: 10.1016/S2095-3119(20)63220-8

[B23] Erel R. Yermiyahu U. Ben-Gal A. Dag A. Shapira O. Schwartz A. (2015). Modification of non-stomatal limitation and photoprotection due to K and Na nutrition of olive trees. J. Plant Physiol. 177, 1–10. doi: 10.1016/j.jplph.2015.01.005 25659331

[B24] Ermakova M. LopezCalcagno P. E. Raines C. A. Furbank R. T. von Caemmerer S. (2019). Overexpression of the rieske FeS protein of the cytochrome b6f complex increases C_4_ photosynthesis in setaria viridis. Commun. Biol. 2, 1–12. doi: 10.1038/s42003-019-0561-9 31453378 PMC6697696

[B25] Fei X. Hu H. Luo Y. Shi Q. Wei A. (2022). Widely targeted metabolomic profiling combined with transcriptome analysis provides new insights into amino acid biosynthesis in green and red pepper fruits. Food Res. Int. 160, 111718. doi: 10.1016/j.foodres.2022.111718 36076459

[B26] Fontaine J.-X. Tercé-Laforgue T. Armengaud P. Clément G. Renou J.-P. Pelletier S.. (2012). Characterization of a NADH-dependent glutamate dehydrogenase mutant of arabidopsis demonstrates the key role of this enzyme in root carbon and nitrogen metabolism. Plant Cell 24, 4044–4065. doi: 10.1105/tpc.112.103689 23054470 PMC3517235

[B27] Gan L. Chen M. Zhang J. Fan J. Yan X. (2022). A novel beta-glucosidase gene for plant type was identified by genome-wide association study and gene Co-expression analysis in widespread bermudagrass. Int. J. Mol. Sci. 23, 11432. doi: 10.3390/ijms231911432 36232734 PMC9570203

[B28] García-Calderón M. Pérez-Delgado C. M. Palove-Balang P. Betti M. Márquez A. J. (2020). Flavonoids and isoflavonoids biosynthesis in the model legume lotus japonicus; connections to nitrogen metabolism and photorespiration. Plants 9, 774. doi: 10.3390/plants9060774 32575698 PMC7357106

[B29] Gau A. Thole H. Sokolenko A. Altschmied L. Herrmann R. Pistorius E. (1998). PsbY, a novel manganese-binding, low-molecular-mass protein associated with photosystem II. Mol. Genet. Genom. 260, 56–68. doi: 10.1007/s004380050870 9829828

[B30] Guo D. Chen F. Inoue K. Blount J. W. Dixon R. A. (2001). Downregulation of caffeic acid 3-o-methyltransferase and caffeoyl CoA 3-o-methyltransferase in transgenic alfalfa: impacts on lignin structure and implications for the biosynthesis of G and s lignin. Plant Cell 13, 73–88. doi: 10.1105/tpc.13.1.73 11158530 PMC102215

[B31] Han Y. Watanabe S. Shimada H. Sakamoto A. (2020). Dynamics of the leaf endoplasmic reticulum modulate β-glucosidase-mediated stress-activated ABA production from its glucosyl ester. J. Exp. Bot. 71, 2058–2071. doi: 10.1093/jxb/erz528 31761937 PMC7094080

[B32] Hao J. Li Q. Yu H. Wang H. Chai L. Miao T.. (2021). Comparative proteomic analysis of cucumber fruits under nitrogen deficiency at the fruiting stage. Hortic. Plant J. 7, 59–72. doi: 10.1016/j.hpj.2020.12.002

[B33] Hirano K. Aya K. Kondo M. Okuno A. Morinaka Y. Matsuoka M. (2012). *OsCAD2* Is the major *CAD* gene responsible for monolignol biosynthesis in rice culm. Plant Cell Rep. 31, 91–101. doi: 10.1007/s00299-011-1142-7 21912859

[B34] Hu T. Wang Y. Wang Q. Dang N. Wang L. Liu C.. (2019). The tomato 2-oxoglutarate-dependent dioxygenase gene *S1F3HL* is critical for chilling stress tolerance. Hortic. Res. 6, 45. doi: 10.1038/s41438-019-0127-5 30962938 PMC6441657

[B35] Imamura S. Kanesaki Y. Ohnuma M. Inouye T. Sekine Y. Fujiwara T.. (2009). R2R3-type MYB transcription factor, CmMYB1, is a central nitrogen assimilation regulator in *Cyanidioschyzon merolae* . Proc. Natl. Acad. Sci. 106, 12548–12553. doi: 10.1073/pnas.090279010 19592510 PMC2718362

[B36] Jia X. Zhu Y. Hu Y. Zhang R. Cheng L. Zhu Z.. (2019). Integrated physiologic, proteomic, and metabolomic analyses of *Malus halliana* adaptation to saline–alkali stress. Hortic. Res. 6, 91. doi: 10.1038/s41438-019-0172-0 31645949 PMC6804568

[B37] Jiao C. Sørensen I. Sun X. Sun H. Behar H. Alseekh S.. (2020). The *Penium margaritaceum* genome: hallmarks of the origins of land plants. Cell 181, 1097–1111. doi: 10.1016/j.cell.2020.04.019 32442406

[B38] Kakiuchi S. Uno C. Ido K. Nishimura T. Noguchi T. Ifuku K.. (2012). The PsbQ protein stabilizes the functional binding of the PsbP protein to photosystem II in higher plants. Biochim. Biophys. Acta Bioenerg. 1817, 1346–1351. doi: 10.1016/j.bbabio.2012.01.009 22306528

[B39] Kanehisa M. Araki M. Goto S. Hattori M. Hirakawa M. Itoh M.. (2008). KEGG for linking genomes to life and the environment. Nucleic Acids Res. 36, D480–D484. doi: 10.1093/nar/gkm882 18077471 PMC2238879

[B40] Kanehisa M. Furumichi M. Tanabe M. Sato Y. Morishima K. (2017). KEGG: new perspectives on genomes, pathways, diseases and drugs. Nucleic Acids Res. 45, D353–DD61. doi: 10.1093/nar/gkw1092 27899662 PMC5210567

[B41] Kanehisa M. Sato Y. Kawashima M. Furumichi M. Tanabe M. (2016). KEGG as a reference resource for gene and protein annotation. Nucleic Acids Res. 44, D457–DD62. doi: 10.1093/nar/gkv1070 26476454 PMC4702792

[B42] Kiba T. Kudo T. Kojima M. Sakakibara H. (2011). Hormonal control of nitrogen acquisition: roles of auxin, abscisic acid, and cytokinin. J. Exp. Bot. 62, 1399–1409. doi: 10.1093/jxb/erq410 21196475

[B43] Kim J. Lee W. J. Vu T. T. Jeong C. Y. Hong S.-W. Lee H. (2017). High accumulation of anthocyanins *via* the ectopic expression of AtDFR confers significant salt stress tolerance in *Brassica napus* l. Plant Cell Rep. 36, 1215–1224. doi: 10.1007/s00299-017-2147-7 28444442

[B44] Klem K. Oravec M. Holub P. Šimor J. Findurová H. Surá K.. (2022). Interactive effects of nitrogen, UV and PAR on barley morphology and biochemistry are associated with the leaf c: n balance. Plant Physiol. Bioch. 172, 111–124. doi: 10.1016/j.plaphy.2022.01.006 35063862

[B45] Krapp A. Traong H. (2006). Regulation of C/N interaction in model plant species. J. Crop Improv. 15, 127–173. doi: 10.1300/J411v15n02_05

[B46] Kumar S. Trivedi P. K. (2018). Glutathione s-transferases: role in combating abiotic stresses including arsenic detoxification in plants. Front. Plant Sci. 9. doi: 10.3389/fpls.2018.00751 PMC5999759 29930563

[B47] Laurent S. Chen H. Bédu S. Ziarelli F. Peng L. Zhang C. (2005). Nonmetabolizable analogue of 2-oxoglutarate elicits heterocyst differentiation under repressive conditions in anabaena sp. PCC 7120. Proc. Natl. Acad. Sci. U.S.A. 102, 9907–9912. doi: 10.1073/pnas.050233710 15985552 PMC1174989

[B48] Lezhneva L. Kiba T. FeriaBourrellier A. B. Lafouge F. BoutetMercey S. Zoufan P.. (2014). The arabidopsis nitrate transporter NRT 2.5 plays a role in nitrate acquisition and remobilization in nitrogen-starved plants. Plant J. 80, 230–241. doi: 10.1111/tpj.12626 25065551

[B49] Li J. Li W. Xu L. Wang M. Zhou W. Li S.. (2022). Acclimation of sugar beet in morphological, physiological and Bv*AMT1. 2* expression under low and high nitrogen supply. PloS One 17, e0278327. doi: 10.1371/journal.pone.0278327 36445927 PMC9707788

[B50] Li J. Liu X. Yao Q. Xu L. Li W. Tan W.. (2023). Tolerance and adaptation characteristics of sugar beet *(Beta vulgaris* l.) to low nitrogen supply. Plant Signal. Behav. 18, 2159155. doi: 10.1080/15592324.2022.2159155 36567601 PMC9794014

[B51] Li M. Xu J. Wang X. Fu H. Zhao M. Wang H.. (2018). Photosynthetic characteristics and metabolic analyses of two soybean genotypes revealed adaptive strategies to low-nitrogen stress. J. Plant Physiol. 229, 132–141. doi: 10.1016/j.jplph.2018.07.009 30081252

[B52] Li Y. Ye Q. He D. Bai H. Wen J. (2020). The ubiquity and coexistence of two FBPases in chloroplasts of photosynthetic eukaryotes and its evolutionary and functional implications. Plant Divers. 42, 120–125. doi: 10.1016/j.pld.2019.09.002 32373770 PMC7195585

[B53] Li L. Ye L. Kong Q. Shou H. (2019). A vacuolar membrane ferric-chelate reductase, OsFRO1, alleviates fe toxicity in rice *(Oryza sativa* l.). Front. Plant Sci. 10. doi: 10.3389/fpls.2019.00700 PMC6558154 31214220

[B54] Liang T. Yuan Z. Fu L. Zhu M. Luo X. Xu W.. (2021). Integrative transcriptomic and proteomic analysis reveals an alternative molecular network of glutamine synthetase 2 corresponding to nitrogen deficiency in rice *(Oryza sativa* l.). Int. J. Mol. Sci. 22, 7674. doi: 10.3390/ijms22147674 34299294 PMC8304609

[B55] Liao H. Chung Y. Hsieh M. (2022). Glutamate: a multifunctional amino acid in plants. Plant Sci. 318, 111238. doi: 10.1016/j.plantsci.2022.111238 35351313

[B56] Lin Y. Pan K. Hung C. Huang H. Chen C. Feng T.. (2013). Overexpression of ferredoxin, PETF, enhances tolerance to heat stress in chlamydomonas reinhardtii. Int. J. Mol. Sci. 14, 20913–20929. doi: 10.3390/ijms141020913 24141188 PMC3821650

[B57] Liu J. Yang H. Lu Q. Wen X. Chen F. Peng L.. (2012). PsbP-domain protein1, a nuclear-encoded thylakoid lumenal protein, is essential for photosystem I assembly in *Arabidopsis* . Plant Cell 24, 4992–5006. doi: 10.1105/tpc.112.106542 23221595 PMC3556971

[B58] Lotta v. S. Serena S. Jörg M. Christiane F. Fikret M. Wolfgang P. S. (2016). The PsbY protein of arabidopsis photosystem II is important for the redox control of cytochrome *b559* . Biochim. Biophys. Acta Bioenerg. 1857, 1524–1533. doi: 10.1016/j.bbabio.2016.05.004 27220875

[B59] Mao X. Cai T. Olyarchuk J. G. Wei L. (2005). Automated genome annotation and pathway identification using the KEGG orthology (KO) as a controlled vocabulary. Bioinformatics 21, 3787–3793. doi: 10.1093/bioinformatics/bti430 15817693

[B60] Meng X. Wang X. Zhang Z. Xiong S. Wei Y. Guo J.. (2021). Transcriptomic, proteomic, and physiological studies reveal key players in wheat nitrogen use efficiency under both high and low nitrogen supply. J. Exp. Bot. 72, 4435–4456. doi: 10.1093/jxb/erab153 33829261

[B61] Nakabayashi R. Saito K. (2015). Integrated metabolomics for abiotic stress responses in plants. Curr. Opin. Plant Biol. 24, 10–16. doi: 10.1016/j.pbi.2015.01.003 25618839

[B62] Nunes-Nesi A. Fernie A. R. Stitt M. (2010). Metabolic and signaling aspects underpinning the regulation of plant carbon nitrogen interactions. Mol. Plant 3, 973–996. doi: 10.1093/mp/ssq049 20926550

[B63] Onaga G. Wydra K. (2016). Advances in plant tolerance to abiotic stresses. Plant Genomics 10, 229–272. doi: 10.5772/64350

[B64] Opassiri R. Pomthong B. Onkoksoong T. Akiyama T. Esen A. Ketudat Cairns J. R. (2006). Analysis of rice glycosyl hydrolase family 1 and expression of Os4bglu12 β-glucosidase. BMC Plant Biol. 6, 1–19. doi: 10.1186/1471-2229-6-33 17196101 PMC1781453

[B65] O’Sullivan J. (2021). “Chapter 12 - climate change and world population,” in The impacts of climate change. Ed. Letcher T. M. (Australia: Elsevier), 313–350.

[B66] Ozawa S.-I. Bald T. Onishi T. Xue H. Matsumura T. Kubo R.. (2018). Configuration of ten light-harvesting chlorophyll a/b complex I subunits in chlamydomonas reinhardtii photosystem I. Plant Physiol. 178, 583–595. doi: 10.1104/pp.18.00749 30126869 PMC6181050

[B67] Pan X. Cao P. Su X. Liu Z. Li M. (2020). Structural analysis and comparison of light-harvesting complexes I and II. Biochim. Biophys. Acta Bioenerg. 1861, 148038. doi: 10.1016/j.bbabio.2019.06.010 31229568

[B68] Pan S. Liu H. Mo Z. Patterson B. Duan M. Tian H.. (2016). Effects of nitrogen and shading on root morphologies, nutrient accumulation, and photosynthetic parameters in different rice genotypes. Sci. Rep. 6, 1–14. doi: 10.1038/srep32148 27557779 PMC4997252

[B69] Passardi F. Longet D. Penel C. Dunand C. (2004). The class III peroxidase multigenic family in rice and its evolution in land plants. Phytochemistry 65, 1879–1893. doi: 10.1016/j.phytochem.2004.06.023 15279994

[B70] Peleman J. D. van der Voort J. R. (2003). Breeding by design. Trends Plant Sci. 8, 330–334. doi: 10.1016/S1360-1385(03)00134-1 12878017

[B71] Peng M. Hannam C. Gu H. Bi Y. M. Rothstein S. J. (2007). A mutation in *NLA*, which encodes a RING-type ubiquitin ligase, disrupts the adaptability of *Arabidopsis* to nitrogen limitation. Plant J. 50, 320–337. doi: 10.1111/j.1365-313X.2007.03050.x 17355433

[B72] Qin L. Walk T. C. Han P. Chen L. Zhang S. Li Y.. (2019). Adaption of roots to nitrogen deficiency revealed by 3D quantification and proteomic analysis. Plant Physiol. 179, 329–347. doi: 10.1104/pp.18.00716 30455286 PMC6324228

[B73] Quan X. Zeng J. Han Z. Zhang G. (2017). Ionomic and physiological responses to low nitrogen stress in Tibetan wild and cultivated barley. Plant Physiol. Bioch. 111, 257–265. doi: 10.1016/j.plaphy.2016.12.008 27951495

[B74] Rana N. K. Mohanpuria P. Kumar V. Yadav S. K. (2010). A CsGS is regulated at transcriptional level during developmental stages and nitrogen utilization in *Camellia sinensis* (L.) o. kuntze. Mol. Biol. Rep. 37, 703–710. doi: 10.1007/s11033-009-9559-6 19449127

[B75] Ren C. Jin S. Wu Y. Zhang B. Kanter D. Wu B.. (2021). Fertilizer overuse in Chinese smallholders due to lack of fixed inputs. J. Environ. Manage. 293, 112913. doi: 10.1016/j.jenvman.2021.112913 34091142

[B76] Schmitter J. Jacquot J. Frédéric D. L. Beauvallet C. Dutka S. Gadal P.. (1988). Purification, properties and complete amino acid sequence of the ferredoxin from a green alga, *Chlamydomonas reinhardtii* . Eur. J. Biochem. 172, 405–412. doi: 10.1111/j.1432-1033.1988.tb13901.x 3350005

[B77] Shen X. Yang L. Han P. Gu C. Li Y. Liao X.. (2022). Metabolic profiles reveal changes in the leaves and roots of rapeseed *(Brassica napus* l.) seedlings under nitrogen deficiency. Int. J. Mol. Sci. 23, 5784. doi: 10.3390/ijms23105784 35628591 PMC9142919

[B78] Shi Y. Guo E. Cheng X. Wang L. Jiang S. Yang X.. (2022). Effects of chilling at different growth stages on rice photosynthesis, plant growth, and yield. Environ. Exp. Bot. 203, 105045. doi: 10.1016/j.envexpbot.2022.105045

[B79] Silva F. A. Albuquerque L. M. Martins T. F. de Freitas J. A. Vasconcelos I. M. de Freitas D. Q.. (2022). A peroxidase purified from cowpea roots possesses high thermal stability and displays antifungal activity against *Colletotrichum gloeosporioides* and *Fusarium oxysporum* . Biocatal. Agric. Biotechnol. 42, 102322. doi: 10.1016/j.bcab.2022.102322

[B80] Simões W. L. Yuri J. E. Guimarães M. J. Santos J. Araújo E. F. (2016). Beet cultivation with saline effluent from fish farming. Rev. Bras. Eng. Agr. Amb. 20, 62–66. doi: 10.1590/1807-1929/agriambi.v20n1p62-66

[B81] Sultana N. Islam S. Juhasz A. Yang R. She M. Alhabbar Z.. (2020). Transcriptomic study for identification of major nitrogen stress responsive genes in Australian bread wheat cultivars. Front. Genet. 11. doi: 10.3389/fgene.2020.583785 PMC7554635 33193713

[B82] Sun H. Shi Q. Zhang S. Huang W. (2022). The response of photosystem I to fluctuating light is influenced by leaf nitrogen content in tomato. Environ. Exp. Bot. 193, 104665. doi: 10.1016/j.envexpbot.2021.104665

[B83] Sweetlove L. J. Beard K. F. Nunes-Nesi A. Fernie A. R. Ratcliffe R. G. (2010). Not just a circle: flux modes in the plant TCA cycle. Trends Plant Sci. 15, 462–470. doi: 10.1016/j.tplants.2010.05.006 20554469

[B84] Sweetlove L. J. Fernie A. R. (2013). The spatial organization of metabolism within the plant cell. Annu. Rev. Plant Biol. 64, 723–746. doi: 10.1146/annurev-arplant-050312-120233 23330793

[B85] Tantray A. Y. Hazzazi Y. Ahmad A. (2022). Physiological, agronomical, and proteomic studies reveal crucial players in rice nitrogen use efficiency under low nitrogen supply. Int. J. Mol. Sci. 23, 6410. doi: 10.3390/ijms23126410 35742855 PMC9224494

[B86] Terauchi A. M. Lu S. Zaffagnini M. Tappa S. Hirasawa M. Tripathy J. N.. (2009). Pattern of expression and substrate specificity of chloroplast ferredoxins from chlamydomonas reinhardtii. J. Biol. Chem. 284, 25867–25878. doi: 10.1074/jbc.M109.023622 19586916 PMC2757988

[B87] Tercé-Laforgue T. Bedu M. Dargel-Grafin C. Dubois F. Gibon Y. Restivo F. M.. (2013). Resolving the role of plant glutamate dehydrogenase: II. physiological characterization of plants overexpressing the two enzyme subunits individually or simultaneously. Plant Cell Physiol. 54, 1635–1647. doi: 10.1093/pcp/pct108 23893023

[B88] Tercé-Laforgue T. Clément G. Marchi L. Restivo F. M. Lea P. J. Hirel B. (2015). Resolving the role of plant NAD-glutamate dehydrogenase: III. overexpressing individually or simultaneously the two enzyme subunits under salt stress induces changes in the leaf metabolic profile and increases plant biomass production. Plant Cell Physiol. 56, 1918–1929. doi: 10.1093/pcp/pcv114 26251210

[B89] Tian H. Song H. Wu X. Zhang Z. (2022). Responses of cell wall components to low nitrogen in rapeseed roots. Agronomy 12, 1044. doi: 10.3390/agronomy12051044

[B90] Wan Q. Bai T. Liu M. Liu Y. Xie Y. Zhang T.. (2022). Comparative analysis of the chalcone-flavanone lsomerase genes in six citrus species and their expression analysis in sweet orange *(Citrus sinensis*). Front. Genet. 13:848141. doi: 10.3389/fgene.2022.848141 35495138 PMC9039136

[B91] Wang H. Fan W. Li H. Yang J. Huang J. Zhang P. (2013). Functional characterization of dihydroflavonol-4-reductase in anthocyanin biosynthesis of purple sweet potato underlies the direct evidence of anthocyanins function against abiotic stresses. PloS One 8, e78484. doi: 10.1371/journal.pone.0078484 24223813 PMC3817210

[B92] Wascher F. L. Stralis-Pavese N. McGrath J. M. Schulz B. Himmelbauer H. Dohm J. C. (2022). Genomic distances reveal relationships of wild and cultivated beets. Nat. Commun. 13, 2021. doi: 10.1038/s41467-022-29676-9 35440134 PMC9019029

[B93] Wen B. Gong X. Chen X. Tan Q. Li L. Wu H. (2022). Transcriptome analysis reveals candidate genes involved in nitrogen deficiency stress in apples. J. Plant Physiol. 279, 153822. doi: 10.1016/j.jplph.2022.153822 36244263

[B94] Xu C. Huang S. Tian B. Ren J. Meng Q. Wang P. (2017). Manipulating planting density and nitrogen fertilizer application to improve yield and reduce environmental impact in Chinese maize production. Front. Plant Sci. 8. doi: 10.3389/fpls.2017.01234 PMC5506086 28747925

[B95] Yamazaki S. Ochiai K. Matoh T. (2019). Rice plants have three homologs of glutathione synthetase genes, one of which, OsGS2, codes for hydroxymethyl-glutathione synthetase. Plant direct 3, e00119. doi: 10.1002/pld3.119 31245762 PMC6508825

[B96] Yang T. Zhang P. Pan J. Amanullah S. Luan F. Han W.. (2022). Genome-wide analysis of the peroxidase gene family and verification of lignin synthesis-related genes in watermelon. Int. J. Mol. Sci. 23, 642. doi: 10.3390/ijms23020642 35054827 PMC8775647

[B97] Yi X. Hargett S. R. Frankel L. K. Bricker T. M. (2009). The PsbP protein, but not the PsbQ protein, is required for normal thylakoid architecture in *Arabidopsis thaliana* . FEBS Lett. 583, 2142–2147. doi: 10.1016/j.febslet.2009.05.048 19500580

[B98] Yi X. Hargett S. R. Liu H. Frankel L. K. Bricker T. M. (2007). The PsbP protein is required for photosystem II complex assembly/stability and photoautotrophy in *Arabidopsis thaliana* . J. Biol. Chem. 282, 24833–24841. doi: 10.1074/jbc.M705011200 17604269

[B99] Yin L. Xu H. Dong S. Chu J. Dai X. He M. (2019). Optimised nitrogen allocation favours improvement in canopy photosynthetic nitrogen-use efficiency: evidence from late-sown winter wheat. Environ. Exp. Bot. 159, 75–86. doi: 10.1016/j.envexpbot.2018.12.013

[B100] Young M. D. Wakefield M. J. Smyth G. K. Oshlack A. (2010). Gene ontology analysis for RNA-seq: accounting for selection bias. Genome Biol. 11, R14. doi: 10.1186/gb-2010-11-2-r14 20132535 PMC2872874

[B101] Yu W. Liu H. Luo J. Zhang S. Xiang P. Wang W.. (2022). Partial root-zone simulated drought induces greater flavonoid accumulation than full root-zone simulated water deficiency in the leaves of *Ginkgo biloba.* environ. Exp. Bot. 201, 104998. doi: 10.1016/j.envexpbot.2022.104998

[B102] Zhang X. Davidson E. A. Mauzerall D. L. Searchinger T. D. Dumas P. Shen Y. (2015). Managing nitrogen for sustainable development. Nature 528, 51–59. doi: 10.1038/nature15743 26595273

[B103] Zhang X. Gong X. Yu H. Su X. Cheng S. Huang J.. (2023). The proline-rich protein MdPRP6 confers tolerance to salt stress in transgenic apple *(Malus domestica*). Sci. Hortic. 308, 111581. doi: 10.1016/j.scienta.2022.111581

[B104] Zhang X. Misra A. Nargund S. Coleman G. D. Sriram G. (2018c). Concurrent isotope-assisted metabolic flux analysis and transcriptome profiling reveal responses of poplar cells to altered nitrogen and carbon supply. Plant J. 93, 472–488. doi: 10.1111/tpj.13792 29193384

[B105] Zhang Q. Tang D. Liu M. Ruan J. (2018b). Integrated analyses of the transcriptome and metabolome of the leaves of albino tea cultivars reveal coordinated regulation of the carbon and nitrogen metabolism. Sci. Hortic. 231, 272–281. doi: 10.1016/j.scienta.2017.11.026

[B106] Zhang C. Zhou C. Burnap R. L. Peng L. (2018a). Carbon/nitrogen metabolic balance: lessons from cyanobacteria. Trends Plant Sci. 23, 1116–1130. doi: 10.1016/j.tplants.2018.09.008 30292707

[B107] Zhao Q. (2016). Lignification: flexibility, biosynthesis and regulation. Trends Plant Sci. 21, 713–721. doi: 10.1016/j.tplants.2016.04.006 27131502

[B108] Zheng J. Li S. Maratab A. Huang Q. Zhneg X. Pang L. (2020). Effects of UV-b treatment on controlling lignification and quality of bamboo *(Phyllostachys prominens*) shoots without sheaths during cold storage. J. Integr. Agric. 19, 1387–1395. doi: 10.1016/S2095-3119(20)63170-7

