# Supplementary material for: Low nitrogen stress-induced transcriptome changes revealed the molecular response and tolerance characteristics in maintaining the C/N balance of sugar beet (Beta vulgaris L.)
Source: Front Plant Sci. 2023 Apr 21;14:1164151. doi: 10.3389/fpls.2023.1164151 (PMC10160481; doi:10.3389/fpls.2023.1164151)
Supplement: Supplementary file 1 [file DataSheet_1.docx]

Supplementary Material

**Low nitrogen stress induced transcriptome changes revealed** the molecular response and tolerance characteristics in maintaining C/N balance of sugar beet (***Beta vulgaris*** L.)

#### Jiajia Li^1,2＃^, Xinyu Liu^1,2,3^, Lingqing Xu^1,2^, Wangsheng Li^1,2^, Qi Yao^1,2,3^, Xilong Yin^1,2^, Qiuhong Wang^1,2^, Wenbo Tan^1,2^, Wang Xing^1,2^ and Dali Liu^1,2＃*^

^1^ National Beet Medium-term Gene Bank, Heilongjiang University, Harbin, 150080, P. R. China;

^2^ Key Laboratory of Sugar Beet Genetics and Breeding, Heilongjiang Province Common College/College of Advanced agriculture and ecological environment, Heilongjiang University, Harbin, 150080, P. R. China;

^3^ Key Laboratory of Molecular Biology, School of Life Sciences, Heilongjiang University, Harbin, China.

^＃^ These authors contributed equally to this work.

*** Correspondence:**

Dali Liu**:** daliliu_hlju@163.com;

Wang Xing: xyjiayou_086@163.com

Tel.: +86 451 86609494

# Supplementary Figures


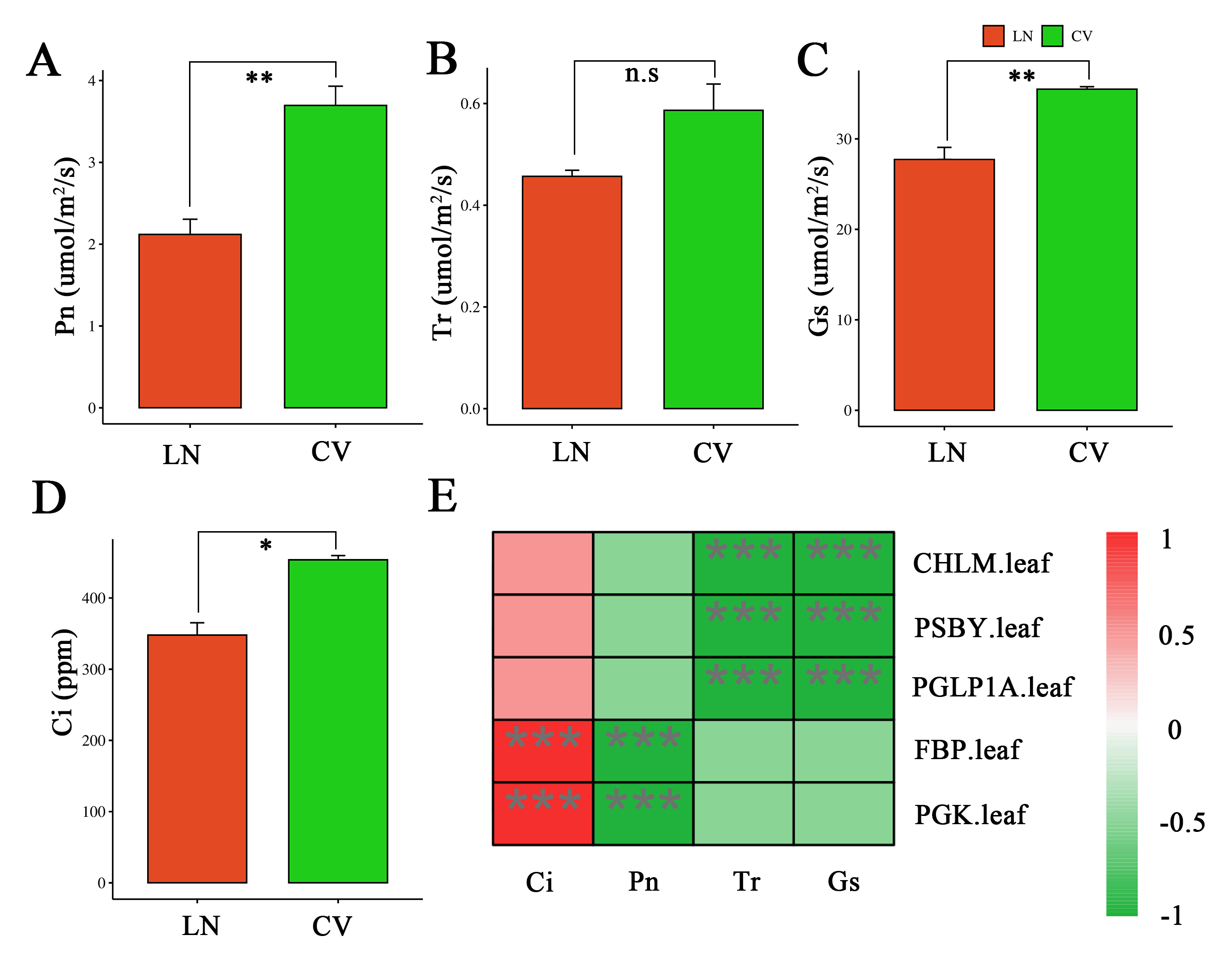


**Supplementary Figure 1. Apparent photosynthetic indexes of sugar beet seedling leaves under LN.** A. Net photosynthetic rate (Pn), B. Transpiration rate (Tr), C. Stomatal conductivity (Gs), D. Intercellular CO_2_ concentration (Ci), E. Correlation analysis of photosynthetic indexes with DEGs. *, **and*** indicate significant differences at p < 0.05, p < 0.01 and p < 0.001, respectively; n.s represents no significant difference.

**
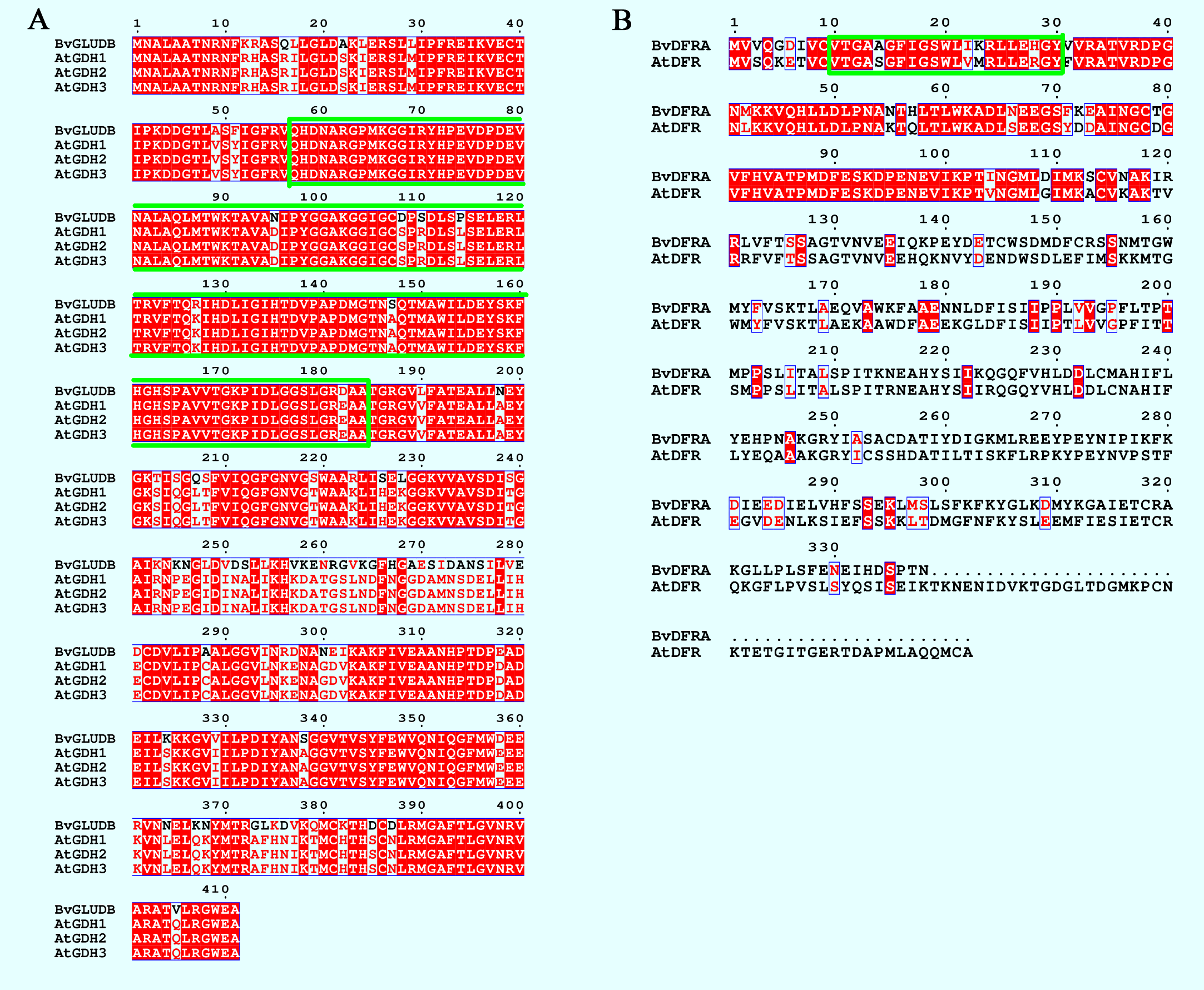
**

**Supplementary Figure 2. Comparative amino acid sequence analysis of BvGLUDB (A) and BvFDRA (B) with homolog proteins from other species.** The conserved functional domains in green boxes are Glu/Leu/Phe/Val dehydrogenase, dimerization domain (InterPro:IPR006097) (A) and the putative NAD(P) binding site (B). AtGDH1 (Locus: AT5G18170), AtGDH2 (Locus: At5g07440), AtGDH3 (Locus:At3g03910), AtDFR (Locus: BAD95233).

# Supplementary Tables

**Supplementary Table 1. Primers for qRT-PCR**

| **Gene id** | **Gene name** | **Forward primer (5'-3')** | **Reverse primer (5'-3')** |
| --- | --- | --- | --- |
| Reference gene | GAPDH (LOC104893518) | GCTTTGAACGACCACTTCGC | ACGCCGAGAGCAACTTGAAC |
| 104884920 | PDP | CCTGACTTTGGTTTGCTCACAACTG | TCTCCATCAACAACGACGCAC |
| 104883662 | CAB | GCCATGTTTTCGATGTTCGGA | ACTTCACTTTACTTGCCGGG |
| 104884524 | NRT3.2 | GTGGTCGTAGCACCTCCATC | AAATGCGAGAGAGAGCACGG |
| 104891570 | NIR | GGCTGGAATCTGTTGGCCTT | TCCCTGCAAGAGGATTACCA |
| 104900882 | TDT | CGTCGAAGAGACAACTCTAGGC | TGAGAGAAATAAGCAAGGGACAA |
| 104897276 | GGAT2 | GGGCAGAAAGAAGGGGTGTT | AGCTGTCCATGATAGCAGGC |
| 104888349 | AGL | ACATTTGTACCACGACTACTGCT | ACCCTCGATAAATGTTGAGCTT |
| 104887841 | NR | TGGCCGTATGGTTAAGTGGT | GCTTGTACCACCAAGCTTCAG |
| 104888010 | 4CL2 | AGGCTATTTTGGGTCAGGGC | AGAGGCTCCTTGGCAAATCC |
| 104895023 | VPS39 | GAAGAGTCAAAGGCAGGCCA | TGTCCCACAAAGAGGCTTCA |
| 104892090 | AMO | GGACCATTTTGGAGCGTGGA | TCTTGCCGTACCACCCTGTA |
| 104908380 | CYP76AD1 | GCGCCTGATTCTTCAACTGC | AGCATCAAAAATGTCGACGAGC |
| 104897077 | U2AF65B | ATGACTCAGCAGATGGCTGT | ACTTGCTGAGCTTCGGAGAG |
| 104892484 | CYP72A397 | ACTTGTCACAAAAGCTGGATCA | GTGGGTTCATTTTTGGCTTCCT |
| 104903619 | GAT1 | CAACGTACTCCAGCGACAGT | CAGTGCACCCATGATCCTTT |
| 104889718 | TOP3B | GCAACAGTTCTTTCTCGTGGT | TGGAATGTTGCAGGAAAATCTGA |
| 104893230 | IMT1 | TGCTCCTTCATATCCTGGTGTG | TAAGCAATGCTCGTCGCTCC |
| 104905708 | BURP5 | TGCTGCTTTTCTCGGTTGGA | TGGCATTGGGGTGAAAGGAA |
| 104884526 | QS | ATTGCCTCTTTGTCACCACCT | ATCTGTGCAAATGCCTGCAAC |
| 104888277 | CNGC1 | TGGATTTAGTTACAAGGGTCCCA | CTTCAGGCGGTCACACATTG |
| 104904631 | GA3OX1 | CCCCAAGAGGATGTGGTCTG | GGTACTCCTCAATAATGTCACAGAA |
| 104898402 | FBL11 | GGATGCCACGTGGAAGTTAC | TCCAAAGTACAGTGCAGCCTTA |

**Supplementary Table 2. Basic statistics of sequencing reads compared to the reference genome in sugar beet under LN stress**

| **Sample name** | **Raw reads** | **Clean reads** | **Error rate (%)** | **Q20 (%)** | **Q30 (%)** | **GC content (%)** | **Comparison rate (%)** |
| --- | --- | --- | --- | --- | --- | --- | --- |
| CV_L1 | 86174406 | 84587316 | 0.01 | 97.77 | 94.25 | 41.27 | 99 |
| CV_L2 | 100442240 | 96518428 | 0.01 | 97.87 | 94.48 | 42.01 | 99 |
| CV_L3 | 91153422 | 89019028 | 0.01 | 97.9 | 94.52 | 42.28 | 99 |
| CV_R1 | 89551558 | 85004098 | 0.02 | 97.01 | 92.66 | 41.86 | 98 |
| CV_R2 | 104136150 | 99318914 | 0.01 | 97.82 | 94.36 | 42.52 | 99 |
| CV_R3 | 96585642 | 92393488 | 0.01 | 97.78 | 94.2 | 42.33 | 99 |
| LN_L1 | 88080704 | 86249904 | 0.01 | 97.75 | 94.22 | 41.08 | 99 |
| LN_L2 | 92896570 | 90789106 | 0.01 | 97.75 | 94.27 | 41.41 | 99 |
| LN_L3 | 89373726 | 86505160 | 0.02 | 97.25 | 92.93 | 41.34 | 98 |
| LN_R1 | 96714870 | 92617680 | 0.01 | 97.77 | 94.19 | 42.66 | 99 |
| LN_R2 | 93291348 | 89099236 | 0.01 | 97.91 | 94.51 | 42.7 | 99 |
| LN_R3 | 101928710 | 96891728 | 0.01 | 97.9 | 94.47 | 43.15 | 99 |

**Supplementary Table 3. DEGs of the top 20 significantly up- and down-regulated in sugar beet responsive to LN**

|  | **Transcript_id** | **Gene_id** | **Locus tag** | **Gene name** | **Gene description** | **Log2(fold change)** | **q value** |
| --- | --- | --- | --- | --- | --- | --- | --- |
| **The top 20 genes significantly up-regulated in the root** | XM_010694132.1 | 104905546 | - | - | protein NRT1/ PTR FAMILY 7.3-like | 9.814988092 | 3.67318E-17 |
|  | XM_010682152.2 | 104895595 | - | - | L-ascorbate oxidase homolog | 9.655561033 | 0.026257207 |
|  | XM_010673539.2 | 104888523 | BVRB_3g055010 | LSI2 | silicon efflux transporter LSI2 | 8.286235513 | 0.023984958 |
|  | XM_019251454.1 | 104902481 | - | MLP34 | MLP-like protein 34 | 7.779980698 | 0.000974073 |
|  | XM_010693891.2 | 104905365 | BVRB_1g018740 | CHS2 | chalcone synthase 3 | 7.478293343 | 5.63885E-31 |
|  | XR_789593.2 | 104889626 | BVRB_3g063950 | RGA3 | putative disease resistance protein RGA3 | 7.449646381 | 6.87034E-11 |
|  | XM_010668698.2 | 104884102 | - | - | uncharacterized LOC104884102 | 7.369644035 | 0.001419658 |
|  | XM_010680317.2 | 104894139 | BVRB_5g115520 | - | hypothetical protein | 7.345282421 | 1.04957E-08 |
|  | XM_019250064.1 | 104895023 | BVRB_6g128740 | VPS39 | vacuolar sorting protein 39 | 7.294863323 | 0.007260255 |
|  | XM_010682681.2 | 104896014 | BVRB_6g138460 | Os01g0723500 | B3 domain-containing protein Os01g0723500 | 7.278546095 | 0.001206879 |
|  | XM_010682328.2 | 104895746 | BVRB_6g135980 | - | hypothetical protein | 6.804389965 | 0.002476782 |
|  | XM_010677915.2 | 104892090 | BVRB_5g098290 | AMO | primary amine oxidase | 6.70197823 | 1.69944E-50 |
|  | XM_010693892.2 | 104905366 | BVRB_1g018750 | CHS2 | chalcone synthase 3 | 6.548582197 | 1.17138E-20 |
|  | XM_010687683.2 | 104900293 | BVRB_7g175850 | DHPS1 | 4-hydroxy-tetrahydrodipicolinate synthase, chloroplastic | 6.529366821 | 0.002696341 |
|  | XM_010697501.2 | 104908380 | BVRB_2g029930 | CYP76AD1 | cytochrome P450 76AD1 | 6.427969927 | 6.03865E-07 |
|  | XR_002038917.1 | 109133758 | - | - | uncharacterized LOC109133758 | 6.331910746 | 0.029029162 |
|  | XM_010674757.2 | 104889519 | BVRB_3g063250 | At3g47110 | putative receptor-like protein kinase At3g47110 | 6.30757588 | 0.000222218 |
|  | XM_010674241.2 | 104889096 | BVRB_1g006520 | Os02g0491600 | putative germin-like protein 2-1 | 6.288448772 | 0.003369562 |
|  | XM_010692724.2 | 104904469 | BVRB_9g218660 | - | hypothetical protein | 6.263014295 | 0.00495151 |
|  | XM_019250645.1 | 104900396 | - | STPS | probable sesquiterpene synthase | 6.155641789 | 3.36059E-09 |
| **The top 20 genes significantly down-regulated in the root** | XM_019247252.1 | 104883006 | BVRB_8g201410 | - | tropinone reductase-like 3 | -7.1117636 | 0.000702447 |
|  | XM_019249633.1 | 104893771 | BVRB_5g112500 | ATJ72 | chaperone protein dnaJ 72 | -7.154740388 | 0.005216069 |
|  | XM_010681864.2 | 104895379 | BVRB_6g132310 | PCMP-E95 | pentatricopeptide repeat-containing protein At3g22150, chloroplastic | -7.214961579 | 0.037665023 |
|  | XM_010685350.2 | 104898294 | BVRB_7g158760 | CYP72A397 | cytochrome P450 72A397 | -7.225926672 | 1.73716E-05 |
|  | XM_010671676.2 | 104887088 | BVRB_1g004230 | FRO2 | ferric reduction oxidase 2 | -7.424254367 | 1.14924E-38 |
|  | XM_010672874.2 | 104888048 | BVRB_3g050240 | CRP1 | pentatricopeptide repeat-containing protein At5g42310, chloroplastic | -7.476876531 | 0.000140109 |
|  | XM_010667446.1 | 104883006 | BVRB_8g201410 | - | tropinone reductase-like 3 | -7.53738017 | 0.002185292 |
|  | XM_019247712.1 | 104884934 | BVRB_013210 | ALA2 | phospholipid-transporting ATPase 2 | -7.575125374 | 0.044559851 |
|  | XM_019247971.1 | 104886773 | BVRB_1g003760 | At3g61590 | F-box/kelch-repeat protein At3g61590 | -7.707588245 | 0.000113558 |
|  | XR_002039064.1 | 104884940 | BVRB_013230 | PCMP-H44 | pentatricopeptide repeat-containing protein At2g03880, mitochondrial | -7.711896076 | 0.030438623 |
|  | XM_010685351.2 | 104898297 | BVRB_7g158790 | CYP72A397 | cytochrome P450 72A397 | -7.794129349 | 6.91118E-07 |
|  | XM_010692371.2 | 104904176 | BVRB_9g212980 | EXLA3 | expansin-like A3 | -8.046455267 | 3.09735E-08 |
|  | XR_002039068.1 | 104884940 | BVRB_013230 | PCMP-H44 | pentatricopeptide repeat-containing protein At2g03880, mitochondrial | -8.066362045 | 0.029863866 |
|  | XM_010688367.1 | 104900837 | - | ZNT7 | zinc transporter 7-like | -8.08490297 | 2.59231E-09 |
|  | XR_002039854.1 | 104888135 | BVRB_1g000110 | APF1 | aspartyl protease family protein 1 | -8.342694416 | 4.31645E-06 |
|  | XR_002040881.1 | 104902821 | BVRB_9g205070 | THA8L | protein THYLAKOID ASSEMBLY 8-like, chloroplastic | -8.376748433 | 0.000169824 |
|  | XM_010691744.2 | 104903667 | BVRB_9g211320 | PPA2 | inorganic pyrophosphatase 2 | -9.242075073 | 0.001619349 |
|  | XR_002039491.1 | 104888667 | BVRB_3g055810 | PSI2 | protein PSK SIMULATOR 2 | -11.04877156 | 0.014561287 |
|  | XM_019250336.1 | 104896949 | BVRB_6g145180 | At5g59900 | putative pentatricopeptide repeat-containing protein At5g59900 | -11.06645204 | 0.036738528 |
|  | XM_010683892.2 | 104897077 | BVRB_6g146140 | U2AF65B | splicing factor U2af large subunit B | -14.90717434 | 0.001663179 |
| **The top 20 genes significantly up-regulated in the leaf** | XM_010696516.2 | 104907565 | BVRB_2g028960 | At3g52030 | F-box/WD-40 repeat-containing protein At3g52030 | 13.4643836 | 0.001309812 |
|  | XM_019249651.1 | 104893939 | BVRB_5g113620 | BIM2 | transcription factor BIM2 | 12.62069774 | 3.12854E-05 |
|  | XM_010697670.2 | 104908549 | BVRB_7g176890 | At4g24790 | protein STICHEL-like 2 | 12.09587181 | 0.037103208 |
|  | XM_010674610.2 | 104889399 | BVRB_3g062020 | SUVH6 | histone-lysine N-methyltransferase, H3 lysine-9 specific SUVH6 | 11.72838931 | 0.000603745 |
|  | XM_019251340.1 | 104902402 | BVRB_1g002090 | BHLH67 | transcription factor bHLH67 | 10.58015258 | 0.02228522 |
|  | XM_019251039.1 | 104900248 | BVRB_7g175380 | At2g30100 | pentatricopeptide repeat-containing protein At2g30100, chloroplastic | 9.281534052 | 0.020513196 |
|  | XM_010669554.2 | 104884853 | BVRB_012730 | At5g03100 | F-box protein At5g03100 | 9.255080053 | 0.005039604 |
|  | XM_019250064.1 | 104895023 | BVRB_6g128740 | VPS39 | vacuolar sorting protein 39 | 9.069192246 | 0.007107631 |
|  | XM_010690941.2 | 104902978 | BVRB_9g206180 | - | uncharacterized LOC104902978 | 8.985244002 | 0.014742755 |
|  | XM_010680682.2 | 104894454 | BVRB_5g118860 | DDX42 | DEAD-box ATP-dependent RNA helicase 42 | 8.854424129 | 0.017467072 |
|  | XM_010678432.2 | 104892484 | BVRB_5g102640 | CYP72A397 | cytochrome P450 72A397 | 8.068689017 | 0.002705852 |
|  | XM_010692165.2 | 104903997 | BVRB_9g214790 | DPH6 | diphthine--ammonia ligase | 7.985773966 | 0.007975723 |
|  | XM_010672674.2 | 104887896 | BVRB_1g005130 | CYP71AZ3 | 6,7,8-trihydroxycoumarin synthase | 7.814591359 | 0.000953419 |
|  | XM_019251867.1 | 104903619 | BVRB_9g210940 | GAT1 | GABA transporter 1 | 7.784218658 | 0.012100785 |
|  | XM_019247124.1 | 104908867 | BVRB_7g179380 | At2g48060 | piezo-type mechanosensitive ion channel homolog | 7.768805976 | 0.010172736 |
|  | XM_010684274.2 | 104897405 | BVRB_6g149040 | BALDH | benzaldehyde dehydrogenase, mitochondrial | 7.573836675 | 6.73133E-05 |
|  | XM_010690021.2 | 104902296 | - | CPR30 | F-box protein CPR30 | 7.499362538 | 0.000574734 |
|  | XM_010680324.2 | 104894145 | BVRB_5g115640 | EMB1027 | arginine--tRNA ligase, chloroplastic/mitochondrial | 7.028159202 | 0.020024257 |
|  | XM_010675015.2 | 104889718 | BVRB_3g065060 | TOP3B | DNA topoisomerase 3-beta | 7.003651858 | 0.028092281 |
|  | XM_010679335.2 | 104893230 | BVRB_5g109500 | IMT1 | inositol 4-methyltransferase | 6.792861279 | 5.29515E-11 |
| **The top 20 genes significantly down-regulated in the leaf** | XM_010692377.1 | 104904181 | BVRB_9g213040 | SAUR64 | auxin-responsive protein SAUR64 | -5.424198827 | 0.020323776 |
|  | XM_010687942.2 | 104900518 | BVRB_8g181230 | - | uncharacterized LOC104900518 | -5.447828382 | 1.26236E-05 |
|  | XM_010667995.2 | 104883464 | BVRB_001340 | ABP19B | auxin-binding protein ABP19b | -5.468224307 | 0.007777957 |
|  | XM_010670918.2 | 104886469 | BVRB_2g033220 | Os02g0224100 | probable protein phosphatase 2C 12 | -5.624820787 | 5.38655E-17 |
|  | XM_010692699.2 | 104904451 | BVRB_9g218520 | - | hypothetical protein | -5.735119814 | 4.16124E-17 |
|  | XM_010694316.2 | 104905708 | BVRB_1g021520 | BURP5 | BURP domain-containing protein 5 | -5.771004329 | 5.09122E-05 |
|  | XM_019247621.1 | 104884526 | BVRB_009200 | QS | quinolinate synthase, chloroplastic | -6.023351234 | 6.96137E-18 |
|  | XM_019248514.1 | 104888277 | BVRB_3g052330 | CNGC1 | cyclic nucleotide-gated ion channel 1 | -6.087200642 | 0.004060039 |
|  | XM_010692935.2 | 104904631 | BVRB_9g220330 | GA3OX1 | gibberellin 3-beta-dioxygenase 1 | -6.224776698 | 3.95378E-05 |
|  | XM_010678183.2 | 104892295 | BVRB_5g100540 | At3g14260 | protein LURP-one-related 11 | -6.249812907 | 0.002216484 |
|  | XM_010697350.2 | 104908232 | BVRB_1g002840 | - | uncharacterized LOC104908232 | -6.454330095 | 1.7532E-17 |
|  | XM_010668015.1 | 104883485 | BVRB_001330 | ABP19B | auxin-binding protein ABP19b | -6.599040272 | 0.015525987 |
|  | XM_010690282.2 | 104902495 | BVRB_8g198750 | PAAS | phenylacetaldehyde synthase | -6.779114311 | 0.002686135 |
|  | XR_002040763.1 | 104902383 | BVRB_8g198300 | - | hypothetical protein | -7.027862657 | 0.008242193 |
|  | XM_010688412.2 | 104900882 | BVRB_8g184880 | TDT | tonoplast dicarboxylate transporter | -7.844247983 | 4.66277E-18 |
|  | XM_019250755.1 | 104898402 | BVRB_7g159950 | FBL11 | BTB/POZ domain-containing protein FBL11 | -8.68965805 | 1.37059E-16 |
|  | XM_010698298.2 | 104909100 | BVRB_8g199660 | ENDO4 | endonuclease 4 | -9.855234334 | 0.017024232 |
|  | XM_010673189.2 | 104888276 | BVRB_3g052320 | - | uncharacterized LOC104888276 | -10.66646481 | 0.048936432 |
|  | XM_019251428.1 | 104902061 | BVRB_8g194720 | At1g55270 | F-box/kelch-repeat protein At1g55270 | -11.49108036 | 0.003121075 |
|  | XM_019247971.1 | 104886773 | BVRB_1g003760 | At3g61590 | F-box/kelch-repeat protein At3g61590 | -13.56205319 | 0.039785112 |

**Supplementary Table 4. Annotation information of down-regulated and up-regulated hub genes of each pathway in sugar beet under LN stress**

|  | **Term** | **Locus tag** | **Gene id** | **Transcript_id** | **Gene name** | **Gene description** | **Part** | **Log2(fold change)** | **q value** |
| --- | --- | --- | --- | --- | --- | --- | --- | --- | --- |
| **Down-hub genes** |  |  |  |  |  |  |  |  |  |
| bvg00195 | Photosynthesis | BVRB_6g147470 | 104897231 | XM_010684065.2 | PsbY | photosystem II core complex proteins psbY, chloroplastic | leaf | -1.11500069 | 0.008430948 |
| bvg00196 | Photosynthesis - antenna proteins | BVRB_5g114540 | 104894032 | XM_010680190.2 | CAB4/Lhca4 | chlorophyll a-b binding protein 4, chloroplastic | leaf | -2.710969332 | 1.93053E-09 |
| bvg01100 | Metabolic pathways | BVRB_1g000370 | 104887841 | XM_010672627.2 | NR | nitrate reductase [NADH] | leaf | -3.336381512 | 1.00517E-13 |
| bvg00710 | Carbon fixation in photosynthetic organisms | BVRB_1g011460 | 104895737 | XM_010682320.2 | FBP | fructose-1,6-bisphosphatase, cytosolic | leaf | -1.236729707 | 0.00403044 |
| bvg01200 | Carbon metabolism | BVRB_2g038200 | 104887103 | XM_010671692.2 | PGK | phosphoglycerate kinase, chloroplastic | leaf | -1.091270835 | 0.008547566 |
| bvg00630 | Glyoxylate and dicarboxylate metabolism | BVRB_4g074740 | 104890185 | XM_010675576.2 | PGLP1A | phosphoglycolate phosphatase 1A, chloroplastic | leaf | -1.126296662 | 0.014092018 |
| bvg01110 | Biosynthesis of secondary metabolites | BVRB_7g180360 | 104908971 | XM_010698139.2 | CHLM | magnesium protoporphyrin IX methyltransferase, chloroplastic | leaf | -1.201324847 | 0.018725366 |
| bvg00910 | Nitrogen metabolism | BVRB_4g087070 | 104891570 | XM_010677274.2 | NIR | ferredoxin--nitrite reductase, chloroplastic | leaf | -2.098912269 | 2.43871E-06 |
|  |  |  |  | XM_010677274.2 | NIR |  | root | -3.267800236 | 1.40255E-12 |
|  |  | BVRB_1g000370 | 104887841 | XM_010672627.2 | NR | nitrate reductase [NADH] | root | -1.46205645 | 0.00013639 |
| **Up-hub genes** |  |  |  |  |  |  |  |  |  |
| bvg01110 | Metabolic pathways | BVRB_005400 | 104884022 | XM_010668612.2 | CAD6 | probable cinnamyl alcohol dehydrogenase 6 | root | 2.343318013 | 0.00100397 |
| bvg01100 | Metabolic pathways | BVRB_2g025900 | 104905171 | XM_010693624.2 | POX2 | proline dehydrogenase 2, mitochondrial | leaf | 3.130558291 | 5.19449E-05 |
|  |  |  |  |  |  |  | root | 2.930252587 | 3.90816E-07 |
| bvg00941 | Flavonoid biosynthesis | BVRB_1g007170 | 104889670 | XM_010674949.2 | DFRA | dihydroflavonol 4-reductase | root | 3.89551494 | 0.01495618 |
|  |  | BVRB_2g036880 | 104886918 | XM_010671471.1 | CHI | chalcone--flavanone isomerase | root | 2.746736996 | 7.08205E-05 |
|  |  | BVRB_2g036940 | 104886934 | XM_010671488.2 | CHI | chalcone--flavanone isomerase | root | 2.576304597 | 2.22423E-08 |
| bvg00940 | Phenylpropanoid biosynthesis | BVRB_2g034560 | 104886661 | XM_010671128.2 | SHT | spermidine hydroxycinnamoyl transferase | root | 1.924698634 | 0.003698958 |
| bvg00073 | Cutin, suberine and wax biosynthesis | BVRB_7g172510 | 104899987 | XM_010687294.1 | CYP86A1 | cytochrome P450 86A1 | root | 1.863155593 | 0.000137696 |

**Supplementary Table 5. DEGs involved in photosynthesis in sugar beet under LN stress**

| **Transcript_id** | **Gene_id** | **Locus_tag** | **Gene name** | **Gene_description** | **Log2(fold change)** | **qvalue** | **Accession** | **Term name** | **Domain** |
| --- | --- | --- | --- | --- | --- | --- | --- | --- | --- |
| XM_010688632.2 | 104901086 | BVRB_8g186680 | PsbQ | oxygen-evolving enhancer protein 3, chloroplastic(LOC104901086) | -1.918021215 | 3.96682E-06 | GO:0015979, GO:0009521, GO:0034357, GO:0009523 | photosynthesis, photosystem, photosynthetic membrane, photosystem II | BP, CC |
| XM_010682848.2 | 104896140 | BVRB_6g139980 | PsaD | photosystem I reaction center subunit II, chloroplastic(LOC104896140) | -1.01040217 | 0.014381732 | GO:0015979, GO:0009521, GO:0034357, GO:0009522, GO:0009538 | photosynthesis, photosystem, photosynthetic membrane, photosystem I, photosystem I reaction center | BP, CC |
| XM_010687366.2 | 104900039 | BVRB_7g173090 | Psb27-1 | photosystem II repair protein PSB27-H1, chloroplastic(LOC104900039) | -1.681681311 | 0.001068486 | GO:0015979，GO:0009523 | photosynthesis，photosystem II | BP，CC |
| XM_010684840.2 | 104897878 | BVRB_6g153560 | PNSL2 | photosynthetic NDH subunit of lumenal location 2, chloroplastic(LOC104897878) | -2.207254721 | 1.992E-06 | GO:0015979, GO:0009521, GO:0034357, GO:0009523 | photosynthesis, photosystem, photosynthetic membrane, photosystem II | BP, CC |
| XM_010696954.2 | 104907924 | BVRB_5g123870 | PsbP1 | psbP-like protein 1, chloroplastic(LOC104907924) | -1.205931821 | 0.01067037 | GO:0015979, GO:0009521, GO:0034357, GO:0009523 | photosynthesis, photosystem, photosynthetic membrane, photosystem II | BP, CC |
| XM_010697661.2 | 104908544 | BVRB_7g176840 | PsbY | photosystem II core complex proteins psbY, chloroplastic(LOC104908544) | -1.611778051 | 0.010347779 | GO:0015979, GO:0009521, GO:0034357, GO:0009523 | photosynthesis, photosystem, photosynthetic membrane, photosystem II | BP, CC |
| XM_010681842.2 | 104895363 | BVRB_6g132120 | PsbP | oxygen-evolving enhancer protein 2, chloroplastic(LOC104895363) | -1.439531184 | 0.000387634 | GO:0015979, GO:0009521, GO:0034357, GO:0009523 | photosynthesis, photosystem, photosynthetic membrane, photosystem II | BP, CC |
| XM_010675628.2 | 104890225 | BVRB_4g075220 | PsaL | photosystem I reaction center subunit XI, chloroplastic(LOC104890225) | -1.255512797 | 0.002969247 | GO:0015979, GO:0009521, GO:0034357, GO:0009522, GO:0009538 | photosynthesis, photosystem, photosynthetic membrane, photosystem I, photosystem I reaction center | BP, CC |
| XM_010693035.2 | 104904686 | BVRB_9g221000 | PsaH | photosystem I reaction center subunit VI, chloroplastic(LOC104904686) | -1.881740529 | 6.4371E-06 | GO:0015979, GO:0009521, GO:0034357, GO:0009522, GO:0009538 | photosynthesis, photosystem, photosynthetic membrane, photosystem I, photosystem I reaction center | BP, CC |
| XM_019251428.1 | 104902061 | BVRB_8g194720 | - | F-box/kelch-repeat protein At1g55270(LOC104902061) | -11.49108036 | 0.003121075 | GO:0015979, GO:0009521, GO:0034357, GO:0009522 | photosynthesis, photosystem, photosynthetic membrane, photosystem I | BP, CC |
| XM_010676256.2 | 104890679 | BVRB_4g080040 | PPD1 | psbP domain-containing protein 1, chloroplastic(LOC104890679) | -1.395309121 | 0.035225451 | GO:0015979, GO:0009521, GO:0034357, GO:0009523 | photosynthesis, photosystem, photosynthetic membrane, photosystem II | BP, CC |
| XM_010676417.1 | 104890818 | BVRB_4g081180 | PPD7 | psbP domain-containing protein 7, chloroplastic(LOC104890818) | -2.044901766 | 0.000847763 | GO:0015979, GO:0009521, GO:0034357, GO:0009523 | photosynthesis, photosystem, photosynthetic membrane, photosystem II | BP, CC |
| XM_010696550.2 | 104907596 | BVRB_4g096020 | PsbR | photosystem II 10 kDa polypeptide, chloroplastic(LOC104907596) | -0.864035331 | 0.048936432 | GO:0015979, GO:0009521, GO:0034357, GO:0009523 | photosynthesis, photosystem, photosynthetic membrane, photosystem II | BP, CC |
| XM_010688303.2 | 104900783 | BVRB_1g013940 | PPD3 | psbP domain-containing protein 3, chloroplastic(LOC104900783) | -1.622704646 | 0.003810407 | GO:0015979, GO:0009521, GO:0034357, GO:0009523 | photosynthesis, photosystem, photosynthetic membrane, photosystem II | BP, CC |
| XM_010681903.2 | 104895406 | BVRB_6g132600 | PsaN | photosystem I reaction center subunit N, chloroplastic(LOC104895406) | -2.028164994 | 3.37995E-06 | GO:0015979, GO:0009521, GO:0034357, GO:0009522, GO:0009523 | photosynthesis, photosystem, photosynthetic membrane, photosystem I, photosystem II | BP, CC |
| XM_010695967.2 | 104907092 | BVRB_4g092210 | PNSL3 | photosynthetic NDH subunit of lumenal location 3, chloroplastic(LOC104907092) | -1.498531703 | 0.006119296 | GO:0015979, GO:0009521, GO:0034357, GO:0009523 | photosynthesis, photosystem, photosynthetic membrane, photosystem II | BP, CC |
| XM_010684065.2 | 104897231 | BVRB_6g147470 | PsbY | photosystem II core complex proteins psbY, chloroplastic(LOC104897231) | -1.11500069 | 0.008430948 | GO:0015979, GO:0009521, GO:0034357, GO:0009523 | photosynthesis, photosystem, photosynthetic membrane, photosystem II | BP, CC |
| XM_010692092.2 | 104903946 | BVRB_9g214300 | PsaF | photosystem I reaction center subunit III, chloroplastic(LOC104903946) | -1.260108654 | 0.001992395 | GO:0015979, GO:0009521, GO:0034357, GO:0009522, GO:0009538 | photosynthesis, photosystem, photosynthetic membrane, photosystem I, photosystem I reaction center | BP, CC |
| XM_010675295.2 | 104889953 | BVRB_4g072250 | PsbO | oxygen-evolving enhancer protein 1, chloroplastic(LOC104889953) | -1.475772583 | 0.000319495 | GO:0015979, GO:0009521, GO:0034357, GO:0009523 | photosynthesis, photosystem, photosynthetic membrane, photosystem II | BP, CC |
| XM_010687791.2 | 104900376 | BVRB_7g176760 | PsaK | photosystem I reaction center subunit psaK, chloroplastic(LOC104900376) | -2.000230128 | 7.82005E-07 | GO:0015979, GO:0009521, GO:0034357, GO:0009522 | photosynthesis, photosystem, photosynthetic membrane, photosystem I | BP, CC |
| XM_010686519.2 | 104899345 | BVRB_7g166490 | PsaE | photosystem I reaction center subunit IV, chloroplastic(LOC104899345) | -1.362812702 | 0.000823394 | GO:0015979, GO:0009521, GO:0034357, GO:0009522, GO:0009538 | photosynthesis, photosystem, photosynthetic membrane, photosystem I, photosystem I reaction center | BP, CC |
| XM_010680869.2 | 104894597 | BVRB_5g120370 | PsbW | photosystem II reaction center W protein, chloroplastic(LOC104894597) | -1.048190581 | 0.013972968 | GO:0015979, GO:0009521, GO:0034357, GO:0009523 | photosynthesis, photosystem, photosynthetic membrane, photosystem II | BP, CC |
| XM_010691011.2 | 104903038 | BVRB_9g206750 | PetC | cytochrome b6-f complex iron-sulfur subunit, chloroplastic(LOC104903038) | -0.975502878 | 0.031802427 | GO:0034357 | photosynthetic membrane | CC |
| XM_010678957.2 | 104892934 | BVRB_5g106170 | PetF | ferredoxin C 2, chloroplastic(LOC104892934) | -1.307309975 | 0.018241319 | bvg00195 | Photosynthesis | - |
| XM_010688536.2 | 104900994 | BVRB_8g186220 | ATPC | ATP synthase gamma chain, chloroplastic(LOC104900994) | -1.057399927 | 0.014676094 | bvg00195 | Photosynthesis | - |
| XM_010668437.2 | 104883872 | BVRB_004160 | PetE | plastocyanin, chloroplastic(LOC104883872) | -2.268497734 | 2.5794E-07 | bvg00195 | Photosynthesis | - |
| XM_010684398.2 | 104897513 | BVRB_6g150240 | ATPD | ATP synthase delta chain, chloroplastic(LOC104897513) | -1.058966361 | 0.012658719 | bvg00195 | Photosynthesis | - |
| XM_010696688.2 | 104907718 | BVRB_4g096780 | PsaO | photosystem I subunit O(LOC104907718) | -2.313533697 | 2.35749E-07 | bvg00195 | Photosynthesis | - |
| XM_010689939.2 | 104902235 | BVRB_8g196730 | PetF | ferredoxin-2(LOC104902235) | -1.425684551 | 0.034938952 | bvg00195 | Photosynthesis | - |
| XM_010675082.2 | 104889776 | BVRB_3g065490 | PsaG | photosystem I reaction center subunit V, chloroplastic(LOC104889776) | -1.504537936 | 0.000535619 | bvg00195 | Photosynthesis | - |
| XM_010687703.2 | 104900312 | BVRB_7g176080 | PetF | ferredoxin, root R-B1(LOC104900312) | -2.857279323 | 9.56277E-08 | bvg00195 | Photosynthesis | - |
| XM_010693637.2 | 104905186 | BVRB_1g016820 | PetH | ferredoxin--NADP reductase, root isozyme, chloroplastic(LOC104905186) | -2.018916264 | 1.01479E-05 | bvg00195 | Photosynthesis | - |
| XM_010670785.2 | 104886325 | BVRB_2g033010 | Lhca2 | chlorophyll a-b binding protein, chloroplastic(LOC104886325) | -1.319103436 | 0.001720824 | bvg00196 | Photosynthesis - antenna proteins | - |
| XM_010687591.2 | 104900217 | BVRB_7g175110 | Lhcb1 | chlorophyll a-b binding protein, chloroplastic(LOC104900217) | -2.142243834 | 2.62207E-05 | bvg00196 | Photosynthesis - antenna proteins | - |
| XM_010689244.2 | 104901652 | BVRB_8g190730 | Lhca2 | photosystem I chlorophyll a/b-binding protein 6, chloroplastic(LOC104901652) | -2.065337477 | 1.97107E-05 | bvg00196 | Photosynthesis - antenna proteins | - |
| XM_010672415.2 | 104887706 | BVRB_2g043310 | Lhcb4.1 | chlorophyll a-b binding protein CP29.1, chloroplastic(LOC104887706) | -2.447258004 | 1.1653E-07 | bvg00196 | Photosynthesis - antenna proteins | - |
| XM_010667637.2 | 104883168 | BVRB_9g225010 | Lhca5 | chlorophyll a-b binding protein 5, chloroplastic(LOC104883168) | -3.841754015 | 2.70894E-12 | bvg00196 | Photosynthesis - antenna proteins | - |
| XM_019247425.1 | 104883672 | BVRB_002570 | - | uncharacterized LOC104883672(LOC104883672) | -4.21382732 | 2.39774E-09 | bvg00196 | Photosynthesis - antenna proteins | - |
| XM_010696126.2 | 104907232 | BVRB_4g093140 | Lhcb3 | chlorophyll a-b binding protein 13, chloroplastic(LOC104907232) | -2.530766342 | 3.79332E-07 | bvg00196 | Photosynthesis - antenna proteins | - |
| XM_010677488.2 | 104891741 | BVRB_4g089040 | Lhca5 | photosystem I chlorophyll a/b-binding protein 5, chloroplastic(LOC104891741) | -1.080308099 | 0.038832796 | bvg00196 | Photosynthesis - antenna proteins | - |
| XM_010668563.2 | 104883981 | BVRB_005040 | Lhcb6 | chlorophyll a-b binding protein CP24, chloroplastic(LOC104883981) | -2.519912263 | 8.34141E-08 | bvg00196 | Photosynthesis - antenna proteins | - |
| XM_010678866.2 | 104892849 | BVRB_5g105530 | Lhcb4.3 | chlorophyll a-b binding protein CP29.3, chloroplastic(LOC104892849) | -1.433094913 | 0.000701611 | bvg00196 | Photosynthesis - antenna proteins | - |
| XM_010694241.2 | 104905645 | BVRB_1g021010 | Lhca3 | photosystem I chlorophyll a/b-binding protein 3-1, chloroplastic(LOC104905645) | -1.435368167 | 0.000905952 | bvg00196 | Photosynthesis - antenna proteins | - |
| XM_010680190.2 | 104894032 | BVRB_5g114540 | Lhca4 | chlorophyll a-b binding protein 4, chloroplastic(LOC104894032) | -2.710969332 | 1.93053E-09 | bvg00196 | Photosynthesis - antenna proteins | - |
| XM_010671568.2 | 104886989 | BVRB_1g004060 | Lhcb5 | chlorophyll a-b binding protein CP26, chloroplastic(LOC104886989) | -2.294554714 | 1.01183E-07 | bvg00196 | Photosynthesis - antenna proteins | - |
| XM_010695090.2 | 104906349 | - | Lhca1 | chlorophyll a-b binding protein 6, chloroplastic(LOC104906349) | -1.986896531 | 2.02343E-05 | bvg00196 | Photosynthesis - antenna proteins | - |
| XM_010667637.2 | 104883168 | BVRB_9g225010 | Lhcb2 | chlorophyll a-b binding protein 5, chloroplastic(LOC104883168) | -3.841754015 | 2.70894E-12 | bvg00196 | Photosynthesis - antenna proteins | - |

**Supplementary Table 6. DEGs associated with C, N and glutathione metabolism biosynthetic pathways under LN stress in sugar beet**

| **Transcript_id** | **Gene_id** | **Locus_tag** | **Gene name** | **Gene_description** | **Log2(fold change)** | **pvalue** | **qvalue** |
| --- | --- | --- | --- | --- | --- | --- | --- |
|  |  |  |  | **Carbon fixation in photosynthetic organisms** |  |  |  |
| XM_010671692.2 | 104887103 | BVRB_2g038200 | PGK | phosphoglycerate kinase, chloroplastic (LOC104887103) | -1.091270835 | 7.28E-05 | 0.008547566 |
| XM_010677812.2 | 104892005 | BVRB_4g091040 | FBA5 | fructose-bisphosphate aldolase 5, cytosolic (LOC104892005) | -2.092385717 | 8.00E-10 | 5.76E-07 |
| XM_010673689.2 | 104888662 | BVRB_3g055740 | TKL | transketolase, chloroplastic (LOC104888662) | -0.895715548 | 0.0004387 | 0.033231753 |
| XM_010694771.2 | 104906059 | BVRB_2g026840 | RBCS | ribulose bisphosphate carboxylase small subunit, chloroplastic 1 (LOC104906059) | -1.427403088 | 5.22E-06 | 0.001092457 |
| XM_010671527.2 | 104886961 | BVRB_2g037220 | RBCS | ribulose bisphosphate carboxylase small subunit, chloroplastic 6 (LOC104886961) | -2.232709632 | 1.16E-09 | 8.01E-07 |
| XM_010694752.2 | 104906047 | BVRB_2g026810 | RBCS | ribulose bisphosphate carboxylase small subunit, chloroplastic 1 (LOC104906047) | -3.713809016 | 2.34E-14 | 6.05E-11 |
| XM_010680176.2 | 104894019 | BVRB_5g114350 | RPE | ribulose-phosphate 3-epimerase, chloroplastic (LOC104894019) | -0.890067386 | 0.000667401 | 0.04490621 |
| XM_010694757.2 | 104906053 | BVRB_2g026850 | RBCS | ribulose bisphosphate carboxylase small subunit, chloroplastic 1 (LOC104906053) | -1.7831231 | 5.22E-08 | 2.25E-05 |
| XM_010694797.2 | 104906081 | BVRB_2g026820 | RBCS | ribulose bisphosphate carboxylase small subunit, chloroplastic 1 (LOC104906081) | -1.957118769 | 6.74E-09 | 3.74E-06 |
| XM_010692461.2 | 104904258 | BVRB_9g216490 | FBA1 | fructose-bisphosphate aldolase 1, chloroplastic (LOC104904258) | -1.383071338 | 6.94E-06 | 0.001354362 |
| XM_010688554.2 | 104901009 | BVRB_1g014260 | GAPB | glyceraldehyde-3-phosphate dehydrogenase B, chloroplastic (LOC104901009) | -1.143958692 | 0.00012686 | 0.013224081 |
| XM_010696330.2 | 104907408 | BVRB_4g094590 | GAPA | glyceraldehyde-3-phosphate dehydrogenase A, chloroplastic (LOC104907408) | -1.20840357 | 3.48E-05 | 0.00493518 |
| XM_010682320.2 | 104895737 | BVRB_1g011460 | FBP | fructose-1,6-bisphosphatase, cytosolic (LOC104895737) | -1.236729707 | 2.66E-05 | 0.00403044 |
| XM_010679762.2 | 104893633 | BVRB_5g111310 | FBP | fructose-1,6-bisphosphatase, chloroplastic (LOC104893633) | -1.004230566 | 0.000212178 | 0.019371139 |
| XM_010684124.2 | 104897276 | BVRB_2g026810 | GGAT2 | glutamate--glyoxylate aminotransferase 2 (LOC104897276) | -1.098754975 | 0.000143794 | 0.014533805 |
|  |  |  |  | **Carbon metabolism** |  |  |  |
| XM_010694797.2 | 104906081 | BVRB_2g026820 | RBCS | ribulose bisphosphate carboxylase small subunit, chloroplastic 1 (LOC104906081) | -1.957118769 | 6.74E-09 | 3.74E-06 |
| XM_010680010.2 | 104893881 | BVRB_5g112980 | PDH-E1 BETA | pyruvate dehydrogenase E1 component subunit beta-1, mitochondrial (LOC104893881) | -1.398211624 | 1.03E-05 | 0.001896827 |
| XM_010681253.2 | 104894893 | BVRB_6g127540 | AMT | aminomethyltransferase, mitochondrial (LOC104894893) | -1.337056704 | 9.33E-06 | 0.001757153 |
| XM_010673746.2 | 104888710 | BVRB_3g056180 | GLUDB | glutamate dehydrogenase B (LOC104888710) | -1.026251078 | 0.000520918 | 0.037662469 |
| XM_010694771.2 | 104906059 | BVRB_2g026840 | RBCS | ribulose bisphosphate carboxylase small subunit, chloroplastic 1 (LOC104906059) | -1.427403088 | 5.22E-06 | 0.001092457 |
| XM_010694757.2 | 104906053 | BVRB_2g026850 | RBCS | ribulose bisphosphate carboxylase small subunit, chloroplastic 1 (LOC104906053) | -1.7831231 | 5.22E-08 | 2.25E-05 |
| XM_010684124.2 | 104897276 | BVRB_2g026820 | GGAT2 | glutamate--glyoxylate aminotransferase 2 (LOC104897276) | -1.098754975 | 0.000143794 | 0.014533805 |
| XM_010671692.2 | 104887103 | BVRB_2g038200 | PGK | phosphoglycerate kinase, chloroplastic (LOC104887103) | -1.091270835 | 7.28E-05 | 0.008547566 |
| XM_010679762.2 | 104893633 | BVRB_5g111310 | FBP | fructose-1,6-bisphosphatase, chloroplastic (LOC104893633) | -1.004230566 | 0.000212178 | 0.019371139 |
| XM_010673689.2 | 104888662 | BVRB_3g055740 | TKL | transketolase, chloroplastic (LOC104888662) | -0.895715548 | 0.0004387 | 0.033231753 |
| XM_010675576.2 | 104890185 | BVRB_2g026850 | PGLP1A | phosphoglycolate phosphatase 1A, chloroplastic (LOC104890185) | -1.126296662 | 0.000138011 | 0.014092018 |
| XM_010680176.2 | 104894019 | BVRB_5g114350 | RPE | ribulose-phosphate 3-epimerase, chloroplastic (LOC104894019) | -0.890067386 | 0.000667401 | 0.04490621 |
| XM_010688554.2 | 104901009 | BVRB_1g014260 | GAPB | glyceraldehyde-3-phosphate dehydrogenase B, chloroplastic (LOC104901009) | -1.143958692 | 0.00012686 | 0.013224081 |
| XM_010696330.2 | 104907408 | BVRB_4g094590 | GAPA | glyceraldehyde-3-phosphate dehydrogenase A, chloroplastic (LOC104907408) | -1.20840357 | 3.48E-05 | 0.00493518 |
| XM_010687072.2 | 104899804 | BVRB_7g170530 | GLYR2 | glyoxylate/succinic semialdehyde reductase 2, chloroplastic (LOC104899804) | -1.383781158 | 0.000565353 | 0.039941462 |
| XM_010682320.2 | 104895737 | BVRB_1g011460 | FBP | fructose-1,6-bisphosphatase, cytosolic (LOC104895737) | -1.236729707 | 2.66E-05 | 0.00403044 |
| XM_010677812.2 | 104892005 | BVRB_4g091040 | FBA5 | fructose-bisphosphate aldolase 5, cytosolic (LOC104892005) | -2.092385717 | 8.00E-10 | 5.76E-07 |
| XM_010671527.2 | 104886961 | BVRB_2g037220 | RBCS | ribulose bisphosphate carboxylase small subunit, chloroplastic 6 (LOC104886961) | -2.232709632 | 1.16E-09 | 8.01E-07 |
| XM_010694752.2 | 104906047 | BVRB_2g026810 | RBCS | ribulose bisphosphate carboxylase small subunit, chloroplastic 1 (LOC104906047) | -3.713809016 | 2.34E-14 | 6.05E-11 |
| XM_010692461.2 | 104904258 | BVRB_9g216490 | FBA1 | fructose-bisphosphate aldolase 1, chloroplastic (LOC104904258) | -1.383071338 | 6.94E-06 | 0.001354362 |
| XM_010684411.2 | 104897526 | BVRB_6g150380 | G6PD | glucose-6-phosphate 1-dehydrogenase, chloroplastic (LOC104897526) | -1.705549302 | 1.18E-05 | 0.002102965 |
| XM_010667582.2 | 104883129 | BVRB_9g224670 | HK1 | hexokinase-1 (LOC104883129) | -2.003698514 | 2.31E-06 | 0.000558315 |
|  |  |  |  | **Nitrogen metabolism** |  |  |  |
| XM_010697516.2 | 104908396 | BVRB_6g155520 | NRT2.5 | high affinity nitrate transporter 2.5 (LOC104908396) | -3.100873426 | 3.24E-07 | 0.000103743 |
| XM_010689694.1 | 104902037 | BVRB_8g194450 | CA | carbonic anhydrase, chloroplastic (LOC104902037) | -2.357037799 | 1.68E-11 | 1.96E-08 |
| XM_010672627.2 | 104887841 | BVRB_1g000370 | NR | nitrate reductase [NADH] (LOC104887841) | -3.336381512 | 2.59E-17 | 1.01E-13 |
| XM_010668031.2 | 104883503 | BVRB_001510 | GS | glutamine synthetase leaf isozyme, chloroplastic (LOC104883503) | -2.316273237 | 2.55E-12 | 1.68E-09 |
| XM_010677274.2 | 104891570 | BVRB_4g087070 | NIR | ferredoxin--nitrite reductase, chloroplastic (LOC104891570) | -2.098912269 | 4.12E-09 | 2.44E-06 |
| XM_010673746.2 | 104888710 | BVRB_3g056180 | GLUDB | glutamate dehydrogenase B (LOC104888710) | -1.026251078 | 0.000520918 | 0.037662469 |
| XM_010698005.2 | 104908841 | BVRB_7g179180 | CA4 | alpha carbonic anhydrase 4 (LOC104908841) | 2.438524533 | 1.10934E-09 | 3.79798E-07 |
| XM_010673169.2 | 104888263 | BVRB_3g052140 | GS1 | glutamate synthase 1 [NADH], chloroplastic (LOC104888263) | -1.207821297 | 0.000152854 | 0.009464186 |
| XM_010685423.2 | 104898358 | BVRB_7g159420 | CA1 | alpha carbonic anhydrase 1, chloroplastic (LOC104898358) | 1.655145361 | 0.001075013 | 0.042732538 |
| XM_010684253.2 | 104897387 | BVRB_6g148840 | CA2 | carbonic anhydrase 2 (LOC104897387) | -2.33836995 | 8.83967E-05 | 0.006179981 |
| XM_010685598.2 | 104898511 | BVRB_7g160540 | NRT2.4 | high affinity nitrate transporter 2.4 (LOC104898511) | #NAME? | 0.000535425 | 0.024896899 |
| XM_010698003.2 | 104908840 | BVRB_7g179170 | CA7 | alpha carbonic anhydrase 7 (LOC104908840) | 3.337399301 | 7.16617E-12 | 4.17083E-09 |
|  |  |  |  | **Glutathione metabolism** |  |  |  |
| XM_019250092.1 | 104895313 | BVRB_6g130380 | APX3 | L-ascorbate peroxidase 3 (LOC104895313) | 2.032000064 | 2.86382E-06 | 0.000378999 |
| XM_010696278.2 | 104907361 | BVRB_4g094330 | PARC | probable glutathione S-transferase parC (LOC104907361) | 1.971690208 | 4.81428E-05 | 0.003825243 |
| XM_010669253.2 | 104884575 | BVRB_009580 | GST23 | glutathione transferase GST 23 (LOC104884575) | 2.164612736 | 7.67239E-08 | 1.66933E-05 |
| XM_010684996.2 | 104898005 | BVRB_6g154910 | GSTU17 | glutathione S-transferase U17 (LOC104898005) | 1.759896539 | 7.79822E-06 | 0.000887041 |
| XM_010680230.2 | 104894062 | BVRB_5g114880 | GSTU9 | glutathione S-transferase U9 (LOC104894062) | 4.227492005 | 3.73159E-06 | 0.00047472 |
| XM_010680227.2 | 104894059 | BVRB_5g114850 | GSTU9 | glutathione S-transferase U9 (LOC104894059) | 1.818712291 | 0.000148266 | 0.009253946 |
| XM_010680225.2 | 104894057 | BVRB_5g114830 | GSTU9 | glutathione S-transferase U9 (LOC104894057) | 2.13626471 | 1.20357E-06 | 0.000181554 |
| XM_010690091.2 | 104902346 | BVRB_8g197940 | GGT3 | glutathione hydrolase 3 (LOC104902346) | 1.978308034 | 2.40611E-05 | 0.002185778 |
| XM_010687356.2 | 104900033 | BVRB_7g173050 | GSTU7 | glutathione S-transferase U7 (LOC104900033) | 4.265344388 | 1.8591E-08 | 4.70362E-06 |
| XM_010696642.2 | 104907677 | BVRB_4g096480 | PARC | probable glutathione S-transferase parC (LOC104907677) | 1.700532001 | 0.000279497 | 0.015120528 |
| XM_010676654.2 | 104891011 | BVRB_1g007470 | APX6 | probable L-ascorbate peroxidase 6, chloroplastic/mitochondrial (LOC104891011) | -0.993935868 | 0.000554217 | 0.039457345 |
| XM_010677596.2 | 104891829 | BVRB_1g008510 | TL29 | thylakoid lumenal 29 kDa protein, chloroplastic (LOC104891829) | -2.333612453 | 3.84749E-09 | 2.31652E-06 |
| XM_010684411.2 | 104897526 | BVRB_6g150380 | G6PD | glucose-6-phosphate 1-dehydrogenase, chloroplastic (LOC104897526) | -1.705549302 | 1.18334E-05 | 0.002102965 |

**Supplementary Table 7. DEGs associated with secondary metabolism under LN stress in sugar beet**

| **Transcript_id** | **Gene_id** | **Locus_tag** | **Gene name** | **Gene_description** | **Log2(fold change)** | **p value** | **q value** |
| --- | --- | --- | --- | --- | --- | --- | --- |
|  |  |  |  | **Phenylpropanoid biosynthesis** |  |  |  |
| XM_019250993.1 | 104900440 | BVRB_7g172630 | BACOVA_02659 | beta-glucosidase BoGH3B (LOC104900440) | -4.532419465 | 0.001289974 | 0.049071015 |
| XM_010672817.2 | 104888010 | BVRB_3g049860 | 4CL2 | 4-coumarate--CoA ligase 2 (LOC104888010) | 1.667267813 | 3.9304E-07 | 7.16728E-05 |
| XM_010675264.2 | 104889925 | - | BGLU13 | beta-glucosidase 13 (LOC104889925) | 3.86371059 | 0.000311892 | 0.01639064 |
| XM_010675265.2 | 104889926 | - | - | - | 2.01597039 | 0.000257211 | 0.014212114 |
| XM_019252566.1 | 104908274 | BVRB_2g029820 | PER7 | peroxidase P7 (LOC104908274) | 3.881413581 | 2.34628E-05 | 0.002144877 |
| XM_010685248.2 | 104898205 | BVRB_7g157590 | PNC1 | cationic peroxidase 1 (LOC104898205) | 2.747519058 | 2.18241E-09 | 6.92835E-07 |
| XM_010688539.2 | 104900996 | BVRB_8g186240 | PER42 | peroxidase 42 (LOC104900996) | 1.230041583 | 0.000348254 | 0.017775177 |
| XM_010668612.2 | 104884022 | BVRB_005400 | CAD6 | probable cinnamyl alcohol dehydrogenase 6 (LOC104884022) | 2.343318013 | 9.12805E-06 | 0.00100397 |
| XM_010676222.2 | 104890653 | BVRB_4g079730 | PER5 | peroxidase 5 (LOC104890653) | 3.180036258 | 1.38721E-14 | 1.3272E-11 |
| XM_010677311.2 | 104891604 | BVRB_4g087470 | BGLU18 | beta-glucosidase 18 (LOC104891604) | 1.669251582 | 0.001171978 | 0.045919478 |
| XM_010697429.2 | 104908331 | BVRB_2g029890 | PER7 | peroxidase P7 (LOC104908331) | 2.713934908 | 2.42632E-05 | 0.002192223 |
| XM_010679602.2 | 104893499 | BVRB_5g110540 | BGLU13 | beta-glucosidase 13 (LOC104893499) | 2.121317093 | 7.84872E-08 | 1.70239E-05 |
| XM_010678992.2 | 104892966 | BVRB_5g106570 | PER20 | peroxidase 20 (LOC104892966) | 2.06879058 | 1.29677E-08 | 3.37943E-06 |
| XM_010687685.2 | 104900294 | BVRB_7g175860 | PER57 | peroxidase 57 (LOC104900294) | 3.232860417 | 7.59359E-06 | 0.000868037 |
| XM_010687686.2 | 104900295 | BVRB_7g175870 | PER57 | peroxidase 57 (LOC104900295) | 2.511889917 | 3.80273E-06 | 0.000482015 |
| XM_010667756.2 | 104883267 | BVRB_9g225600 | SAT | stemmadenine O-acetyltransferase (LOC104883267) | -1.374236569 | 0.001072738 | 0.042666373 |
| XM_010691417.2 | 104903392 | BVRB_9g208880 | PER4 | peroxidase 4 (LOC104903392) | -2.235713175 | 0.000502692 | 0.023883666 |
| XM_010697796.1 | 104908662 | BVRB_7g177530 | PER57 | peroxidase 57 (LOC104908662) | -1.579802823 | 0.000119024 | 0.007864571 |
| XM_010671128.2 | 104886661 | BVRB_2g034560 | SHT | spermidine hydroxycinnamoyl transferase (LOC104886661) | 1.924698634 | 4.62026E-05 | 0.003698958 |
| XM_010697362.2 | 104908267 | BVRB_2g029810 | PER7 | peroxidase P7 (LOC104908267) | 2.894408154 | 1.71328E-07 | 3.42826E-05 |
| XM_010685251.2 | 104898208 | BVRB_7g157620 | PNC1 | cationic peroxidase 1 (LOC104898208) | 2.478310182 | 8.86303E-07 | 0.000142302 |
| XM_010673015.2 | 104888141 | - | PER5 | peroxidase 5-like (LOC104888141) | 1.337277475 | 0.000530067 | 0.024730071 |
| XM_010674488.2 | 104889293 | BVRB_3g061430 | HST | shikimate O-hydroxycinnamoyl transferase (LOC104889293) | 1.560720316 | 0.0009408 | 0.038674144 |
| XM_010667245.2 | 104882831 | BVRB_8g200320 | BGLU46 | beta-glucosidase 46 (LOC104882831) | 3.869491052 | 0.000141138 | 0.008920693 |
| XM_010673030.2 | 104888149 | BVRB_3g050950 | COMT | caffeic acid 3-O-methyltransferase (LOC104888149) | 1.216830354 | 0.000414006 | 0.020291221 |
| XM_010689693.2 | 104902035 | BVRB_8g194440 | PER25 | peroxidase 25 (LOC104902035) | -1.183409811 | 0.000115345 | 0.007657722 |
| XM_010672763.2 | 104887972 | BVRB_3g049400 | COMT | caffeic acid 3-O-methyltransferase (LOC104887972) | 2.049425659 | 3.47534E-05 | 0.002949269 |
| XM_010683011.2 | 104896291 | - | PER11 | peroxidase 11 (LOC104896291) | 1.823996703 | 7.51738E-08 | 1.64586E-05 |
| XM_010671086.2 | 104886619 | BVRB_2g034550 | SHT | spermidine hydroxycinnamoyl transferase (LOC104886619) | 1.578271848 | 6.4045E-05 | 0.004788236 |
| XM_010698104.2 | 104908933 | BVRB_7g179870 | PER21 | peroxidase 21 (LOC104908933) | 1.821748545 | 1.13032E-06 | 0.000173886 |
|  |  |  |  | **Flavonoid biosynthesis** |  |  |  |
| XM_010671488.2 | 104886934 | BVRB_2g036940 | CHI | chalcone--flavanone isomerase (LOC104886934) | 2.576304597 | 4.61777E-11 | 2.22423E-08 |
| XM_010674949.2 | 104889670 | BVRB_1g007170 | DFRA | dihydroflavonol 4-reductase (LOC104889670) | 3.89551494 | 0.000275174 | 0.01495618 |
| XM_010671086.2 | 104886619 | BVRB_2g034550 | SHT | spermidine hydroxycinnamoyl transferase (LOC104886619) | 1.578271848 | 6.4045E-05 | 0.004788236 |
| XM_010681635.2 | 104895196 | - | F3H | naringenin,2-oxoglutarate 3-dioxygenase (LOC104895196) | 2.426221321 | 3.4617E-06 | 0.000448041 |
| XM_010671128.2 | 104886661 | BVRB_2g034560 | SHT | spermidine hydroxycinnamoyl transferase (LOC104886661) | 1.924698634 | 4.62026E-05 | 0.003698958 |
| XM_010693891.2 | 104905365 | BVRB_1g018740 | CHS3 | chalcone synthase 3 (LOC104905365) | 7.478293343 | 8.07373E-35 | 5.63885E-31 |
| XM_010667756.2 | 104883267 | BVRB_9g225600 | SAT | stemmadenine O-acetyltransferase (LOC104883267) | -1.374236569 | 0.001072738 | 0.042666373 |
| XM_010693893.2 | 104905367 | BVRB_1g018770 | CHS | chalcone synthase (LOC104905367) | 3.102508381 | 2.62784E-13 | 2.08561E-10 |
| XM_010693892.2 | 104905366 | BVRB_1g018750 | CHS3 | chalcone synthase 3(LOC104905366) | 6.548582197 | 3.85753E-24 | 1.17138E-20 |
| XM_010674488.2 | 104889293 | BVRB_3g061430 | HST | shikimate O-hydroxycinnamoyltransferase (LOC104889293) | 1.560720316 | 0.0009408 | 0.038674144 |
| XM_010672304.2 | 104887630 | BVRB_2g042480 | CHS | chalcone synthase (LOC104887630) | -2.24001417 | 0.000348718 | 0.017777495 |
| XM_010671471.1 | 104886918 | BVRB_2g036880 | CHI | chalcone--flavanone isomerase (LOC104886918) | 2.746736996 | 3.85324E-07 | 7.08205E-05 |
| XM_010667546.2 | 104883093 | BVRB_8g202170 | FLS1 | flavonol synthase/flavanone 3-hydroxylase (LOC104883093) | 1.918033757 | 2.29143E-06 | 0.000309575 |
|  |  |  |  | **Biosynthesis of secondary metabolites** |  |  |  |
| XM_019250993.1 | 104900440 | BVRB_7g172630 | BACOVA_02659 | beta-glucosidase BoGH3B (LOC104900440) | -4.532419465 | 0.001289974 | 0.049071015 |
| XM_010681635.2 | 104895196 | - | F3H | naringenin,2-oxoglutarate 3-dioxygenase | 2.426221321 | 3.4617E-06 | 0.000448041 |
| XM_010684858.2 | 104897892 | BVRB_6g153680 | KCS11 | 3-ketoacyl-CoA synthase 11 (LOC104897892) | 3.646481875 | 9.3548E-16 | 1.14624E-12 |
| XM_010689299.2 | 104901700 | BVRB_8g191190 | AASS | alpha-aminoadipic semialdehyde synthase (LOC104901700) | 1.370283767 | 0.000147018 | 0.009217254 |
| XM_010695618.2 | 104906803 | BVRB_3g069300 | IPT5 | adenylate isopentenyltransferase 5, chloroplastic (LOC104906803) | -3.332743449 | 8.50607E-13 | 6.18834E-10 |
| XM_010692662.2 | 104904415 | BVRB_2g025190 | GPAT7 | glycerol-3-phosphate acyltransferase 7 (LOC104904415) | 1.886743078 | 1.8655E-08 | 4.70362E-06 |
| XM_010692359.2 | 104904140 | BVRB_1g002310 | KCS1 | 3-ketoacyl-CoA synthase 1 (LOC104904140) | 2.122684688 | 6.21135E-07 | 0.000106851 |
| XM_010688265.2 | 104900761 | BVRB_8g183870 | PPO | polyphenol oxidase, chloroplastic (LOC104900761) | -1.409205488 | 1.00107E-05 | 0.001078611 |
| XM_019250155.1 | 104895517 | BVRB_6g133820 | OPR3 | 12-oxophytodienoate reductase 3 (LOC104895517) | Inf | 0.000408748 | 0.020116142 |
| XM_010672304.2 | 104887630 | BVRB_2g042480 | CHS | chalcone synthase (LOC104887630) | -2.24001417 | 0.000348718 | 0.017777495 |
| XM_010693624.2 | 104905171 | BVRB_2g025900 | POX2 | proline dehydrogenase 2, mitochondrial (LOC104905171) | 2.930252587 | 1.14712E-09 | 3.90816E-07 |
| XM_010671902.2 | 104887286 | BVRB_1g004500 | GPAT8 | probable glycerol-3-phosphate acyltransferase 8 (LOC104887286) | 1.654188885 | 7.89591E-07 | 0.00013193 |
| XM_010673015.2 | 104888141 | - | PER5 | peroxidase 5-like | 1.337277475 | 0.000530067 | 0.024730071 |
| XM_010679016.2 | 104892989 | BVRB_5g106300 | KAO2 | ent-kaurenoic acid oxidase 2 (LOC104892989) | -4.01822947 | 0.001076356 | 0.042761585 |
| XM_010667546.2 | 104883093 | BVRB_8g202170 | FLS1 | flavonol synthase/flavanone 3-hydroxylase (LOC104883093) | 1.918033757 | 2.29143E-06 | 0.000309575 |
| XM_010673030.2 | 104888149 | BVRB_3g050950 | COMT | caffeic acid 3-O-methyltransferase (LOC104888149) | 1.216830354 | 0.000414006 | 0.020291221 |
| XM_010679303.2 | 104893204 | BVRB_1g010100 | ACO5 | 1-aminocyclopropane-1-carboxylate oxidase 5 (LOC104893204) | -1.845554556 | 3.34331E-05 | 0.002861558 |
| XM_010693636.2 | 104905185 | BVRB_1g016810 | NPC4 | non-specific phospholipase C4 (LOC104905185) | 1.381120151 | 0.000104226 | 0.00701961 |
| XM_010677311.2 | 104891604 | BVRB_4g087470 | BGLU18 | beta-glucosidase 18 (LOC104891604) | 1.669251582 | 0.001171978 | 0.045919478 |
| XM_010698104.2 | 104908933 | BVRB_7g179870 | PER21 | peroxidase 21 (LOC104908933) | 1.821748545 | 1.13032E-06 | 0.000173886 |
| XM_010672817.2 | 104888010 | BVRB_3g049860 | 4CL2 | 4-coumarate--CoA ligase 2 (LOC104888010) | 1.667267813 | 3.9304E-07 | 7.16728E-05 |
| XM_010697429.2 | 104908331 | BVRB_2g029890 | PER7 | peroxidase P7 (LOC104908331) | 2.713934908 | 2.42632E-05 | 0.002192223 |
| XM_010671488.2 | 104886934 | BVRB_2g036940 | CHI | chalcone--flavanone isomerase (LOC104886934) | 2.576304597 | 4.61777E-11 | 2.22423E-08 |
| XM_010682134.2 | 104895578 | BVRB_6g134460 | - | phosphoglycerate mutase-like protein 4 (LOC104895578) | 1.906141073 | 3.84892E-05 | 0.003215509 |
| XM_019249220.1 | 104892124 | BVRB_5g098350 | maoA | primary amine oxidase (LOC104892124) | -2.383443626 | 6.33999E-05 | 0.00475622 |
| XM_010676665.1 | 104891029 | BVRB_4g083150 | EMB3004 | bifunctional 3-dehydroquinate dehydratase/shikimate dehydrogenase, chloroplastic (LOC104891029) | 2.733071442 | 4.39936E-06 | 0.000540951 |
| XM_010669032.2 | 104884391 | BVRB_008110 | ACO1 | 1-aminocyclopropane-1-carboxylate oxidase 1 (LOC104884391) | -2.58555489 | 0.000399179 | 0.01974468 |
| XM_010691417.2 | 104903392 | BVRB_9g208880 | PER4 | peroxidase 4 (LOC104903392) | -2.235713175 | 0.000502692 | 0.023883666 |
| XM_010674949.2 | 104889670 | BVRB_1g007170 | DFRA | dihydroflavonol 4-reductase (LOC104889670) | 3.89551494 | 0.000275174 | 0.01495618 |
| XM_010667245.2 | 104882831 | BVRB_8g200320 | BGLU46 | beta-glucosidase 46 (LOC104882831) | 3.869491052 | 0.000141138 | 0.008920693 |
| XM_010668612.2 | 104884022 | BVRB_005400 | CAD6 | probable cinnamyl alcohol dehydrogenase 6 (LOC104884022) | 2.343318013 | 9.12805E-06 | 0.00100397 |
| XM_010683478.2 | 104896709 | BVRB_6g143630 | HISN1B | ATP phosphoribosyltransferase 2, chloroplastic (LOC104896709) | -1.609547804 | 1.41518E-06 | 0.00020985 |
| XM_010671086.2 | 104886619 | BVRB_2g034550 | SHT | spermidine hydroxycinnamoyl transferase (LOC104886619) | 1.578271848 | 6.4045E-05 | 0.004788236 |
| XM_010677911.2 | 104892088 | BVRB_5g098280 | maoA | primary amine oxidase (LOC104892088) | 2.886447039 | 9.25293E-13 | 6.59431E-10 |
| XM_010679602.2 | 104893499 | BVRB_5g110540 | BGLU13 | beta-glucosidase 13 (LOC104893499) | 2.121317093 | 7.84872E-08 | 1.70239E-05 |
| XM_010688539.2 | 104900996 | BVRB_8g186240 | PER42 | peroxidase 42 (LOC104900996) | 1.230041583 | 0.000348254 | 0.017775177 |
| XM_010686010.2 | 104898878 | BVRB_7g162990 | IPT3 | adenylate isopentenyltransferase 3, chloroplastic (LOC104898878) | -2.420606431 | 0.000586039 | 0.026821863 |
| XM_010681255.2 | 104894893 | BVRB_6g127540 | AMT | aminomethyltransferase, mitochondrial (LOC104894893) | -3.695230998 | 0.001242077 | 0.047716818 |
| XM_010673169.2 | 104888263 | BVRB_3g052140 | GLT1 | glutamate synthase 1 [NADH], chloroplastic (LOC104888263) | -1.207821297 | 0.000152854 | 0.009464186 |
| XM_010671471.1 | 104886918 | BVRB_2g036880 | CHI | chalcone--flavanone isomerase (LOC104886918) | 2.746736996 | 3.85324E-07 | 7.08205E-05 |
| XM_010682410.1 | 104895799 | - | PPO | polyphenol oxidase, chloroplastic (LOC104895799) | -3.440868736 | 2.91566E-07 | 5.6098E-05 |
| XM_010690065.2 | 104902333 | BVRB_8g197810 | BCAT2 | branched-chain-amino-acid aminotransferase 2, chloroplastic (LOC104902333) | 2.795326126 | 0.000216391 | 0.012449056 |
| XM_010678992.2 | 104892966 | BVRB_5g106570 | PER20 | peroxidase 20 (LOC104892966) | 2.06879058 | 1.29677E-08 | 3.37943E-06 |
| XM_010676592.2 | 104890968 | BVRB_4g082400 | OASC | cysteine synthase (LOC104890968) | 4.294721708 | 0.000121071 | 0.007932283 |
| XM_010693692.2 | 104905231 | BVRB_1g017310 | CYP51 | obtusifoliol 14-alpha demethylase (LOC104905231) | -1.450990488 | 0.001231455 | 0.04746331 |
| XM_010678655.1 | 104892666 | BVRB_5g099510 | PGD2 | 6-phosphogluconate dehydrogenase, decarboxylating 2, chloroplastic (LOC104892666) | -1.397641348 | 4.86663E-05 | 0.003853684 |
| XM_010677915.2 | 104892090 | BVRB_5g098290 | maoA | primary amine oxidase (LOC104892090) | 6.70197823 | 4.86653E-55 | 1.69944E-50 |
| XM_010687685.2 | 104900294 | BVRB_7g175860 | PER57 | peroxidase 57 (LOC104900294) | 3.232860417 | 7.59359E-06 | 0.000868037 |
| XM_010687686.2 | 104900295 | BVRB_7g175870 | PER57 | peroxidase 57 (LOC104900295) | 2.511889917 | 3.80273E-06 | 0.000482015 |
| XM_010671185.2 | 104886705 | BVRB_2g035170 | CEPT1 | choline/ethanolaminephosphotransferase 1 (LOC104886705) | #NAME? | 1.72643E-05 | 0.001674688 |
| XM_010667756.2 | 104883267 | BVRB_9g225600 | SAT | stemmadenine O-acetyltransferase (LOC104883267) | -1.374236569 | 0.001072738 | 0.042666373 |
| XM_010697796.1 | 104908662 | BVRB_7g177530 | PER57 | peroxidase 57 (LOC104908662) | -1.579802823 | 0.000119024 | 0.007864571 |
| XM_010671128.2 | 104886661 | BVRB_2g034560 | SHT | spermidine hydroxycinnamoyl transferase (LOC104886661) | 1.924698634 | 4.62026E-05 | 0.003698958 |
| XM_010697362.2 | 104908267 | BVRB_2g029810 | PER7 | peroxidase P7 (LOC104908267) | 2.894408154 | 1.71328E-07 | 3.42826E-05 |
| XM_010685251.2 | 104898208 | BVRB_7g157620 | PNC1 | cationic peroxidase 1 (LOC104898208) | 2.478310182 | 8.86303E-07 | 0.000142302 |
| XM_010674488.2 | 104889293 | BVRB_3g061430 | HST | shikimate O-hydroxycinnamoyltransferase (LOC104889293) | 1.560720316 | 0.0009408 | 0.038674144 |
| XM_010687683.2 | 104900293 | BVRB_7g175850 | DAPA | 4-hydroxy-tetrahydrodipicolinate synthase, chloroplastic (LOC104900293) | 6.529366821 | 3.11939E-05 | 0.002696341 |
| XM_010689693.2 | 104902035 | BVRB_8g194440 | PER25 | peroxidase 25 (LOC104902035) | -1.183409811 | 0.000115345 | 0.007657722 |
| XM_010672763.2 | 104887972 | BVRB_3g049400 | COMT | caffeic acid 3-O-methyltransferase (LOC104887972) | 2.049425659 | 3.47534E-05 | 0.002949269 |
| XM_010692669.2 | 104904425 | BVRB_9g218200 | AOS | allene oxide synthase (LOC104904425) | 2.721630649 | 0.000262801 | 0.014429651 |
| XM_010694743.2 | 104906043 | BVRB_2g045590 | DAPB2 | 4-hydroxy-tetrahydrodipicolinate reductase 2, chloroplastic (LOC104906043) | #NAME? | 0.000379665 | 0.019015292 |
| XM_010683011.2 | 104896291 | - | PER11 | peroxidase 11(LOC104896291) | 1.823996703 | 7.51738E-08 | 1.64586E-05 |
| XM_010668419.2 | 104883854 | BVRB_004050 | NCED2 | 9-cis-epoxycarotenoid dioxygenase NCED2, chloroplastic (LOC104883854) | 5.266678971 | 1.53854E-05 | 0.001515578 |
| XM_010681586.2 | 104895152 | BVRB_1g010750 | ADH2 | alcohol dehydrogenase 2 (LOC104895152) | -3.711434384 | 0.000245268 | 0.013749193 |
| XM_010675264.2 | 104889925 | - | BGLU13 | beta-glucosidase 13 (LOC104889925) | 3.86371059 | 0.000311892 | 0.01639064 |
| XM_010675265.2 | 104889926 | - | - | - | 2.01597039 | 0.000257211 | 0.014212114 |
| XM_019252566.1 | 104908274 | BVRB_2g029820 | PER7 | peroxidase P7 (LOC104908274) | 3.881413581 | 2.34628E-05 | 0.002144877 |
| XM_010693891.2 | 104905365 | BVRB_1g018740 | CHS3 | chalcone synthase 3 (LOC104905365) | 7.478293343 | 8.07373E-35 | 5.63885E-31 |
| XM_010685248.2 | 104898205 | BVRB_7g157590 | PNC1 | cationic peroxidase 1 (LOC104898205) | 2.747519058 | 2.18241E-09 | 6.92835E-07 |
| XM_010693893.2 | 104905367 | BVRB_1g018770 | CHS | chalcone synthase (LOC104905367) | 3.102508381 | 2.62784E-13 | 2.08561E-10 |
| XM_010693892.2 | 104905366 | BVRB_1g018750 | CHS3 | chalcone synthase 3 (LOC104905366) | 6.548582197 | 3.85753E-24 | 1.17138E-20 |
| XM_010676222.2 | 104890653 | BVRB_4g079730 | PER5 | peroxidase 5 (LOC104890653) | 3.180036258 | 1.38721E-14 | 1.3272E-11 |
| XM_010678672.2 | 104892681 | BVRB_1g009560 | gpmA1 | 2,3-bisphosphoglycerate-dependent phosphoglycerate mutase 1 (LOC104892681) | -1.635351992 | 2.41292E-05 | 0.002185778 |
| XM_010687230.2 | 104899938 | BVRB_7g171950 | OPR11 | putative 12-oxophytodienoate reductase 11 (LOC104899938) | 2.12010263 | 0.000410661 | 0.020183925 |
| XM_010687223.2 | 104899932 | BVRB_7g171880 | ASN1 | asparagine synthetase [glutamine-hydrolyzing] 1 (LOC104899932) | 2.710513029 | 0.00017685 | 0.01062044 |
| XM_010684275.2 | 104897406 | BVRB_6g149060 | ALDH2B7 | aldehyde dehydrogenase family 2 member B7, mitochondrial (LOC104897406) | -1.107775377 | 0.000871711 | 0.036490403 |
| XM_010694829.2 | 104906111 | BVRB_2g026880 | CYP735A1 | cytokinin hydroxylase (LOC104906111) | -3.748425054 | 3.9543E-17 | 6.736E-14 |
| XM_010694647.2 | 104905974 | BVRB_2g044780 | KCS10 | 3-ketoacyl-CoA synthase 10 (LOC104905974) | 2.694785201 | 1.74843E-13 | 1.43663E-10 |
| XM_010692633.2 | 104904397 | BVRB_9g217890 | LPAT5 | probable 1-acyl-sn-glycerol-3-phosphate acyltransferase 5 (LOC104904397) | 4.741877688 | 0.00069684 | 0.030661613 |
| XM_010696649.2 | 104907682 | BVRB_4g096540 | GLY1 | low-specificity L-threonine aldolase 1 (LOC104907682) | 2.251801408 | 1.49286E-06 | 0.000218583 |
| XM_010672411.2 | 104887704 | BVRB_2g043300 | KCS11 | 3-ketoacyl-CoA synthase 11 (LOC104887704) | 2.008308313 | 2.4801E-05 | 0.002229278 |
| XM_010684411.2 | 104897526 | BVRB_6g150380 | G6PD | glucose-6-phosphate 1-dehydrogenase, chloroplastic (LOC104897526) | -2.24472029 | 2.45901E-10 | 9.70294E-08 |
| XM_010685258.2 | 104898212 | BVRB_7g157660 | - | alanine--glyoxylate aminotransferase 2 homolog 3, mitochondrial (LOC104898212) | 1.261863293 | 0.000577698 | 0.026492174 |
| XM_010685262.2 | 104898217 | BVRB_7g157710 | KCS11 | 3-ketoacyl-CoA synthase 11 (LOC104898217) | 2.723529959 | 2.36143E-07 | 4.58861E-05 |
| XM_010684569.2 | 104897637 | BVRB_6g151550 | GAPG | glyceraldehyde-3-phosphate dehydrogenase, cytosolic (LOC104897637) | -1.570419815 | 0.000144702 | 0.009112958 |
| XM_010674599.2 | 104889392 | BVRB_3g061940 | GPDH1 | glycerol-3-phosphate dehydrogenase [NAD(+)] GPDHC1, cytosolic (LOC104889392) | 1.848984619 | 4.25198E-06 | 0.000524676 |
|  |  |  |  | **Cutin, suberine and wax biosynthesis** |  |  |  |
| XM_010681683.2 | 104895231 | BVRB_6g131050 | HHT1 | omega-hydroxypalmitate O-feruloyl transferase (LOC104895231) | 2.104663849 | 2.3584E-09 | 7.45319E-07 |
| XM_010690388.2 | 104902574 | BVRB_9g202950 | CYP86B1 | cytochrome P450 86B1 (LOC104902574) | 1.937451692 | 5.08664E-06 | 0.000614639 |
| XM_010687741.2 | 104900337 | BVRB_7g176320 | FAR | alcohol-forming fatty acyl-CoA reductase (LOC104900337) | 3.819812574 | 4.16812E-21 | 9.09718E-18 |
| XM_010671882.2 | 104887268 | BVRB_1g004480 | CYP86A8 | cytochrome P450 86A8 (LOC104887268) | 1.849382568 | 7.33994E-06 | 0.00084454 |
| XM_010678291.1 | 104892372 | BVRB_5g101370 | CYP86B1 | cytochrome P450 86B1 (LOC104892372) | -2.572279446 | 0.00012585 | 0.008176383 |
| XM_010687294.1 | 104899987 | BVRB_7g172510 | CYP86A1 | cytochrome P450 86A1 (LOC104899987) | 1.863155593 | 8.4382E-07 | 0.000137696 |
| XM_010679975.2 | 104893839 | BVRB_5g112740 | HHT1 | omega-hydroxypalmitate O-feruloyl transferase (LOC104893839) | 3.633514811 | 7.99479E-25 | 2.65891E-21 |
| XM_010689193.1 | 104901603 | BVRB_8g190320 | FAR3 | fatty acyl-CoA reductase 3 (LOC104901603) | 3.392123097 | 1.12177E-10 | 4.8362E-08 |
